# A multistep continuous flow synthesis machine for the preparation of pyrazoles *via* a metal-free amine-redox process†
†Raw spectra can be found at https://www.repository.cam.ac.uk/handle/1810/252343.
‡
‡Electronic supplementary information (ESI) available. See DOI: 10.1039/c5re00082c



**DOI:** 10.1039/c5re00082c

**Published:** 2016-01-07

**Authors:** Jian-Siang Poh, Duncan L. Browne, Steven V. Ley

**Affiliations:** a Department of Chemistry , University of Cambridge , Lensfield Road , Cambridge , CB2 1EW , UK; b School of Chemistry , Cardiff University , Main Building, Park Place , CF10 3AT , UK . Email: dlbrowne@cardiff.ac.uk

## Abstract

A versatile multistep continuous flow setup is reported for the four-step conversion of anilines into pyrazole products. The synthesis machine incorporates the use of amine-redox chemistry through diazotization and a metal-free vitamin C mediated reduction. The machine can be used for the synthesis of an array of analogues or the scale up of an individual target.

## Abstract 2

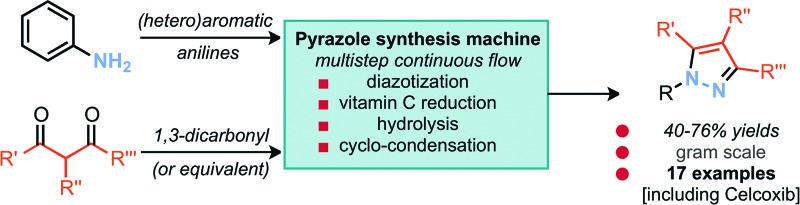
A versatile multistep continuous flow setup is reported for the four-step conversion of anilines into pyrazole products.

## Introduction

**Fig. 1:**
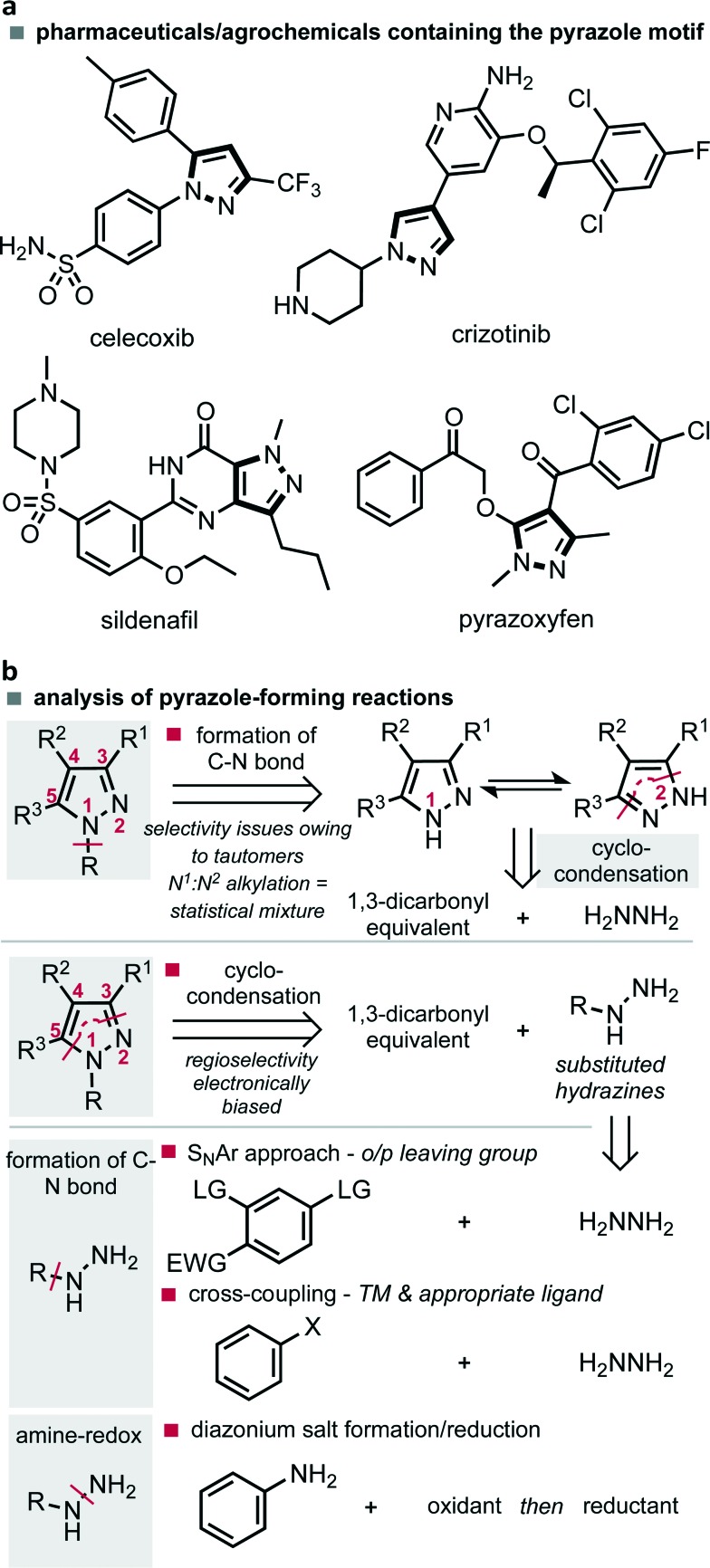
a Examples of pyrazole scaffolds. b Synthetic approaches towards pyrazole scaffolds.

The synthesis of heterocyclic compounds continues to serve as a cornerstone for the preparation of materials necessary for our modern society. The way in which such materials are constructed is therefore an important area of research whereby ease of diversity, simplicity of scale up and sustainability of the method represent key factors in the future advancement of this established field. In the case of pyrazoles, five-membered heterocycles featuring two adjacent nitrogen atoms, their functionality is characterized by their appearance in materials, agrochemicals and pharmaceuticals, examples of which are celecoxib (**1**), crizotinib, sildenafil and pyrazoxyfen (Fig. 1a).^1^ At scale these compounds are most commonly prepared by the cyclo-condensation of hydrazines with 1,3-dicarbonyl compounds or their equivalents. This can be achieved by either the cyclo-condensation with simple hydrazine followed by a non-selective alkylation of a tautomeric mixture, or by appropriate substitution of hydrazine followed by an electronically controlled regioselective cyclo-condensation (Fig. 1b). The latter approach is often preferred as it leads to more selective processes. However, both strategies require the preparation and storage of hydrazines on scale, a practice which represents a significant safety risk, economic investment and is applicable to a relatively limited substrate scope.

Substituted hydrazines are often prepared by nucleophilic aromatic substitution (S_N_Ar) of the appropriately activated benzene systems, or more recently, utilising palladium-catalysed cross-coupling methods with hydrazine. In the latter case, the scope has been increased by the development of important ligands that permit a difficult reductive elimination step.^2^ Although this cross-coupling approach may have some way to go before its application on scale becomes economically and environmentally sustainable, some headway has been made with the development of a continuous flow approach.^3^ An alternative option, which permits access to a vast range of substituted hydrazines centres on the use of amine-redox chemistry. This method consists of oxidation through diazotization, followed by reduction of the N–N triple bond, commonly with tin(ii) chloride (Fig. 1b).^4^

However, in recent years there has been an interest in moving towards more sustainable reductants, those that can be washed away from the product through simple aqueous extraction where ascorbic acid or sodium sulfite serve as reductant, including our own previous mechanistic work and flow proof-of-concept.^5^ Here we describe the first report of an end-to-end multistep^6^ continuous flow process^7^ for the amine-redox cycle followed by hydrolysis of the hydrazine surrogate and ensuing cyclo-condensation, a process characterised by low inventories of both diazonium salt and hydrazine at any one time and the use of vitamin C as reductant.^8^

## Results and discussion

**Fig. 2:**
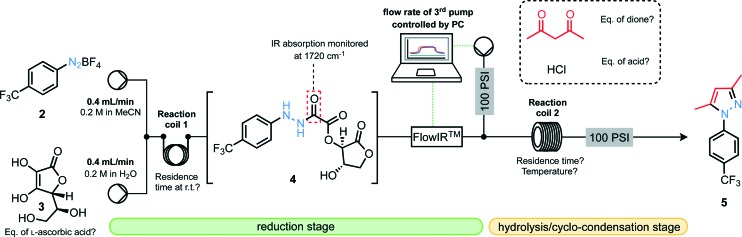
Equipment setup for segmented flow reduction/hydrolysis/cyclo-condensation sequence.

Our first experiments towards this goal commenced with the development of compatible flow conditions for the reduction stage followed by hydrolysis and cyclo-condensation (Fig. 2). Aiming for *N*-arylated pyrazoles, we tested the performance of the setup by injecting 0.8 mmol segments of 4-trifluoromethylbenzenediazonium tetrafluoroborate (**2**, 0.2 M in MeCN) and l-ascorbic acid (**3**, 0.2 M in H_2_O) which were combined at a T-piece and passed through a preliminary reactor coil where the reduction stage could take place. This solution then passed through an in-line flow IR device which served two purposes: (i) to monitor the presence of the desired carbonyl stretching frequency resulting from the oxamate moiety as well as to monitor levels of diazonium compound still on-stream; (ii) to actively control the flow rate of a third pump^9^ delivering both pentane-2,4-dione and the hydrochloric acid necessary for oxamate cleavage so that stoichiometries could be matched with the flowing segment, thus delivering matched concentration profiles.

Once combined with the acid and the dione, the stream then passed through a heated coil before emerging at the output through a 100 psi back-pressure regulator. The flow procedure developed here offers advantages over batch procedures, allowing rapid screening of reaction conditions with minimal operator input, as well as the *in situ* formation of unstable and/or explosive intermediates (hydrazides and hydrazines) to minimize operator exposure.

With an appropriate apparatus setup to optimize the three-step reduction/hydrolysis/cyclo-condensation sequence established, various parameters were investigated (Table 1). Notably, it was found that the final yield of pyrazole was highly dependent on the residence time for the reduction step with *t*_R_ = 6 min appearing to be optimal; a decreased residence time does not allow the reduction to proceed to completion, whereas increased residence times result in greater decomposition of hydrazide **4**. We are mindful here of recent reports on the catalytic generation of radical intermediates through the action of ascorbic acid on diazonium salts as a likely competing pathway.^10^ There is also an optimum temperature for reactor coil 2 around 140 °C, where higher than this seemed to result in reduced yields of pyrazole products. Optimal conditions were found (Table 1, entry 4), providing pyrazole **5** in 69% yield after isolation.

**Table 1:** Optimization of conditions for segmented flow reduction/hydrolysis/cyclo-condensation sequence

Entry	Coil 1, *t*_R_/min	Coil 2, *T*/°C	Equiv. of HCl	Yield^*a*^ (%)
1	2.5	100	5.5	21
2	2.5	140	5.5	46
3^*b*^	2.5	140	5.5	45
4	6	140	5.5	71 (69)
5	10	140	5.5	67
6	18	140	5.5	62
7	35	140	5.5	58
8	6	100	5.5	21
9	6	120	5.5	55
10	6	160	5.5	53
11^*c*^	6	140	5.5	70
12^*d*^	6	140	5.5	71
13	6	140	0	35
14	6	140	2.2	68
15	6	140	11	58

^*a*^Yield determined from quantitative ^1^H NMR analysis with DMF as internal standard, yield of isolated product in parentheses.

^*b*^63 minute residence time in reactor coil 2.

^*c*^2.2 equivalents of dione used.

^*d*^2 equivalents of ascorbic acid used.

**Fig. 3:**
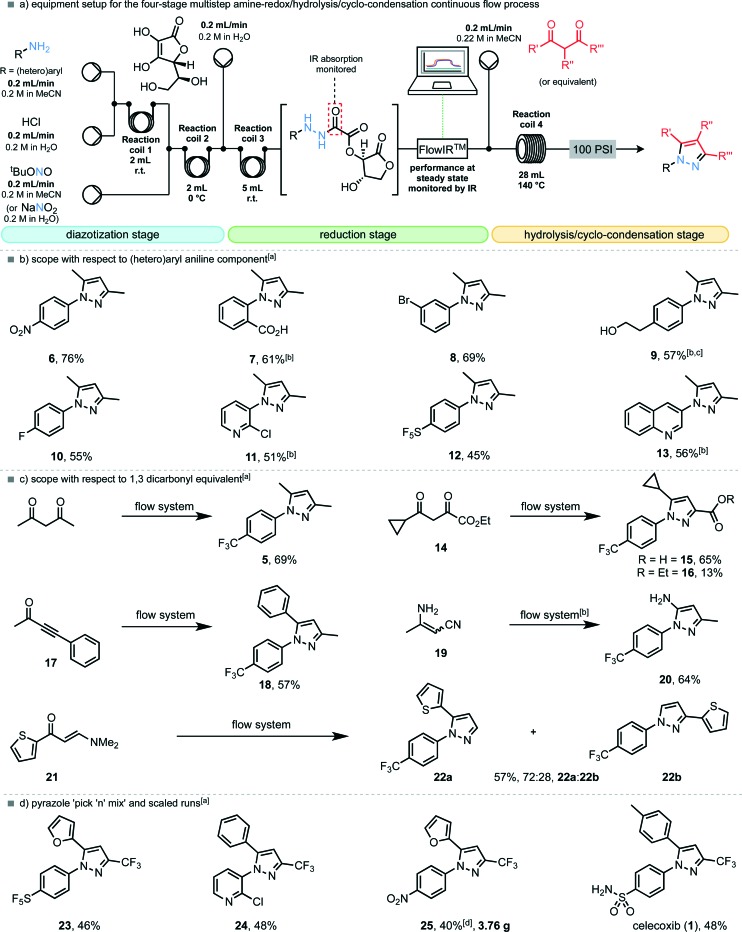
Equipment setup and scope for the continuous flow diazotization/reduction/hydrolysis/cyclo-condensation sequence. ^a^ Entries run on 4.8 mmol scale with respect to aniline unless otherwise stated, yields represent isolated product. ^b^ Using NaNO_2_ in H_2_O as diazotizing agent. ^c^ Using a 15 mL reaction coil at 30 °C for the reduction module. ^d^ Run on 28.8 mmol scale over 12 hours.

Next we sought to assess the scope of the reaction, but with the following modifications to the flow procedure: (i) it would be preferable for the diazonium salts to also be produced *in situ* from their corresponding anilines, thus avoiding the isolation and handling of additional explosive species; (ii) a transformation of the procedure from segmented flow to continuous flow would allow safe scale-up to provide gram-quantities of *N*-arylated pyrazoles. A ‘diazotization module’ was easily incorporated into the existing flow set-up to replace the diazonium tetrafluoroborate input stream, resulting in a four-step telescoped flow procedure (Fig. 3a).

In order to assess whether the new flow set-up with the *in situ* generation of diazonium chloride salts (as opposed to the tetrafluoroborate salts) was compatible with the existing reduction, hydrolysis and condensation steps, we subjected 4-trifluoromethylaniline to the fully telescoped process, undergoing diazotization with 1 equiv. of *t*-butyl nitrite, reduction with 1 equiv. of l-ascorbic acid and condensation with 1.1 equiv. of pentane-2,4-dione, run under continuous flow conditions for 2 h. Pleasingly, the desired pyrazole **5** was isolated in 69% yield over four steps, in good agreement with the previously optimized results. Scope of the reaction and flow process with respect to anilines was then explored. In general, most electron-withdrawing substituents on the anilines were tolerated (Fig. 3b). Notably, anthranilic acid, whose diazonium derivative is known to decompose (explosively, if uncontrolled) to benzyne could be effectively employed in the flow process and provided pyrazole **7** in 61% yield. Conversion of the 4-pentafluorosulfanyl amine to pyrazole **12** *via* its corresponding, previously unknown, hydrazine proceeded in a moderate 45% yield. Electron-rich systems such as 4-methoxyaniline were not tolerated in the reduction step, but heteroaromatic ring systems were applicable; both 3-amino-2-chloropyridine and 3-aminoquinoline provided the desired pyrazoles (**11** and **13**) in good yields.

Next, a variety of 1,3-dicarbonyl compounds or equivalents were examined in the telescoped multistep process (Fig. 3c). The unsymmetrical 1,3,4-tricarbonyl compound **14** provided pyrazoles **15** (ref. 11) and **16** as single regioisomers, in a combined yield of 78%. Acetylenic ketone **17** and 3-aminocrotononitrile (**19**) were also converted to pyrazole **18** and **20** (ref. 11) respectively as single regioisomers within the standard operating conditions of this pyrazole synthesising machine. Enaminone **21** was also an effective substrate leading to 57% yield of a 3 : 1 mixture of pyrazole regioisomers (**22a** and **22b**).

As a further illustration of the utility of the telescoped procedure, perhaps as a machine to aid possible drug discovery, a selection of relevant 1,3-diketones were crossed with aniline starting materials to yield pyrazole analogues of celecoxib (**1**) as a product output (Fig. 3d). Furan-bearing, pentafluorosulfanyl pyrazole **23** was furnished in 46% yield, whereas the *N*-pyridyl variant (**24**) was isolated in 48% yield. A scale-up experiment where the machine was running for a 12 hour period demonstrated the synthesis of the 4-nitrophenyl analogue **25** in 40% yield, affording 3.76 g of the isolated pyrazole product. Finally, the appropriate sulfanilamide was diazotized, reduced with l-ascorbic acid, hydrolyzed and condensed with the corresponding dione to provide the COX-2 selective non-steroidal anti-inflammatory drug celecoxib (**1**) in 48% yield over four continuous steps.

## Conclusions

In summary, we have reported a metal-free continuous flow method for the generation of a variety of *N*-arylated pyrazoles *via* amine-redox chemistry. The telescoped flow process offers a distinct advantage over corresponding batch procedures due to the *in situ* formation and use of several reactive intermediates. Critically, this end-to-end style of synthesis avoids the stock-piling and storage of hazardous drug intermediates (diazonium salts and hydrazines) by advancing them to the next step as they are produced. The machine, and conditions/parameters reported here are applicable to a relatively broad range of amine and 1,3-dicarbonyl materials. Further investigations into the handling and use of reactive intermediates in flow as well as the development of multistep machines to support the synthesis and discovery process are currently ongoing in our laboratories.
