# Supplementary material for: A multistep continuous flow synthesis machine for the preparation of pyrazoles via a metal-free amine-redox process
Source: React Chem Eng. 2016 Jan 7;1(1):101–5. doi: 10.1039/c5re00082c (PMC4906367; doi:10.1039/c5re00082c)
Supplement: Supplementary file 1 [file RE-001-C5RE00082C-s001.pdf]

Electronic Supplementary Material (ESI) for Reaction Chemistry & Engineering.
This journal is © The Royal Society of Chemistry 2015

Supporting Information

A Multistep Continuous Flow Synthesis Machine for the Preparation of Pyrazoles via a Metal-Free Amine-Redox Process

Jian-Siang Poh,[a] Duncan L. Browne*,[a, b] and Steven V. Ley[a]

[a] Whiffen Laboratory, Department of Chemistry, University of Cambridge, Lensfield Road, CB2 1EW, Cambridge, UK
[b] School of Chemistry, Cardiff University, Main Building, Park Place, CF10 3AT, Cardiff, UK

Contents

1. General experimental details ... 3
2. Synthetic procedures and characterisation for pyrazoles ... 4
3. NMR spectra ... 21
4. References ... 51

1. General experimental details

All batch reactions were performed using oven-dried glassware (200 °C) under an atmosphere of argon unless otherwise stated. All flow reactions were performed using a Uniqsis FlowSyn platform,[1] Vapourtec E-series system[2] and a Knauer Smartline Pump 100.[3] In-line IR spectroscopy was performed using a Mettler Toledo FlowIR™ device equipped with a SiComp (silicon) head.[4] Solvents were freshly distilled over sodium benzophenone ketyl (Et_2O) or calcium hydride (MeCN, CH_2Cl_2, hexane and EtOAc). All reagents were obtained from commercial sources and used without further purification.

Flash column chromatography was performed using high-purity grade silica gel (Merck grade 9385) with a pore size 60 Å and 230–400 mesh particle size under air pressure. Analytical thin layer chromatography (TLC) was performed using silica gel 60 F_{254} pre-coated glass backed plates and visualized by ultraviolet radiation (254 nm) and/or potassium permanganate solution as appropriate.

^{1}H NMR spectra were recorded on a 400 MHz DPX-400 Dual Spectrometer, 500 MHz DCH Cryoprobe Spectrometer or a 600 MHz Avance 600 BBI Spectrometer as indicated. Chemical shifts are reported in ppm with the resonance resulting from incomplete deuteration of the solvent as the internal standard ($CDCl_3$: 7.26 ppm; d_3-MeCN: 1.94 ppm, qn). ^{13}C NMR spectra were recorded the same spectrometers with complete proton decoupling. Chemical shifts are reported in ppm with the solvent resonance as the internal standard ($^{13}CDCl_3$: 77.16 ppm, t; d_3-MeCN: 1.32 ppm (methyl), septet). ^{19}F NMR spectra were recorded on a 376 MHz Avance III HD Spectrometer. Chemical shifts are reported in ppm with $CFCl_3$ as the external standard ($CFCl_3$: 0.00 ppm). Data are reported as follows: chemical shift δ/ppm, integration (^{1}H only), multiplicity (s = singlet, d = doublet, t = triplet, q = quartet, qn = quintet, br = broad, m = multiplet or combinations thereof; ^{13}C signals are singlets unless otherwise stated), coupling constants *J* in Hz, assignment. Spectra are assigned as fully as possible, using ^{1}H-COSY, DEPT-135, HMQC and HMBC where appropriate to facilitate structural determination. Signals that cannot be unambiguously assigned are reported with all possible assignments separated by a slash (e.g. H_1/H_2). Multiple signals arising from diastereotopic positions are suffixed alphabetically (e.g. H_{1a}, H_{1b}). Overlapping signals that cannot be resolved are reported with their assignments denoted in list format (e.g. H_1, H_2 and H_3). ^{1}H NMR signals are reported to 2 decimal places and ^{13}C signals to 1 decimal place unless rounding would produce a value identical to another signal. In this case, an additional decimal place is reported for both signals concerned.

Infrared spectra were recorded neat as thin films on a Perkin-Elmer Spectrum One FTIR spectrometer and selected peaks are reported (s = strong, m = medium, w = weak, br = broad).

High resolution mass spectrometry (HRMS) was performed using positive electrospray ionisation (ESI+), on either a Waters Micromass LCT Premier spectrometer or performed by the Mass Spectrometry Service for the Chemistry Department at the University of Cambridge. All *m/z* values are reported to 4 decimal places and are within ± 5 ppm of theoretical values.

Melting points were collected using a Stanford Research Systems OptiMelt Automated Melting Point System using a gradient of 1.0 °C per min.

Elemental composition microanalysis was performed by the Microanalytical Laboratories at the Department of Chemistry, University of Cambridge.

2. Synthetic procedures and characterisation for pyrazoles

Procedure for producing calibration curve graph:

To a 10 mL volumetric flask was added 4-trifluoromethyldiazonium tetrafluoroborate (0.260 g, 1.0 mmol) and L-ascorbic acid (0.176 g, 1.0 mmol). The reaction was started with the addition of MeCN/H_2O (1:1) up to the mark (producing a final adduct concentration of 0.1 M), whereupon the homogeneous mixture immediately turned yellow. A magnetic stirrer bar was added and the mixture stirred at r.t. until completion of the reaction (monitored by ^{1}H NMR, approx. 30 min). Into a Mettler Toledo SiComp FlowIR™ flow cell was injected (using a syringe adapter) 0.5 mL of MeCN/H_2O (1:1) to act as the reference spectrum. A 0.5 mL aliquot from the reaction mixture was then injected into the flow cell and the absorbance measured at 1720 cm^{-1}. Three serial dilutions (0.05 M, 0.025 M, 0.0125 M) were made and their corresponding absorbances measured.

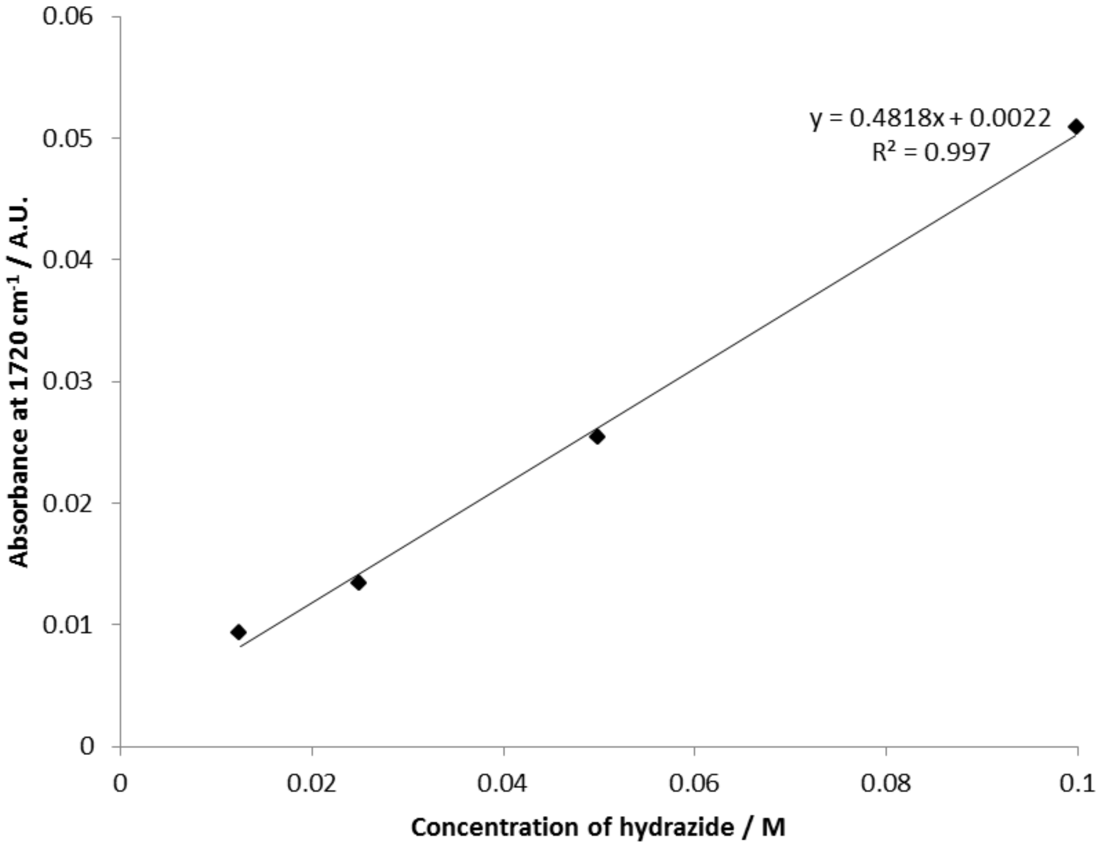

General procedure for segmented flow optimisation reactions:

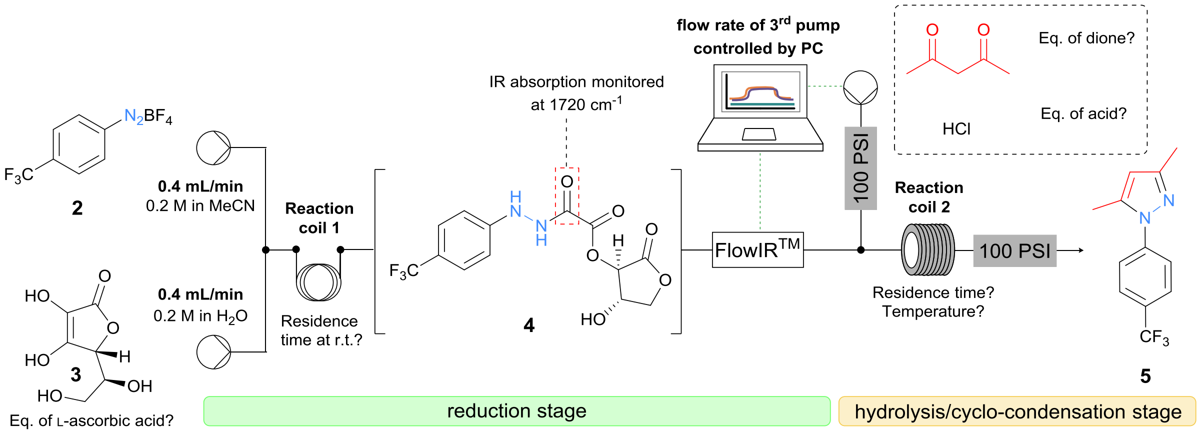

The Uniqsis FlowSyn was primed with MeCN in solvent line A, H_2O in solvent line B, a freshly prepared solution of 4-trifluoromethyldiazonium tetrafluoroborate (0.2 M in MeCN) in reagent line A and a solution of L-ascorbic acid (0.2 M in H_2O) in reagent line B. Solvent lines A and B were each run at 0.4 mL/min which were then mixed in a T-piece, directed into a reaction coil held at r.t. followed by the Mettler Toledo FlowIR^{TM} silicon flow cell configured to obtain ν_{max} values at 1720 cm^{-1} on the iC IR software; the zero reference spectrum was set to the 1:1 MeCN/H_2O mixture. The absorbance readings obtained every 15 s were output into Microsoft Excel. The flow stream was then directed into another T-piece connected to a Knauer Smartline Pump 100 fitted with a 100 PSI BPR, pre-primed up to the T-piece with a solution of pentane-2,4-dione (0.2 M in 1:1 MeCN/H_2O) and HCl (1.0 M in 1:1 MeCN/H_2O). The Knauer pump was controlled with a RS232 serial cable using LabVIEW to automatically dispense the dione/HCl solution at a flow rate dependent on the IR absorbance reading from the Excel spreadsheet.[5] (The correlation coefficient was calculated to be 9132 μL/min, using a flow rate of 0.8 mL/min prior to the third pump, 1.1 equivalents of pentane-2,4-dione and a 0.2 M solution of pentane-2,4-dione.) The output stream was directed into a 28 mL reactor coil held at 140 °C, followed by a 100 PSI BPR then the collection vessel. The Uniqsis FlowSyn was then switched from solvent lines A and B to reagent lines A and B and run for 10 min, then switched back to solvent. The output mixture was treated with an aqueous solution of saturated $NaHCO_3$ (10 mL), extracted with diethyl ether (3 × 20 mL). The combined organic extracts were washed with brine (20 mL), dried ($MgSO_4$) and evaporated under reduced pressure to yield the crude product.

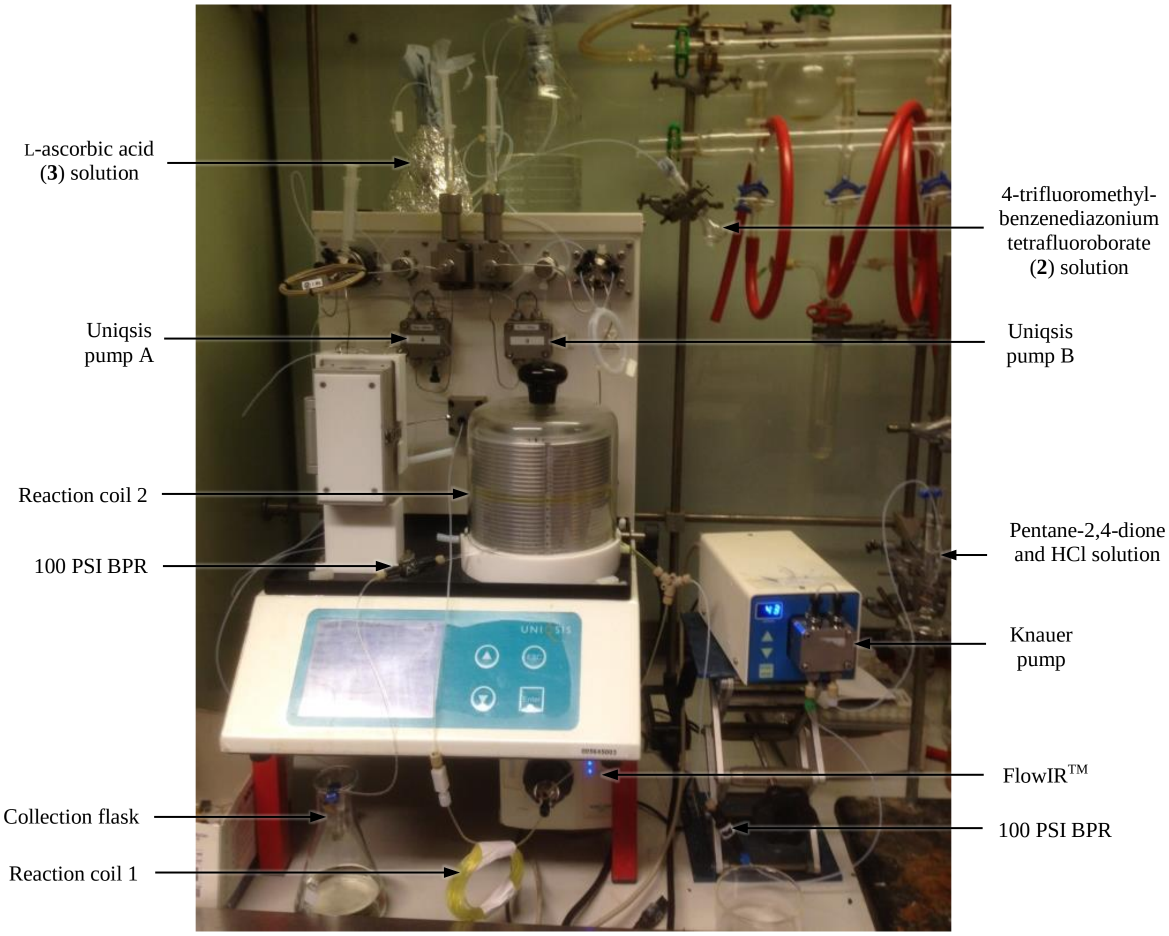

Figure 1: Reaction set-up for optimisation studies under segmented flow conditions.

General procedure for the continuous flow telescoped synthesis of pyrazoles:

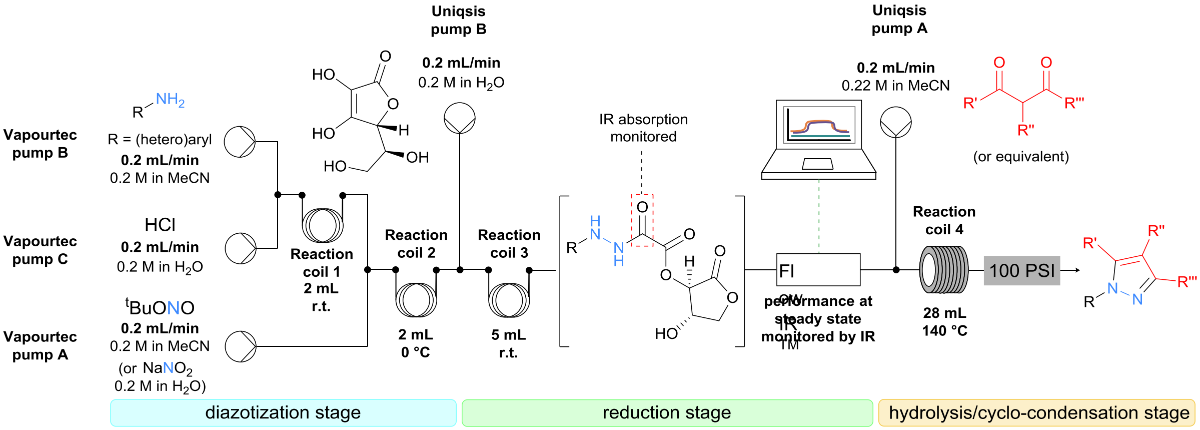

The Vapourtec E-series was primed with MeCN in solvent lines A and B, H_2O in solvent line C, a solution of *tert*-butyl nitrite (0.2 M in MeCN) in reagent line A, a solution of the substituted aniline (0.2 M in MeCN) in reagent line B and a solution of HCl (1.0 M in H_2O) in reagent line C. The Uniqsis FlowSyn was primed with MeCN in solvent line A, H_2O in solvent line B, a solution of the 1,3-dicarbonyl compound or equivalent (0.22 M in MeCN) in reagent line A and a solution of L-ascorbic acid (0.2 M in H_2O) in reagent line B. The complete flow platform was set up as follows: Vapourtec lines B and C were directed into a T-piece followed by a 2 mL reaction coil held at r.t., a T-piece connected to Vapourtec line A, a 2 mL reaction coil held at 0 °C in an ice bath, a T-piece connected to Uniqsis line B, a 5 mL reaction coil held at r.t., the FlowIR^{TM} silicon flow cell configured to obtain IR absorbance values at 1700-1780 cm^{-1}, a T-piece connected to Uniqsis line A, a 28 mL reaction coil held at 140 °C and finally a 100 PSI BPR whose output was directed into the collection flask; each pump was run at 0.2 mL/min. Initially all input lines were set to solvent and were left to run for 30 min – during this time a zero reference spectrum was obtained. Vapourtec lines A, B and C were then switched from solvent to reagent, left to run for 15 min, then Uniqsis lines A and B were switched from solvent to reagent. The system was run at a pre-steady state flow set-up for a further 60 min. The output was then collected at steady state for 120 min, which was treated with an aqueous solution of saturated sodium $NaHCO_3$ (25 mL) and extracted with diethyl ether (3 × 50 mL). The combined organic extracts were washed with brine (50 mL), dried ($MgSO_4$) and evaporated under reduced pressure. The crude product was purified by silica gel column chromatography to afford the desired pyrazoles.

(**Shut down procedure:** Vapourtec lines A, B and C were switched from reagent back to solvent. Once a large drop in the absorbance was detected by the FlowIR^{TM} signalling the end of hydrazide formation, Uniqsis lines A and B were switched from reagent back to solvent. Solvent was further pumped for 60 min to ensure complete purging of reactants from the system, whereupon pumping was terminated.)

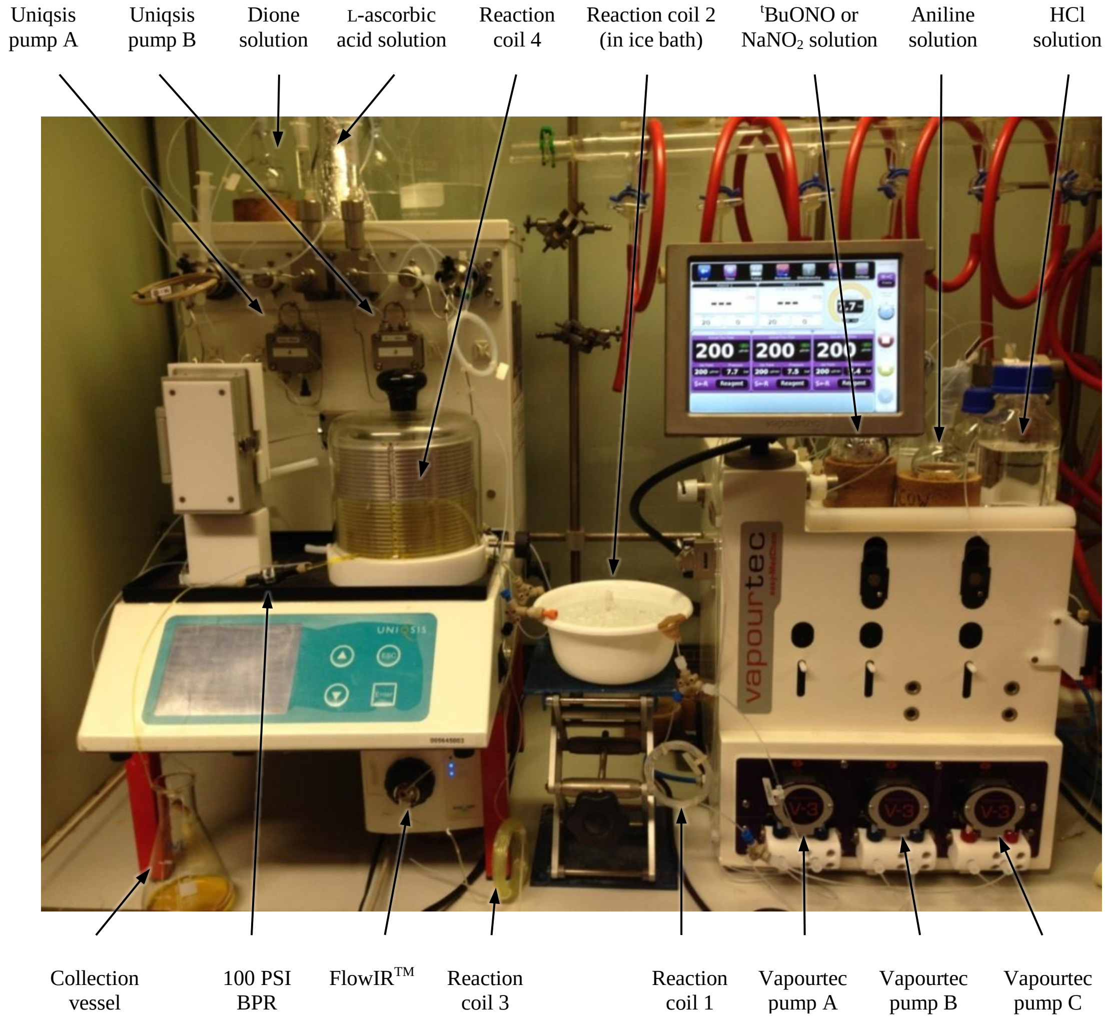

Figure 2: Reaction set-up for scope studies under continuous flow.

4-(5-(*p*-tolyl)-3-(trifluoromethyl)-1*H*-pyrazol-1-yl)benzenesulfonamide, celecoxib (1):

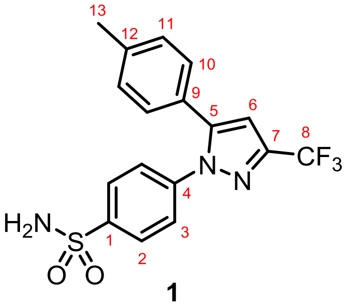

1

Isolated as an off-white amorphous solid (0.873 g, 2.3 mmol, 48%) after silica gel column chromatography (eluent: 30% EtOAc/hexane) following the general procedure for the telescoped synthesis of pyrazoles using sulfanilamide (0.827 g, 4.8 mmol) and 4,4,4-trifluoro-1-(*p*-tolyl)-1,3-butanedione (1.215 g, 5.3 mmol); m.p. 154-155 °C (lit. m.p.[6] 154-156 °C). Data is consistent with a reported example.[6]

^{1}H NMR (600 MHz, d$_3$-MeCN): δ 7.89-7.86 (m, 2 H, H_2), 7.49-7.46 (m, 2 H, H_3), 7.21-7.17 (m, 4 H, H_{10} and H_{11}), 6.92 (s, 1 H, H_6), 5.75 (s, 2 H, NH_2), 2.34 (s, 3 H, H_{13}).

^{13}C NMR (150 MHz, d$_3$-MeCN): δ 146.6 (C_5), 143.9 (C_4), 143.8 (q, $^{2}J_{CF}$ = 37.8 Hz, C_7), 143.1 (C_1), 140.8 (C_9), 130.4 (C_{10}), 129.9 (C_{11}), 128.1 (C_2), 126.9 (C_3), 126.8 (C_{12}), 122.5 (q, $^{1}J_{CF}$ = 266.4 Hz, C_8), 107.0 (q, $^{3}J_{CF}$ = 2.0 Hz, C_6), 21.3 (C_{13}).

^{19}F NMR (376 MHz, d$_3$-MeCN): δ -62.8 (s, CF_3).

FTIR (ν_{max}, cm^{-1}): 3338 (w, N-H stretch), 3231 (w, N-H stretch), 1594 (w), 1563 (w), 1498 (w), 1474 (w), 1446 (w), 1374 (w), 1346 (m), 1274 (m), 1229 (m), 1157 (s), 1132 (s), 1104 (m), 1016 (w), 981 (m), 970 (m), 904 (m), 845 (m), 801 (w), 791 (m), 760 (m).

HRMS (ESI): calculated for $C_{17}H_{15}N_3O_2F_3S$ $[M+H]^+$ 382.0837, found 382.0841.

R$_f$ = 0.23 (30% EtOAc/hexane).

Anal. calcd. for $C_{17}H_{14}N_3O_2F_3S$: C 53.56, H 3.70, N 11.02; found C 53.27, H 3.69, N 10.58.

3,5-dimethyl-1-(4-(trifluoromethyl)phenyl)-1*H*-pyrazole (5):

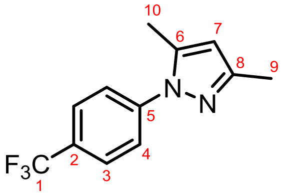

5

Isolated as a yellow oil (0.801 g, 3.3 mmol, 69%) after silica gel column chromatography (eluent: 10% EtOAc/hexane) following the general procedure for the telescoped synthesis of pyrazoles using 4-trifluoromethylaniline (0.773 g, 4.8 mmol) and pentane-2,4-dione (0.529 g, 5.3 mmol). Data is consistent with a reported example.[7]

^{1}H NMR (400 MHz, CDCl$_3$): δ 7.66 (d, $^{3}J_{HH}$ = 8.4 Hz, 2 H, H_3), 7.55 (d, $^{3}J_{HH}$ = 8.4 Hz, 2 H, H_4), 6.00 (s, 1 H, H_7), 2.31 (s, 3 H, H_9), 2.27 (s, 2 H, H_{10}).

^{13}C NMR (100 MHz, CDCl$_3$): δ 149.9 (C_8), 142.8 (q, $^{5}J_{CF}$ = 1.2 Hz, C_5), 139.5 (C_6), 128.7 (q, $^{2}J_{CF}$ = 32.7 Hz, C_2), 126.1 (q, $^{3}J_{CF}$ = 3.8 Hz, C_3), 124.0 (C_4), 123.9 (q, $^{1}J_{CF}$ = 270.7 Hz, C_1), 108.2 (C_7), 13.3 (C_9), 12.5 (C_{10}).

^{19}F NMR (376 MHz, CDCl$_3$): δ -62.4 (s, CF_3).

FTIR (ν_{max}, cm^{-1}): 1616 (m), 1560 (w), 1524 (m), 1417 (m), 1383 (m), 1366 (m), 1321 (s), 1164 (m), 1119 (s), 1104 (s), 1067 (s), 1035 (s), 1019 (m), 1012 (m), 974 (m), 844 (s), 787 (m), 758 (w).

HRMS (ESI): calculated for $C_{12}H_{12}N_2F_3$ $[M+H]^+$ 241.0953, found 241.0964.

R$_f$ = 0.30 (10% EtOAc/hexane).

Anal. calcd. for $C_{12}H_{11}N_2F_3$: C 60.00, H 4.61, N 11.66; found C 60.08, H 4.69, N 11.53.

Ethyl 5-cyclopropyl-1-(4-(trifluoromethyl)phenyl)-1*H*-pyrazole-3-carboxylate (16):

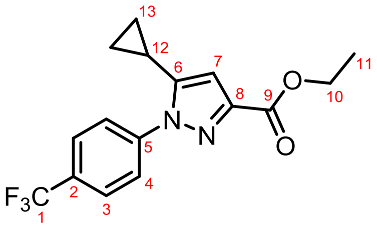

16

Isolated as a pale yellow amorphous solid (0.165 g, 0.5 mmol, 13%) following a modified version of the general procedure for the telescoped synthesis of pyrazoles using 4-trifluoromethylaniline (0.773 g, 4.8 mmol) and ethyl 4-cyclopropyl-2,4-dioxobutanoate (0.973 g, 5.3 mmol). The output was treated with an aqueous solution of sodium hydroxide (3 M in H_2O) until pH 10 and the aqueous layer extracted with CH_2Cl_2 (3 × 50 mL). The combined organic extracts were dried ($MgSO_4$), evaporated under reduced pressure and purified by silica gel column chromatography (eluent: 10% EtOAc/hexane); m.p. 72-73 °C.

^{1}H NMR (600 MHz, $CDCl_3$): δ 7.82 (d, $^3J_{HH}$ = 8.4 Hz, 2 H, H_3), 7.75 (d, $^3J_{HH}$ = 8.4 Hz, 2 H, H_4), 6.54 (s, 1 H, H_7), 4.41 (q, $^3J_{HH}$ = 7.1 Hz, 2 H, H_{10}), 1.81-1.76 (m, 1 H, H_{12}), 1.39 (t, 3 H, $^3J_{HH}$ = 7.1 Hz, H_{11}), 1.07-1.03 (m, 2 H, H_{13a}), 0.84-0.81 (m, 2 H, H_{13b}).
^{13}C NMR (150 MHz, $CDCl_3$): δ 162.3 (C_9), 147.7 (C_8), 144.5 (C_6), 142.2 (C_5), 130.2 (q, $^2J_{CF}$ = 32.7 Hz, C_2), 126.3 (q, $^3J_{CF}$ = 3.7 Hz, C_3), 125.2 (C_4), 123.7 (q, $^1J_{CF}$ = 270.5 Hz, C_1), 106.2 (C_7), 61.2 (C_{10}), 14.4 (C_{11}), 9.1 (C_{13}), 7.7 (C_{12}).
^{19}F NMR (376 MHz, $CDCl_3$): δ -62.6 (s, CF_3).
FTIR (ν_{max}, cm^{-1}): 1732 (s, C=O ester stretch), 1617 (m), 1598 (w), 1554 (w), 1524 (w), 1482 (w), 1440 (m), 1421 (m), 1385 (m), 1325 (s), 1247 (s), 1234 (s), 1158 (s), 1128 (s), 1105 (s), 1064 (s), 1035 (m), 1025 (m), 1007 (s), 971 (w), 905 (w), 888 (w), 846 (s), 824 (s), 774 (s).
HRMS (ESI): calculated for $C_{16}H_{16}N_2O_2F_3$ $[M+H]^+$ 325.1164, found 325.1160; calculated for $C_{16}H_{15}N_2O_2F_3Na$ $[M+Na]^+$ 347.0983, found 347.0976.
R_f = 0.19 (10% EtOAc/hexane).
Anal. calcd. for $C_{16}H_{15}N_2O_2F_3$: C 59.26, H 4.66, N 8.64; found C 59.21, H 4.62, N 8.60.

5-cyclopropyl-1-(4-(trifluoromethyl)phenyl)-1*H*-pyrazole-3-carboxylic acid (15):

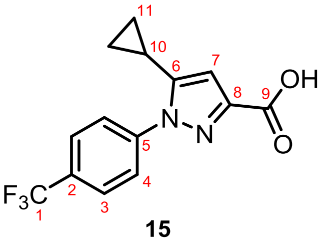

15

Isolated as a pale yellow amorphous solid (0.758 g, 2.6 mmol, 65%). The aqueous layer after extraction with CH_2Cl_2 from the synthesis of **16** was treated with aqueous HCl (3 M in H_2O) until pH 4, saturated with NaCl and extracted with CH_2Cl_2 (3 × 50 mL). The combined organic extracts were dried ($MgSO_4$), evaporated under reduced pressure and purified by silica gel column chromatography (eluent: 5% MeOH/1% AcOH/CH_2Cl_2); m.p. 134-136 °C.

^{1}H NMR (600 MHz, $CDCl_3$): δ 11.15 (br s, 1 H, COOH), 7.84 (d, $^{3}J_{HH}$ = 8.4 Hz, 2 H, H_3), 7.77 (d, $^{3}J_{HH}$ = 8.4 Hz, 2 H, H_4), 6.61 (s, 1 H, H_7), 1.84-1.79 (m, 1 H, H_{10}), 1.09-1.06 (m, 2 H, H_{11a}), 0.87-0.84 (m, 2 H, H_{11b}).
^{13}C NMR (150 MHz, $CDCl_3$): δ 166.5 (C_9), 148.3 (C_8), 143.4 (C_6), 142.0 (C_5), 130.4 (q, $^{2}J_{CF}$ = 32.8 Hz, C_2), 126.3 (q, $^{3}J_{CF}$ = 3.7 Hz, C_3), 125.1 (C_4), 123.7 (q, $^{1}J_{CF}$ = 270.6 Hz, C_1), 106.7 (C_7), 9.2 (C_{11}), 7.8 (C_{10}).
^{19}F NMR (376 MHz, $CDCl_3$): δ -62.6 (s, CF_3).
FTIR (ν_{max}, cm^{-1}): 3200-2500 (br w, COO-H stretch), 1697 (s, C=O carboxylic acid stretch), 1616 (m), 1523 (w), 1494 (m), 1477 (m), 1418 (m), 1382 (m), 1356 (w), 1324 (s), 1260 (m), 1235 (m), 1161 (s), 1123 (s), 1105 (s), 1067 (s), 1008 (s), 936 (m), 851 (s), 816 (m), 781 (m), 768 (m).
HRMS (ESI): calculated for $C_{14}H_{12}N_2O_2F_3$ $[M+H]^+$ 297.0851, found 297.0843; calculated for $C_{14}H_{11}N_2O_2F_3Na$ $[M+Na]^+$ 319.0670, found 319.0664.
R_f = 0.27 (5% MeOH/1% AcOH/CH_2Cl_2).
Anal. calcd. for $C_{14}H_{11}N_2O_2F_3$: C 56.76, H 3.74, N 9.46; found C 56.36, H 3.79, N 9.30.

Slow diffusion of hexane into a saturated solution of **15** in CH_2Cl_2 provided the CH_2Cl_2 solvate of **15** as colourless crystalline prisms for X-ray crystallographic analysis. The structure was unambiguously confirmed by single X-ray crystallography and deposited at the Cambridge Crystallographic Data Centre, deposition number CCDC 1430558.

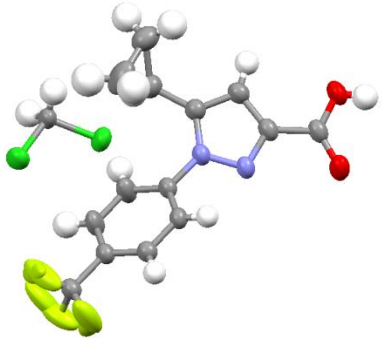

3-methyl-5-phenyl-1-(4-(trifluoromethyl)phenyl)-1*H*-pyrazole (18):

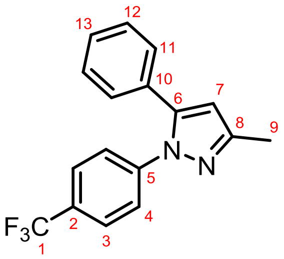

18

Isolated as a yellow oil (0.829 g, 2.7 mmol, 57%) after silica gel column chromatography (eluent: 5% EtOAc/hexane) following the general procedure for the telescoped synthesis of pyrazoles using 4-trifluoromethylaniline (0.773 g, 4.8 mmol) and 4-phenyl-3-butyn-2-one (0.761 g, 5.3 mmol). Data is consistent with a reported example.[8]

^{1}H NMR (600 MHz, $CDCl_3$): δ 7.56 (d, $^{3}J_{HH}$ = 8.5 Hz, 2 H, H_3), 7.40 (d, $^{3}J_{HH}$ = 8.5 Hz, 2 H, H_4), 7.34-7.31 (m, 3 H, H_{11} and H_{13}), 7.24-7.22 (m, 2 H, H_{12}), 6.33 (s, 1 H, H_7), 2.39 (s, 3 H, H_9).

^{13}C NMR (150 MHz, CDCl$_3$): δ 150.3 (C_8), 143.9 (C_6), 142.8 (C_5), 130.4 (C_{10}), 128.62 (C_{11}/C_{12}), 128.59 (C_{11}/C_{12}), 128.5 (q, $^2J_{CF}$ = 32.7 Hz, C_2), 128.4 (C_{13}), 125.9 (q, $^3J_{CF}$ = 3.7 Hz, C_3), 124.5 (C_4), 123.8 (q, $^1J_{CF}$ = 270.4 Hz, C_1), 108.9 (C_7), 13.5 (C_9).
^{19}F NMR (376 MHz, CDCl$_3$): δ -62.6 (s, CF_3).
FTIR (ν_{max}, cm^{-1}): 1616 (m), 1556 (w), 1525 (w), 1499 (w), 1446 (w), 1415 (m), 1379 (w), 1364 (m), 1321 (s), 1165 (s), 1121 (s), 1104 (s), 1074 (s), 1062 (s), 1017 (m), 968 (m), 917 (w), 843 (s), 802 (w), 785 (w), 761 (s).
HRMS (ESI): calculated for $C_{17}H_{14}N_2F_3$ $[M+H]^+$ 303.1109, found 303.1115.
R$_f$ = 0.21 (5% EtOAc/hexane).
Anal. calcd. for $C_{17}H_{13}N_2F_3$: C 67.54, H 4.33, N 9.27; found C 67.73, H 4.44, N 9.25.

5-(thiophen-2-yl)-1-(4-(trifluoromethyl)phenyl)-1*H*-pyrazole (22a) and **3-(thiophen-2-yl)-1-(4-(trifluoromethyl)phenyl)-1*H*-pyrazole (22b):**

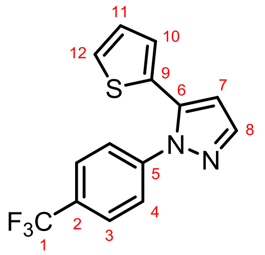

22a

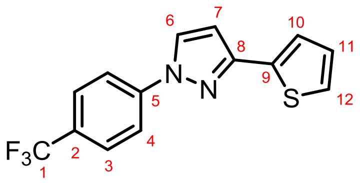

22b

Isolated as an inseparable mixture of regioisomers (72:28) as an orange amorphous solid (combined yield: 0.808 g, 2.7 mmol, 57%) after silica gel column chromatography (eluent: 10% EtOAc/hexane) following the general procedure for the telescoped synthesis of pyrazoles using 4-trifluoromethylaniline (0.773 g, 4.8 mmol) and (*E*)-3-(dimethylamino)-1-(thiophen-2-yl)prop-2-en-1-one (0.957 g, 5.3 mmol).

^{1}H NMR (600 MHz, CDCl$_3$): δ 7.98 (d, $^3J_{HH}$ = 2.6 Hz, 1 H, $H_{6,22b}$), 7.87 (d, $^3J_{HH}$ = 8.6 Hz, 2 H, $H_{4,22b}$), 7.73 (d, $^3J_{HH}$ = 1.7 Hz, 1 H, $H_{8,22a}$), 7.72 (d, $^3J_{HH}$ = 8.6 Hz, 2 H, $H_{3,22b}$), 7.65 (d, $^3J_{HH}$ = 8.4 Hz, 2 H, $H_{3,22a}$), 7.52 (d, $^3J_{HH}$ = 8.4 Hz, 2 H, $H_{4,22a}$), 7.44 (dd, $^3J_{HH}$ = 3.7 Hz, $^4J_{HH}$ = 0.9 Hz, 1 H, $H_{10,22b}$), 7.35 (dd, $^3J_{HH}$ = 5.0 Hz, $^4J_{HH}$ = 0.9 Hz, 1 H, $H_{12,22a}$), 7.32 (dd, $^3J_{HH}$ = 5.0 Hz, $^4J_{HH}$ = 0.9 Hz, 1 H, $H_{12,22b}$), 7.10 (dd, $^3J_{HH}$ = 5.0 Hz, 3.7 Hz, 1 H, $H_{11,22b}$), 7.00 (dd, $^3J_{HH}$ = 5.0 Hz, 3.7 Hz, 1 H, $H_{11,22a}$), 6.86 (dd, $^3J_{HH}$ = 3.7 Hz, 0.9 Hz, 1 H, $H_{10,22a}$), 6.72 (d, $^3J_{HH}$ = 2.6 Hz, 1 H, $H_{7,22b}$), 6.58 (d, $^3J_{HH}$ = 1.7 Hz, 1 H, $H_{7,22a}$).
^{13}C NMR (150 MHz, CDCl$_3$): δ 149.0 ($C_{8,22b}$), 142.6 ($C_{5,22a}$), 142.3 ($C_{5,22b}$), 141.0 ($C_{8,22a}$), 136.7 ($C_{6,22a}$), 135.8 ($C_{9,22b}$), 130.7 ($C_{9,22a}$), 129.8 (q, $^2J_{CF}$ = 32.6 Hz, $C_{2,22a}$), 128.1 (q, $^2J_{CF}$ = 32.6 Hz, $C_{2,22b}$), 128.0 ($C_{6,22b}$), 127.9 ($C_{10,22a}$), 127.6 ($C_{11,22a}$), 127.5 ($C_{11,22b}$), 127.1 ($C_{12,22a}$), 126.7 (q, $^3J_{CF}$ = 3.7 Hz, $C_{3,22b}$), 126.1 (q, $^3J_{CF}$ = 3.8 Hz, $C_{3,22a}$), 125.5 ($C_{4,22a}$), 125.4 ($C_{12,22b}$), 124.7 ($C_{10,22b}$), 124.0 (q, $^1J_{CF}$ = 270.2 Hz, $C_{1,22b}$), 123.8 (q, $^1J_{CF}$ = 270.5 Hz, $C_{1,22a}$), 118.6 ($C_{4,22b}$), 109.2 ($C_{7,22a}$), 106.0 ($C_{7,22b}$).
^{19}F NMR (376 MHz, CDCl$_3$): δ -62.2 (s, $CF_{3,22b}$), -62.5 (s, 3 F, $CF_{3,22a}$).
FTIR (ν_{max}, cm^{-1}): 1615 (m), 1559 (w), 1523 (m), 1505 (w), 1454 (w), 1425 (w), 1412 (m), 1379 (m), 1323 (s), 1209 (m), 1155 (m), 1101 (s), 1080 (m), 1067 (s), 1042 (m), 1013 (m), 945 (m), 921 (m), 911 (m), 882 (w), 845 (s), 833 (m), 826 (s), 791 (m), 753 (m).
HRMS (ESI): calculated for $C_{14}H_{10}N_2F_3S$ $[M+H]^+$ 295.0517, found 295.0517.
R$_f$ = 0.32 (10% EtOAc/hexane).
Anal. calcd. for $C_{14}H_9N_2F_3S$: C 57.14, H 3.08, N 9.52; found C 57.31, H 3.14, N 9.44.

3-methyl-1-(4-(trifluoromethyl)phenyl)-1*H*-pyrazol-5-amine (20):

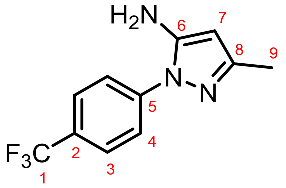

20

Isolated as yellow plate-like crystals (0.743 g, 3.1 mmol, 64%) after silica gel column chromatography (eluent: 30% EtOAc/hexane) following a modified version of the general procedure for the telescoped synthesis of pyrazoles using 4-trifluoromethylaniline (0.773 g, 4.8 mmol) and 3-aminocrotononitrile (0.433 g, 5.3 mmol). Vapourtec solvent and reagent line A were replaced with H_2O and a solution of sodium nitrite (0.2 M in H_2O) respectively; the other solvent/reagent lines were unchanged; m.p. 132-134 °C.

^{1}H NMR (400 MHz, $CDCl_3$): δ 7.72 (d, $^3J_{HH}$ = 8.7 Hz, 2 H, H_3), 7.67 (d, $^3J_{HH}$ = 8.7 Hz, 2 H, H_4), 5.45 (s, 1 H, H_7), 3.84 (br s, 2 H, NH_2), 2.20 (s, 3 H, H_9).
^{13}C NMR (100 MHz, $CDCl_3$): δ 150.4 (C_8), 145.5 (C_6), 141.8 (C_5), 128.3 (q, $^2J_{CF}$ = 32.7 Hz, C_2), 126.5 (q, $^3J_{CF}$ = 3.8 Hz, C_3), 123.9 (q, $^1J_{CF}$ = 270.7 Hz, C_1), 122.8 (C_4), 92.0 (C_7), 13.8 (C_9).
^{19}F NMR (376 MHz, $CDCl_3$): δ -62.4 (s, CF_3).
FTIR (ν_{max}, cm^{-1}): 3407 (w, N-H stretch), 3302 (w, N-H stretch), 3203 (w, C-H stretch), 1615 (m), 1592 (w), 1565 (m), 1527 (m), 1490 (w), 1465 (w), 1443 (w), 1417 (w), 1392 (w), 1373 (w), 1323 (m), 1163 (m), 1104 (s), 1067 (s), 1015 (m), 1008 (m), 843 (m), 805 (w), 771 (w).
HRMS (ESI): calculated for $C_{11}H_{11}N_3F_3$ $[M+H]^+$ 242.0905, found 242.0914.
R_f = 0.33 (30% EtOAc/hexane).
Anal. calcd. for $C_{11}H_{10}N_3F_3$: C 54.77, H 4.18, N 17.42; found C 54.76, H 4.16, N 17.11.

Slow diffusion of hexane into a saturated solution of **20** in CH_2Cl_2 provided **20** as colourless crystalline plates for X-ray crystallographic analysis. The structure was unambiguously confirmed by single X-ray crystallography and deposited at the Cambridge Crystallographic Data Centre, deposition number CCDC 1430559.

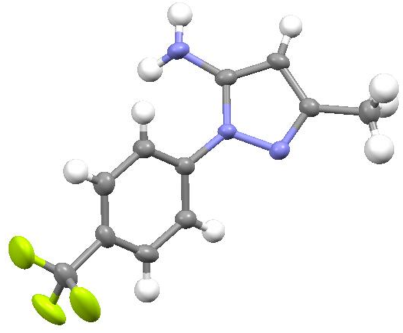

3,5-dimethyl-1-(4-nitrophenyl)-1*H*-pyrazole (6):

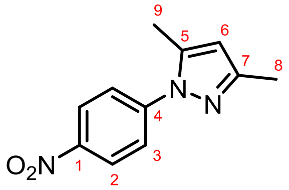

6

Isolated as a yellow amorphous solid (0.797 g, 3.7 mmol, 76%) after silica gel column chromatography (eluent: 20% EtOAc/hexane) following the general procedure for the telescoped synthesis of pyrazoles using 4-nitroaniline (0.663 g, 4.8 mmol) and pentane-2,4-dione (0.529 g, 5.3 mmol); m.p. 101-102 °C (lit. m.p.[9] 101-103 °C). Data is consistent with a reported example.[9]

^{1}H NMR (400 MHz, $CDCl_3$): δ 8.31 (d, $^3J_{HH}$ = 9.1 Hz, 2 H, H_2), 7.67 (d, $^3J_{HH}$ = 9.1 Hz, 2 H, H_3), 6.07 (s, 1 H, H_6), 2.42 (s, 3 H, H_8), 2.30 (s, 3 H, H_9).
^{13}C NMR (100 MHz, $CDCl_3$): δ 150.8 (C_7), 145.6 (C_1), 145.0 (C_4), 139.8 (C_5), 124.7 (C_2), 123.5 (C_3), 109.3 (C_6), 13.5 (C_8), 13.1 (C_9).
FTIR (ν_{max}, cm^{-1}): 1608 (w), 1594 (m), 1570 (w), 1516 (s, NO_2 stretch), 1503 (m), 1415 (w), 1380 (w), 1359 (w), 1330 (s, NO_2 stretch), 1177 (w), 1124 (w), 1109 (m), 1034 (m), 971 (m), 853 (s), 800 (m), 769 (m).
HRMS (ESI): calculated for $C_{11}H_{12}N_3O_2$ $[M+H]^+$ 218.0930, found 218.0940.
R_f = 0.31 (20% EtOAc/hexane).
Anal. calcd. for $C_{11}H_{11}N_3O_2$: C 60.82, H 5.10, N 19.34; found C 60.85, H 5.13, N 19.05.

1-(3-bromophenyl)-3,5-dimethyl-1*H*-pyrazole (8):

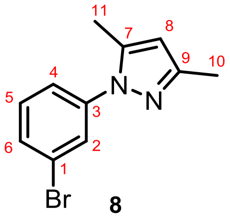

8

Isolated as a yellow oil (0.836 g, 3.3 mmol, 69%) after silica gel column chromatography (eluent: 10% EtOAc/hexane) following the general procedure for the telescoped synthesis of pyrazoles using 3-bromoaniline (0.826 g, 4.8 mmol) and pentane-2,4-dione (0.529 g, 5.3 mmol).

^{1}H NMR (400 MHz, $CDCl_3$): δ 7.63 (s, 1 H, H_2), 7.44 (d, $^3J_{HH}$ = 7.9 Hz, 1 H, H_6), 7.36 (d, $^3J_{HH}$ = 7.9 Hz, 1 H, H_4), 7.28 (t, $^3J_{HH}$ = 7.9 Hz, 1 H, H_5), 5.98 (s, 1 H, H_8), 2.30 (s, 3 H, H_{10}), 2.28 (s, 3 H, H_{11}).
^{13}C NMR (100 MHz, $CDCl_3$): δ 149.5 (C_9), 141.1 (C_3), 139.5 (C_7), 130.2 (C_5), 130.1 (C_6), 127.7 (C_2), 122.9 (C_4), 122.5 (C_1), 107.6 (C_8), 13.5 (C_{10}), 12.5 (C_{11}).
FTIR (ν_{max}, cm^{-1}): 1591 (s), 1578 (s), 1557 (m), 1488 (s), 1426 (m), 1378 (m), 1360 (m), 1247 (w), 1132 (w), 1092 (w), 1068 (w), 1041 (w), 1020 (w), 998 (w), 974 (w), 874 (w), 776 (s).
HRMS (ESI): calculated for $C_{11}H_{12}N_{12}Br$ $[M+H]^+$ 251.0184, found 251.0196.
R_f = 0.31 (10% EtOAc/hexane).
Anal. calcd. for $C_{11}H_{11}N_{12}Br$: C 52.61, H 4.41, N 11.15, Br 31.82; found C 52.58, H 4.44, N 11.07, Br 31.93.

1-(4-fluorophenyl)-3,5-dimethyl-1*H*-pyrazole (10):

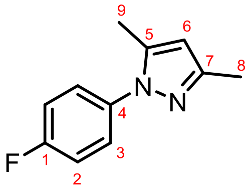

10

Isolated as a yellow oil (0.502 g, 2.6 mmol, 55%) after silica gel column chromatography (eluent: 10% EtOAc/hexane) following the general procedure for the telescoped synthesis of pyrazoles using 4-fluoroaniline (0.533 g, 4.8 mmol) and pentane-2,4-dione (0.529 g, 5.3 mmol). Data is consistent with a reported example.[10]

^{1}H NMR (400 MHz, CDCl$_3$): δ 7.38 (dd, $^3J_{HH}$ = 8.8 Hz, $^4J_{HF}$ = 4.8 Hz, 2 H, H$_3$), 7.12 (t, $^3J_{HH}$ = $^3J_{HF}$ = 8.8 Hz, 2 H, H$_2$), 5.98 (s, 1 H, H$_6$), 2.28 (s, 3 H, H$_8$), 2.26 (s, 3 H, H$_9$).
^{13}C NMR (100 MHz, CDCl$_3$): δ 161.6 (d, $^1J_{CF}$ = 245.8 Hz, C$_1$), 149.0 (C$_7$), 139.4 (C$_5$), 136.1 (d, $^4J_{CF}$ = 3.1 Hz, C$_4$), 126.6 (d, $^3J_{CF}$ = 8.5 Hz, C$_3$), 115.8 (d, $^2J_{CF}$ = 22.7 Hz, C$_2$), 106.8 (C$_6$), 13.4 (C$_8$), 12.2 (C$_9$).
^{19}F NMR (376 MHz, CDCl$_3$): δ -114.5 (s, 1 F, F$_1$).
FTIR (ν_{max}, cm^{-1}): 1557 (m), 1512 (s), 1416 (m), 1384 (m), 1366 (m), 1220 (m), 1154 (m), 1131 (w), 1094 (w), 1036 (m), 1017 (w), 977 (w), 838 (s), 821 (m), 784 (m), 760 (w).
HRMS (ESI): calculated for $C_{11}H_{12}N_2F$ [M+H]$^+$ 191.0985, found 191.0994.
R$_f$ = 0.27 (10% EtOAc/hexane).
Anal. calcd. for $C_{11}H_{11}N_2F$: C 69.46, H 5.83, N 14.73; found C 69.38, H 5.94, N 14.68.

3,5-dimethyl-1-(4-(pentafluorosulfanyl)phenyl)-1*H*-pyrazole (12):

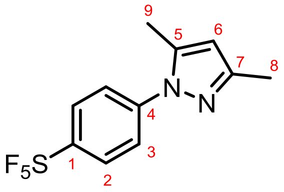

12

Isolated as a slow-to-crystallise off-white amorphous solid (0.644 g, 2.2 mmol, 45%) after silica gel column chromatography (eluent: 10% EtOAc/hexane) following the general procedure for the telescoped synthesis of pyrazoles using 4-aminophenylsulfur pentafluoride (1.052 g, 4.8 mmol) and pentane-2,4-dione (0.529 g, 5.3 mmol); m.p. 57-58 °C.

^{1}H NMR (500 MHz, CDCl$_3$): δ 7.84-7.82 (m, 2 H, H$_2$), 7.57 (d, $^3J_{HH}$ = 9.0 Hz, 2 H, H$_3$), 6.04 (s, 1 H, H$_6$), 2.38 (s, 3 H, H$_8$), 2.29 (s, 3 H, H$_9$).
^{13}C NMR (125 MHz, CDCl$_3$): δ 151.6 (qn, $^2J_{CF}$ = 17.9 Hz, C$_1$), 150.3 (C$_7$), 142.3 (C$_4$), 139.6 (C$_5$), 127.0 (qn, $^3J_{CF}$ = 4.3 Hz, C$_2$), 123.6 (C$_3$), 108.5 (C$_6$), 13.5 (C$_8$), 12.8 (C$_9$).
^{19}F NMR (376 MHz, CDCl$_3$): δ 83.1 (qn, $^2J_{FF}$ = 150.1 Hz, SF$_{ax}$), 62.3 (d, $^2J_{FF}$ = 150.1 Hz, SF$_{eq}$).
FTIR (ν_{max}, cm^{-1}): 1603 (m), 1561 (m), 1507 (m), 1447 (w), 1416 (m), 1383 (m), 1364 (m), 1193 (w), 1134 (w), 1101 (m), 1037 (w), 975 (w), 815 (s, S-F stretch), 767 (s).
HRMS (ESI): calculated for $C_{11}H_{12}N_2F_5S$ [M+H]$^+$ 299.0641, found 299.0656.
R$_f$ = 0.24 (10% EtOAc/hexane).
Anal. calcd. for $C_{11}H_{11}N_2F_5S$: C 44.29, H 3.72, N 9.39; found C 44.48, H 3.76, N 9.27.

2-(3,5-dimethyl-1*H*-pyrazol-1-yl)benzoic acid (7):

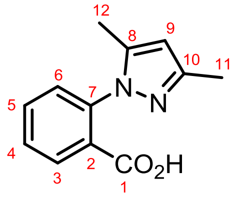

7

Isolated as a yellow amorphous solid (0.631 g, 2.9 mmol, 61%) following a modified version of the general procedure for the telescoped synthesis of pyrazoles using anthranilic acid (0.658 g, 4.8 mmol) and pentane-2,4-dione (0.529 g, 5.3 mmol). Vapourtec solvent and reagent line A were replaced with H_2O and a solution of sodium nitrite (0.2 M in H_2O) respectively; the other solvent/reagent lines were unchanged. The output was treated with an aqueous solution of sodium hydroxide (3 M in H_2O) until pH 10 and the aqueous phase washed with diethyl ether (2 × 25 mL). The mixture was carefully reacidified with aqueous HCl (3 M in H_2O) until pH 4, saturated with NaCl and extracted with EtOAc (3 × 50 mL). The combined organic extracts were dried ($MgSO_4$), evaporated under reduced pressure and purified by silica gel column chromatography (eluent: 5% MeOH/1% AcOH/CH_2Cl_2); m.p. 149-152 °C, (lit. m.p.[11] 151-153 °C). Data is consistent with a reported example.[11]

^{1}H NMR (400 MHz, $CDCl_3$): δ 10.42 (br s, 1 H, COOH), 8.03 (d, $^3J_{HH}$ = 7.6 Hz, 1 H, H_3), 7.58 (t, $^3J_{HH}$ = 7.6 Hz, 1 H, H_5), 7.49 (t, $^3J_{HH}$ = 7.6 Hz, 1 H, H_4), 7.26 (d, $^3J_{HH}$ = 7.6 Hz, 1 H, H_6), 6.01 (s, 1 H, H_9), 2.28 (s, 3 H, H_{11}), 2.15 (s, 3 H, H_{12}).
^{13}C NMR (100 MHz, $CDCl_3$): δ 167.7 (C_1), 149.8 (C_{10}), 142.0 (C_7), 137.3 (C_8), 132.4 (C_3), 132.3 (C_5), 129.7 (C_2), 128.8 (C_4), 128.2 (C_6), 107.0 (C_9), 13.2 (C_{11}), 11.8 (C_{12}).
FTIR (ν_{max}, cm^{-1}): 3200-2400 (br w, COO-H stretch), 1704 (m, C=O carboxylic acid stretch), 1598 (w), 1579 (w), 1555 (w), 1500 (m), 1423 (w), 1385 (w), 1247 (s), 1165 (m), 1140 (m), 1089 (m), 1041 (m), 1013 (m), 984 (w), 960 (w), 805 (m), 780 (s), 756 (s).
HRMS (ESI): calculated for $C_{12}H_{13}N_2O_2$ $[M+H]^+$ 217.0977, found 217.0980; calculated for $C_{12}H_{12}N_2O_2Na$ $[M+Na]^+$ 239.0796, found 239.0798.
***R**$_f$* (5% MeOH/1% AcOH/CH_2Cl_2) = 0.35.

2-(4-(3,5-dimethyl-1*H*-pyrazol-1-yl)phenyl)ethanol (9):

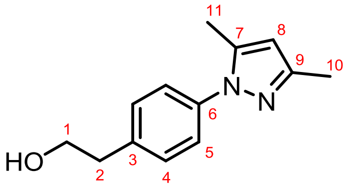

9

Isolated as a yellow amorphous solid (0.589 g, 2.7 mmol, 57%) after silica gel column chromatography (eluent: 70% EtOAc/hexane) following a modified version of the general procedure for the telescoped synthesis of pyrazoles using 4-aminophenethyl alcohol (0.692 g, 4.8 mmol) and pentane-2,4-dione (0.529 g, 5.3 mmol). Vapourtec solvent and reagent line A were replaced with H_2O and a solution of sodium nitrite (0.2 M in H_2O) respectively; the other solvent/reagent lines were unchanged. The 5 mL reaction coil at r.t. was replaced with a 15 mL reaction coil held at 30 °C in a water bath; m.p. 97-98 °C. Data is consistent with a reported example.[12]

^{1}H NMR (400 MHz, CDCl$_3$): δ 7.34 (d, $^3J_{HH}$ = 8.4 Hz, 2 H, H$_4$), 7.27 (d, $^3J_{HH}$ = 8.4 Hz, 2 H, H$_5$), 5.98 (s, 1 H, H$_8$), 3.80 (t, $^3J_{HH}$ = 6.7 Hz, 2 H, H$_1$), 2.87 (t, $^3J_{HH}$ = 6.7 Hz, 2 H, H$_2$), 2.28 (two superimposed s, 3 H each, H$_{10}$ and H$_{11}$), 2.12 (br s, 1 H, OH).
^{13}C NMR (100 MHz, CDCl$_3$): δ 148.9 (C$_9$), 139.4 (C$_7$), 138.3 (C$_6$), 138.0 (C$_3$), 129.5 (C$_4$), 124.9 (C$_5$), 106.7 (C$_8$), 63.3 (C$_1$), 38.9 (C$_2$), 13.4 (C$_{10}$), 12.3 (C$_{11}$).
FTIR (ν_{max}, cm^{-1}): 3310 (br w, O-H stretch), 2926 (w, C-H stretch), 2855 (w, C-H stretch), 1550 (m), 1519 (s), 1418 (m), 1381 (m), 1367 (m), 1167 (w), 1139 (w), 1112 (w), 1055 (s), 1025 (m), 986 (w), 858 (s), 835 (w), 818 (w), 787 (s), 771 (w).
HRMS (ESI): calculated for $C_{13}H_{17}N_2O$ [M+H]$^+$ 217.1341, found 217.1345.
R$_f$ = 0.30 (70% EtOAc/hexane).
Anal. calcd. for $C_{13}H_{16}N_2O$: C 72.19, H 7.46, N 12.95; found C 71.83, H 7.37, N 12.66.

2-chloro-3-(3,5-dimethyl-1*H*-pyrazol-1-yl)pyridine (11):

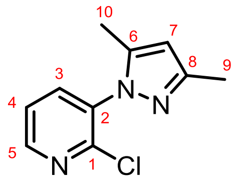

11

Isolated as a yellow oil (0.510 g, 2.5 mmol, 51%) after silica gel column chromatography (eluent: 50% EtOAc/hexane) following a modified version of the general procedure for the telescoped synthesis of pyrazoles using 3-amino-2-chloropyridine (0.617 g, 4.8 mmol) and pentane-2,4-dione (0.529 g, 5.3 mmol). Vapourtec solvent and reagent line A were replaced with H_2O and a solution of sodium nitrite (0.2 M in H_2O) respectively; the other solvent/reagent lines were unchanged.

^{1}H NMR (400 MHz, CDCl$_3$): δ 8.46 (dd, $^3J_{HH}$ = 4.8 Hz, $^4J_{HH}$ = 1.8 Hz, 1 H, H$_5$), 7.74 (dd, $^3J_{HH}$ = 7.8 Hz, $^4J_{HH}$ = 1.8 Hz, 1 H, H$_3$), 7.36 (dd, $^3J_{HH}$ = 7.8, 4.8 Hz, 1 H, H$_4$), 6.00 (s, 1 H, H$_7$), 2.27 (s, 3 H, H$_9$), 2.12 (s, 3 H, H$_{10}$).
^{13}C NMR (100 MHz, CDCl$_3$): δ 150.4 (C$_8$), 149.7 (C$_5$), 149.5 (C$_1$), 141.5 (C$_2$), 138.5 (C$_3$), 134.5 (C$_6$), 122.9 (C$_4$), 106.3 (C$_7$), 13.5 (C$_9$), 11.3 (C$_{10}$).
FTIR (ν_{max}, cm^{-1}): 1716 (m), 1634 (w), 1558 (m), 1484 (m), 1408 (s), 1377 (m), 1364 (m), 1217 (m), 1125 (m), 1103 (m), 1058 (m), 1027 (m), 1008 (m), 972 (w), 899 (w), 812 (s), 784 (s).
HRMS (ESI): calculated for $C_{10}H_{11}N_3Cl$ [M+H]$^+$ 208.0636, found 208.0626.
R$_f$ = 0.31 (50% EtOAc/hexane).

3-(3,5-dimethyl-1*H*-pyrazol-1-yl)quinoline (13):

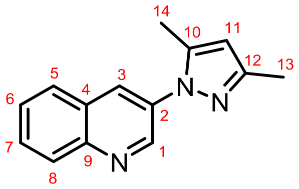

13

Isolated as a yellow-brown amorphous solid (0.595 g, 2.7 mmol, 56%) after silica gel column chromatography (eluent: 30% EtOAc/hexane) following a modified version of the general procedure for the telescoped synthesis of pyrazoles using 3-aminoquinoline (0.692 g, 4.8 mmol) and pentane-2,4-dione (0.529 g, 5.3 mmol). Vapourtec solvent and reagent line A were replaced with H_2O and a solution of sodium nitrite (0.2 M in H_2O) respectively; the

other solvent/reagent lines were unchanged; m.p. 71-73 °C. Data is consistent with a reported example.[13]

^{1}H NMR (400 MHz, CDCl$_3$): δ 9.07 (d, $^4J_{HH}$ = 2.4 Hz, 1 H, H$_1$), 8.19 (d, $^4J_{HH}$ = 2.4 Hz, 1 H, H$_3$), 8.15 (d, $^3J_{HH}$ = 8.5 Hz, 1 H, H$_8$), 7.86 (d, $^3J_{HH}$ = 8.1 Hz, 1 H, H$_5$), 7.76-7.72 (m, 1 H, H$_7$), 7.61-7.58 (m, 1 H, H$_6$), 6.08 (s, 1 H, H$_{11}$), 2.39 (s, 3 H, H$_{13}$), 2.33 (s, 3 H, H$_{14}$).
^{13}C NMR (100 MHz, CDCl$_3$): δ 150.2 (C$_{12}$), 147.1 (C$_1$), 146.7 (C$_9$), 140.0 (C$_{10}$), 133.4 (C$_2$), 129.7 (C$_7$), 129.6 (C$_3$), 129.4 (C$_8$), 127.9 (C$_5$), 127.6 (C$_4$), 127.5 (C$_6$), 107.9 (C$_{11}$), 13.5 (C$_{13}$), 12.4 (C$_{14}$).
FTIR (ν_{max}, cm^{-1}): 1605 (w), 1563 (w), 1495 (w), 1482 (w), 1423 (m), 1385 (m), 1361 (m), 1121 (w), 1036 (w), 990 (w), 951 (m), 905 (m), 867 (w), 826 (m), 786 (m), 756 (s).
HRMS (ESI): calculated for $C_{14}H_{14}N_3$ [M+H]$^+$ 224.1188, found 224.1194.
R_f (30% EtOAc/hexane) = 0.22.
Anal. calcd. for $C_{14}H_{13}N_3$: C 75.31, H 5.87, N 18.82; found C 74.91, H 5.85, N 18.40.

5-(furan-2-yl)-1-(4-(pentafluorosulfanyl)phenyl)-3-(trifluoromethyl)-1*H*-pyrazole (23):

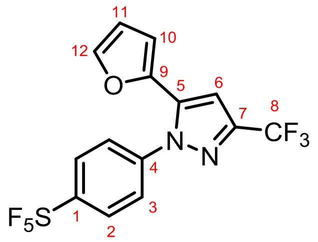

23

Isolated as a viscous yellow oil (0.893 g, 2.2 mmol, 46%) after silica gel column chromatography (eluent: 5% EtOAc/hexane) following the general procedure for the telescoped synthesis of pyrazoles using 4-aminophenylsulfur pentafluoride (1.052 g, 4.8 mmol) and 4,4,4-trifluoro-1-(2-furyl)-1,3-butanedione (1.088 g, 5.3 mmol).
^{1}H NMR (500 MHz, CDCl$_3$): δ 7.88-7.84 (m, 2 H, H$_2$), 7.55 (d, $^3J_{HH}$ = 8.9 Hz, 2 H, H$_3$), 7.46 (dd, $^3J_{HH}$ = 1.8 Hz, $^4J_{HH}$ = 0.6 Hz, 1 H, H$_{12}$), 6.91 (s, 1 H, H$_6$), 6.46 (dd, $^3J_{HH}$ = 3.5 Hz, 1.8 Hz, H$_{11}$), 6.32 (dd, $^3J_{HH}$ = 3.5 Hz, $^4J_{HH}$ = 0.6 Hz, 1 H, H$_{10}$).
^{13}C NMR (125 MHz, CDCl$_3$): δ 153.5 (qn, $^2J_{CF}$ = 18.3 Hz, C$_1$), 144.2 (q, $^2J_{CF}$ = 38.6 Hz, C$_7$), 143.9 (C$_{12}$), 142.1 (C$_9$), 141.6 (C$_4$), 135.8 (C$_5$), 127.1 (qn, $^3J_{CF}$ = 4.5 Hz, C$_2$), 125.4 (C$_3$), 120.8 (q, $^1J_{CF}$ = 267.6 Hz, C$_8$), 111.7 (C$_{11}$), 111.0 (C$_{10}$), 105.5 (C$_6$).
^{19}F NMR (376 MHz, CDCl$_3$): δ 82.1 (qn, $^2J_{FF}$ = 150.2 Hz, SF$_{ax}$), 62.0 (d, $^2J_{FF}$ = 150.2 Hz, SF$_{eq}$), -63.6 (s, CF$_3$).
FTIR (ν_{max}, cm^{-1}): 1604 (w), 1499 (m), 1472 (w), 1433 (w), 1414 (w), 1386 (w), 1368 (w), 1279 (w), 1244 (m), 1218 (w), 1171 (w), 1135 (m), 1104 (m), 1079 (w), 1015 (w), 991 (w), 974 (m), 897 (w), 825 (s, S-F stretch).
HRMS (ESI): calculated for $C_{14}H_9N_2OF_8S$ [M+H]$^+$ 405.0308, found 405.0318.
R_f = 0.27 (5% EtOAc/hexane).
Anal. calcd. for $C_{14}H_8N_2OF_8S$: C 41.59, H 1.99, N 6.93; found C 41.49, H 1.99, N 6.89.

2-chloro-3-(5-phenyl-3-(trifluoromethyl)-1*H*-pyrazol-1-yl)pyridine (24):

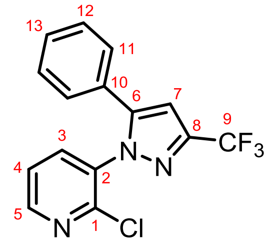

24

Isolated as a viscous orange oil (0.739 g, 2.3 mmol, 48%) after silica gel column chromatography (eluent: 20% EtOAc/hexane) following the general procedure for the telescoped synthesis of pyrazoles using 3-amino-2-chloropyridine (0.617 g, 4.8 mmol) and 4,4,4-trifluoro-1-phenyl-1,3-butanedione (1.141 g, 5.3 mmol).

^{1}H NMR (600 MHz, $CDCl_3$): δ 8.50 (dd, $^3J_{HH}$ = 4.8 Hz, $^4J_{HH}$ = 1.7 Hz, 1 H, H_5), 7.82 (dd, $^3J_{HH}$ = 7.8 Hz, $^4J_{HH}$ = 1.7 Hz, 1 H, H_3), 7.38 (dd, $^3J_{HH}$ = 7.8 Hz, 4.8 Hz, 1 H, H_4), 7.36-7.33 (m, 1 H, H_{13}), 7.32-7.29 (m, 2 H, H_{12}), 7.20-7.18 (m, 2 H, H_{11}), 6.82 (s, 1 H, H_7).
^{13}C NMR (150 MHz, $CDCl_3$): δ 150.5 (C_5), 149.2 (C_1), 147.0 (C_2), 144.5 (q, $^2J_{CF}$ = 38.4 Hz, C_8), 138.3 (C_3), 134.2 (C_6), 129.5 (C_{13}), 128.9 (C_{11}), 128.2 (C_{10}), 128.1 (C_{12}), 122.9 (C_4), 121.0 (q, $^1J_{CF}$ = 267.5 Hz, C_9), 104.8 (q, $^3J_{CF}$ = 1.9 Hz, C_7).
^{19}F NMR (376 MHz, $CDCl_3$): δ -62.4 (s, CF_3).
FTIR (ν_{max}, cm^{-1}): 1628 (w), 1570 (w), 1471 (s), 1444 (w), 1415 (s), 1373 (w), 1280 (m), 1238 (s), 1201 (m), 1159 (s), 1120 (s), 1094 (s), 1074 (m), 1062 (m), 1027 (w), 1001 (w), 975 (s), 917 (w), 810 (s), 761 (s).
HRMS (ESI): calculated for $C_{15}H_{10}N_3F_3Cl$ $[M+H]^+$ 324.0515, found 324.0518.
R_f = 0.20 (20% EtOAc/hexane).

5-(furan-2-yl)-1-(4-nitrophenyl)-3-(trifluoromethyl)-1*H*-pyrazole (25):

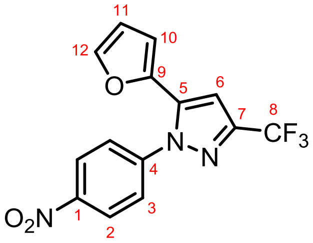

25

Isolated as an orange amorphous solid (3.74 g, 11.6 mmol, 40%) after silica gel column chromatography (eluent: 10% EtOAc/hexane) following a modified version of the general procedure for the telescoped synthesis of pyrazoles using 4-nitroaniline (3.98 g, 28.8 mmol) and 4,4,4-trifluoro-1-(2-furyl)-1,3-butanedione (6.53 g, 31.7 mmol). The flow set-up was run for 12 h at steady state and the output was treated with an aqueous solution of saturated $NaHCO_3$ (150 mL), extracted with diethyl ether (3 × 300 mL). The combined organic extracts were washed with brine (300 mL), dried ($MgSO_4$) and evaporated under reduced pressure; m.p. 100-101 °C. Data is consistent with a reported example.[14]

^{1}H NMR (600 MHz, $CDCl_3$): δ 8.32 (d, $^3J_{HH}$ = 9.0 Hz, 2 H, H_2), 7.62 (d, $^3J_{HH}$ = 9.0 Hz, 2 H, H_3), 7.45 (d, $^3J_{HH}$ = 1.8 Hz, 1 H, H_{12}), 6.91 (s, 1 H, H_6), 6.47 (dd, $^3J_{HH}$ = 3.4 Hz, 1.8 Hz, 1 H, H_{11}), 6.39 (d, $^3J_{HH}$ = 3.4 Hz, 1 H, H_{10}).

^{13}C NMR (150 MHz, $CDCl_3$): δ 147.4 (C_1), 144.5 (q, $^2J_{CF}$ = 38.7 Hz, C_7), 144.1 (C_4), 144.0 (C_{12}), 141.9 (C_9), 135.9 (C_5), 125.7 (C_2), 124.6 (C_3), 120.7 (q, $^1J_{CF}$ = 267.6 Hz, C_8), 111.8 (C_{11}), 111.4 (C_{10}), 106.0 (q, $^3J_{CF}$ = 1.9 Hz, C_6).
^{19}F NMR (376 MHz, $CDCl_3$): δ -62.6 (s, CF_3).
FTIR (ν_{max}, cm^{-1}): 1597 (m), 1507 (m, NO_2 stretch), 1496 (m), 1474 (m), 1433 (w), 1421 (w), 1384 (w), 1355 (m, NO_2 stretch), 1274 (m), 1245 (s), 1224 (m), 1167 (m), 1153 (m), 1120 (s), 1099 (s), 1067 (m), 1025 (m), 986 (w), 970 (s), 897 (m), 884 (w), 856 (s), 814 (s).
HRMS (ESI): calculated for $C_{14}H_9N_3O_3F_3$ $[M+H]^+$ 324.0596, found 324.0594.
R_f = 0.29 (10% EtOAc/hexane).
Anal. calcd. for $C_{14}H_8N_3O_3F_3$: C 52.02, H 2.49, N 13.00; found C 51.93, H 2.48, N 12.63.

3. NMR spectra

4-(5-(*p*-tolyl)-3-(trifluoromethyl)-1*H*-pyrazol-1-yl)benzenesulfonamide, celecoxib (1):

^{1}H NMR, 600 MHz, d_3-MeCN:

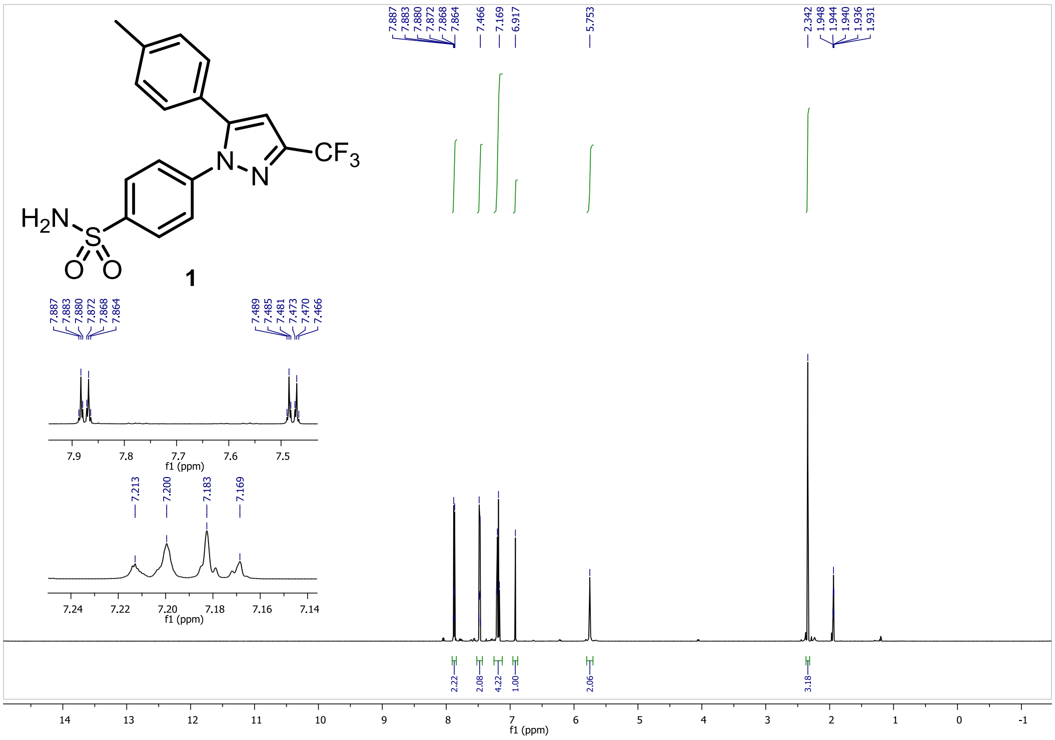

^{13}C NMR, 150 MHz, d_3-MeCN:

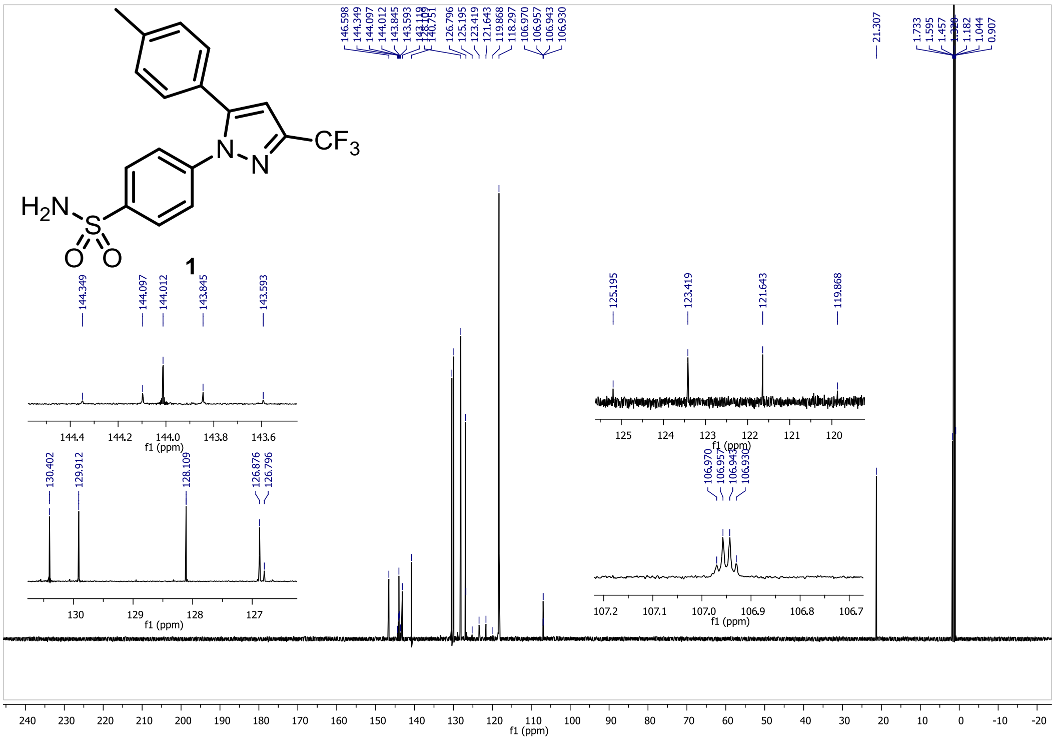

^{19}F NMR, 376 MHz, d_3-MeCN:

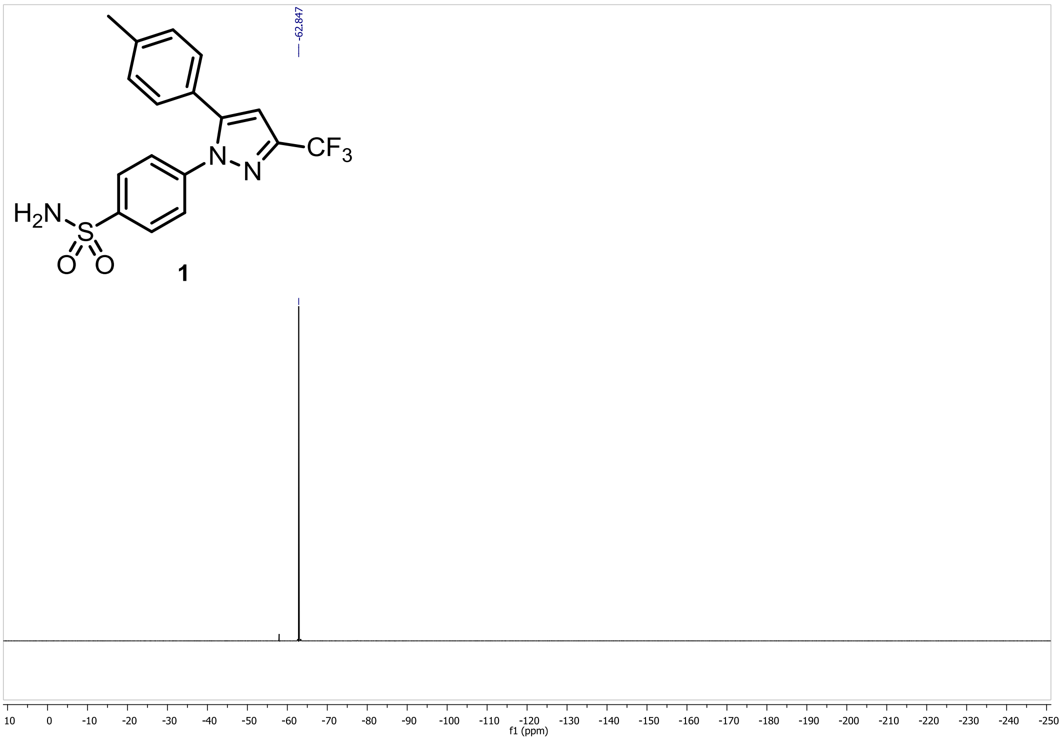

3,5-dimethyl-1-(4-(trifluoromethyl)phenyl)-1*H*-pyrazole (5):

^{1}H NMR, 400 MHz, $CDCl_3$:

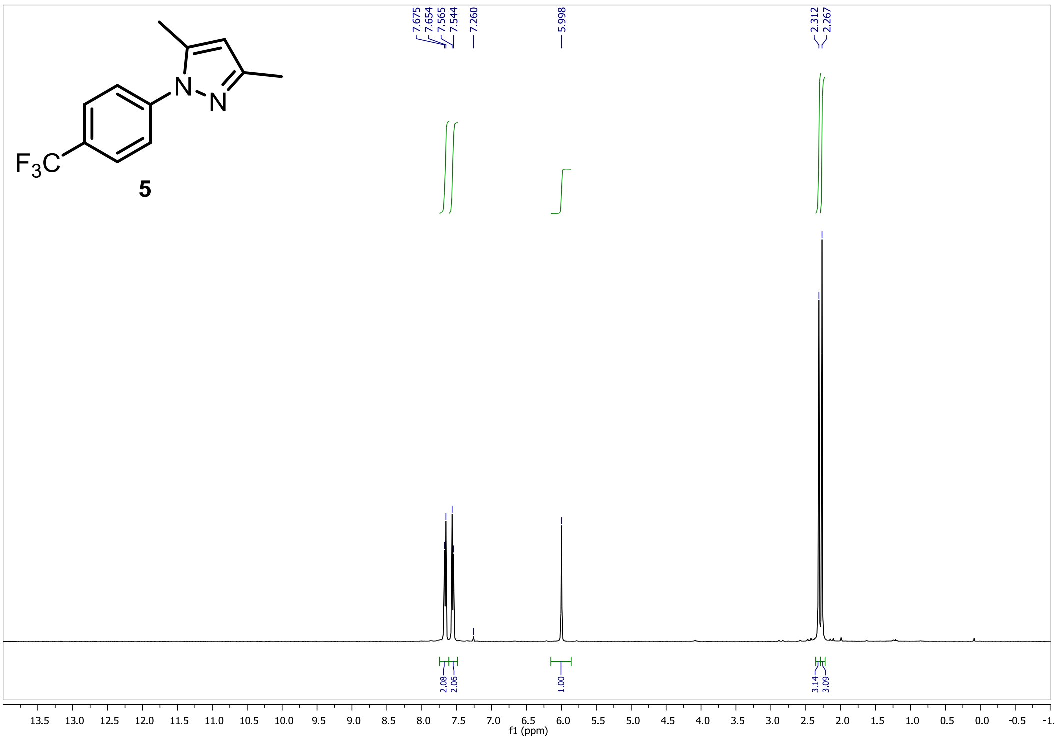

^{13}C NMR, 100 MHz, $CDCl_3$:

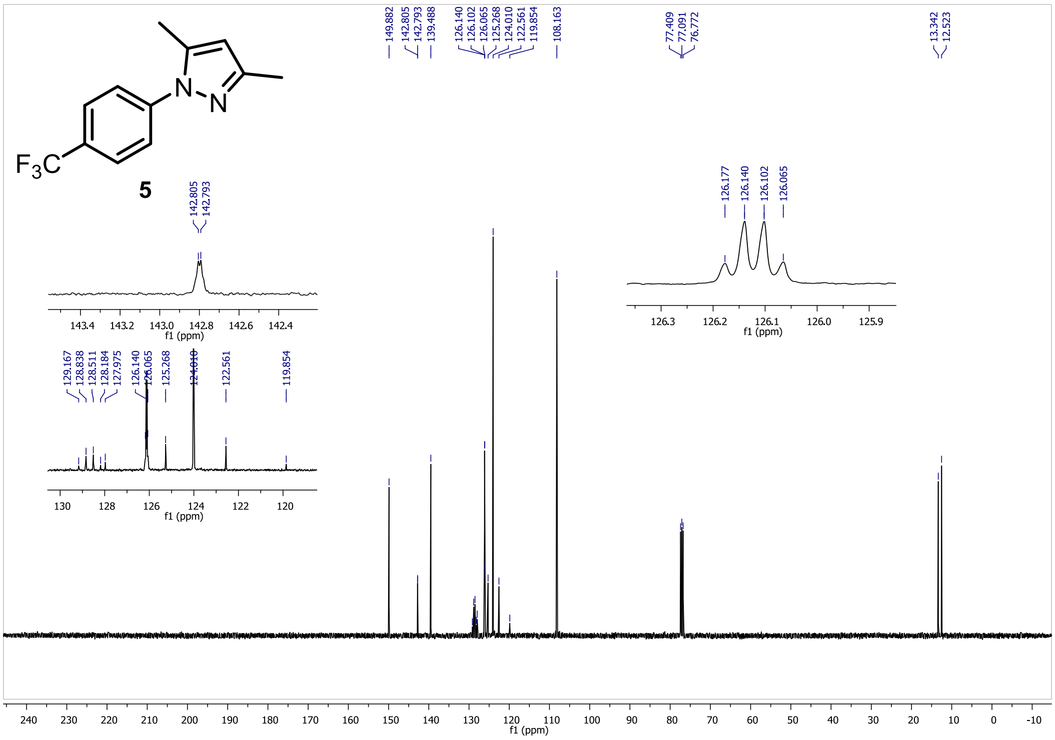

^{19}F NMR, 376 MHz, $CDCl_3$:

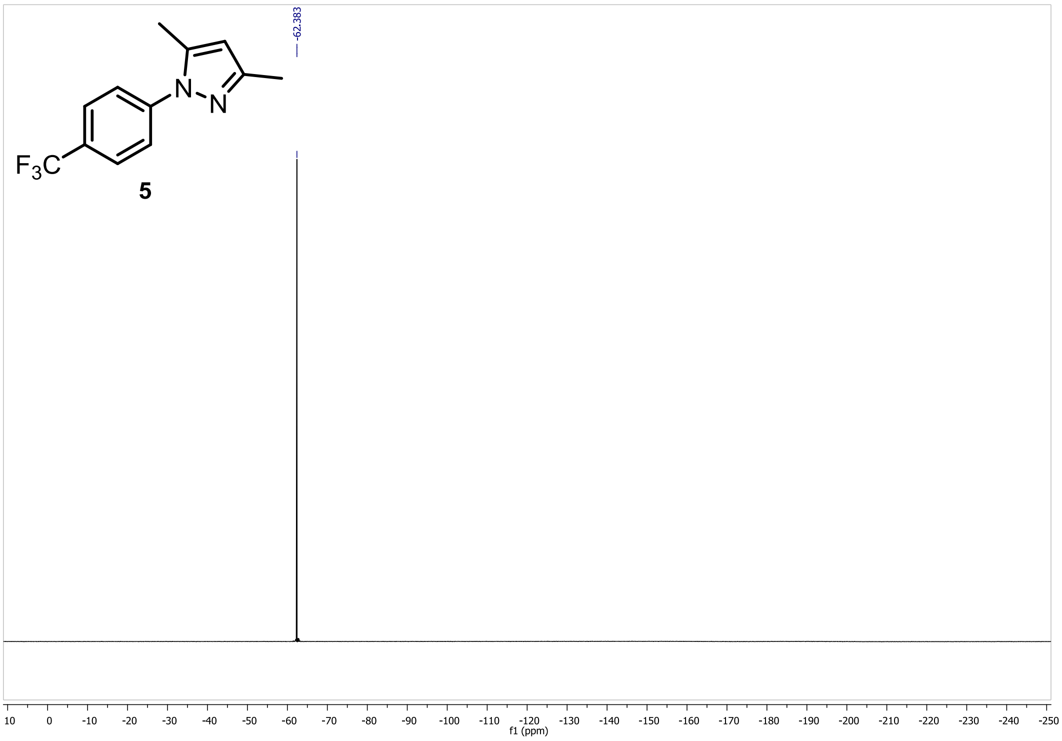

3,5-dimethyl-1-(4-nitrophenyl)-1*H*-pyrazole (6):

^{1}H NMR, 400 MHz, $CDCl_3$:

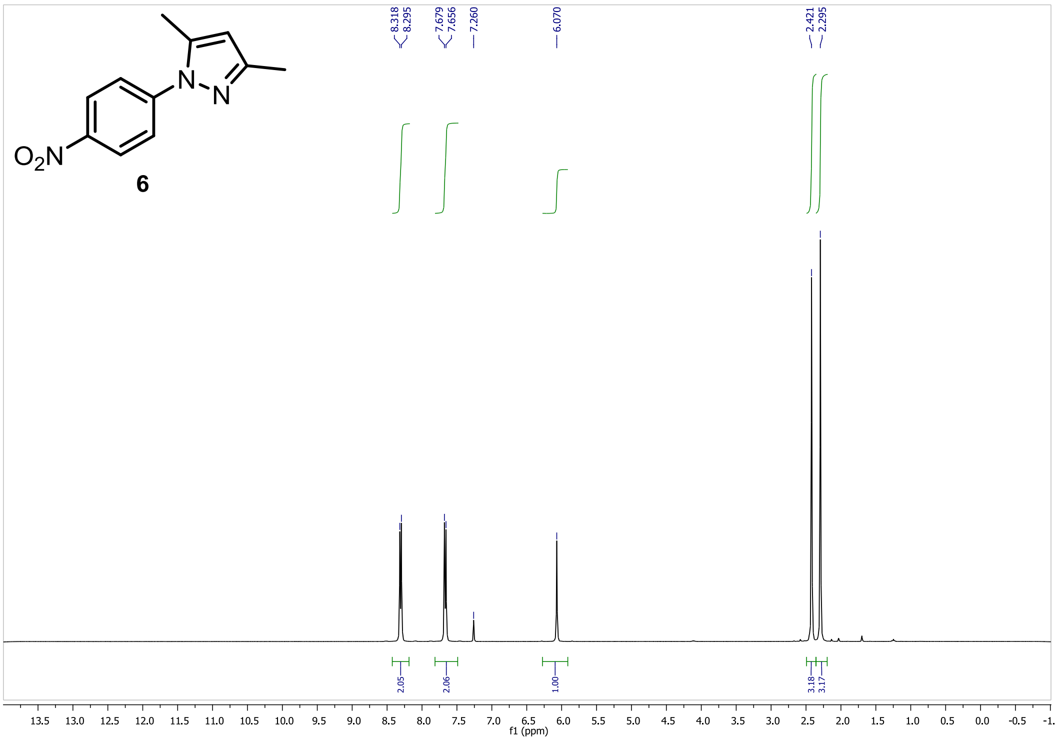

^{13}C NMR, 100 MHz, $CDCl_3$:

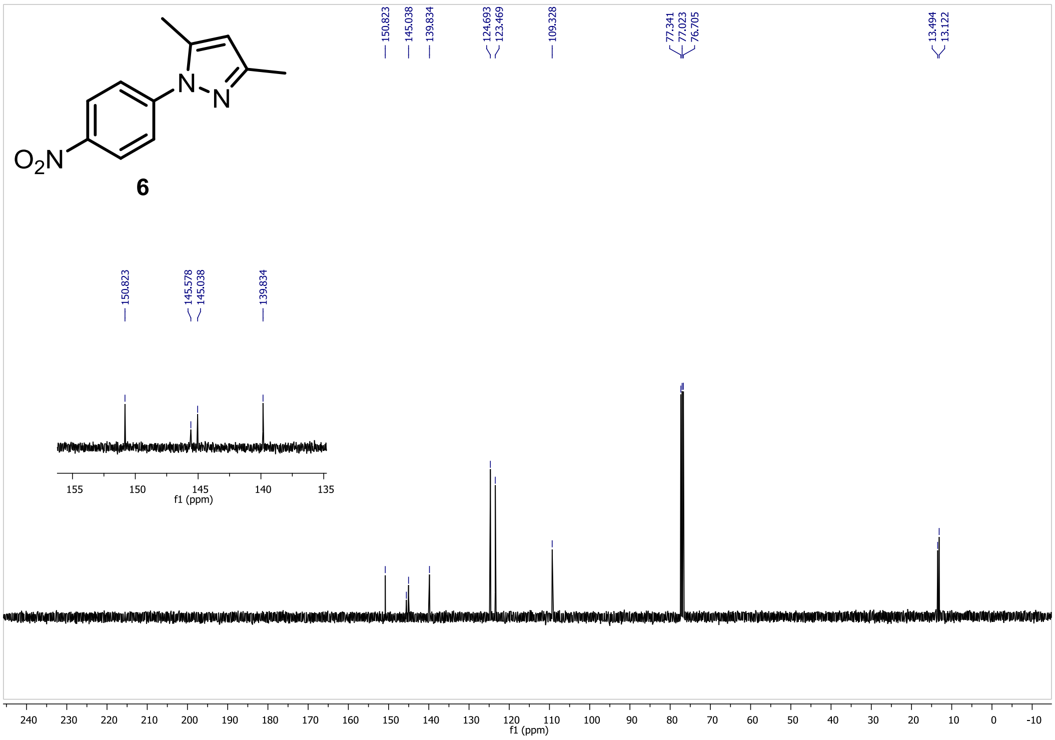

2-(3,5-dimethyl-1*H*-pyrazol-1-yl)benzoic acid (7):

^{1}H NMR, 400 MHz, $CDCl_3$:

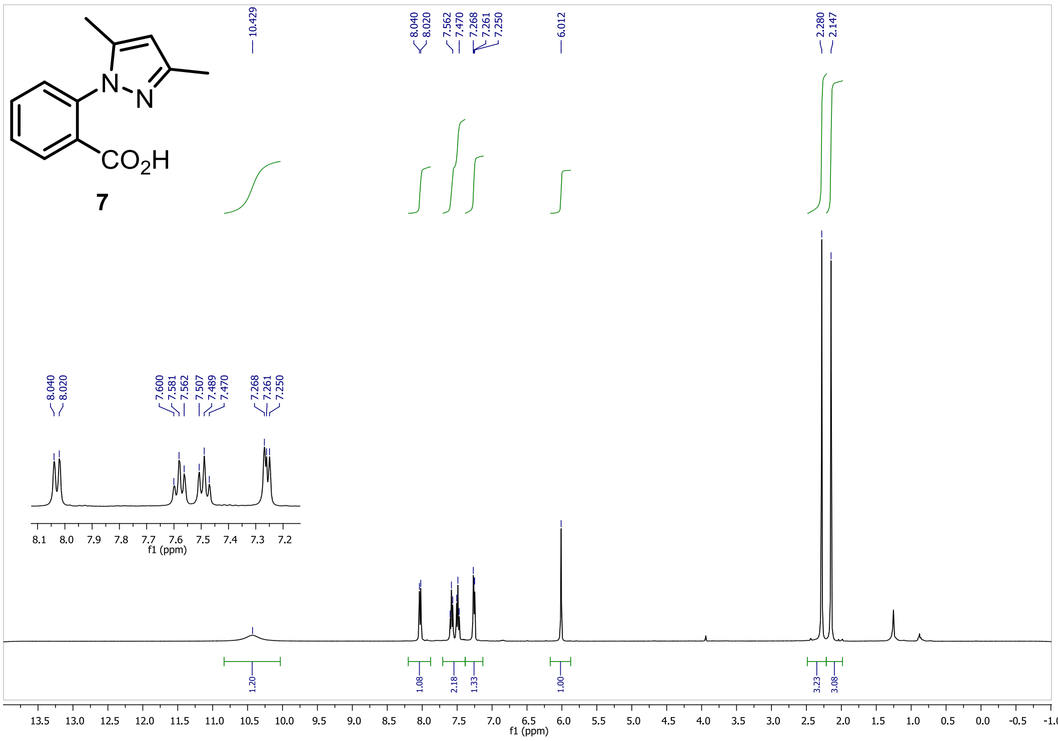

^{13}C NMR, 100 MHz, $CDCl_3$:

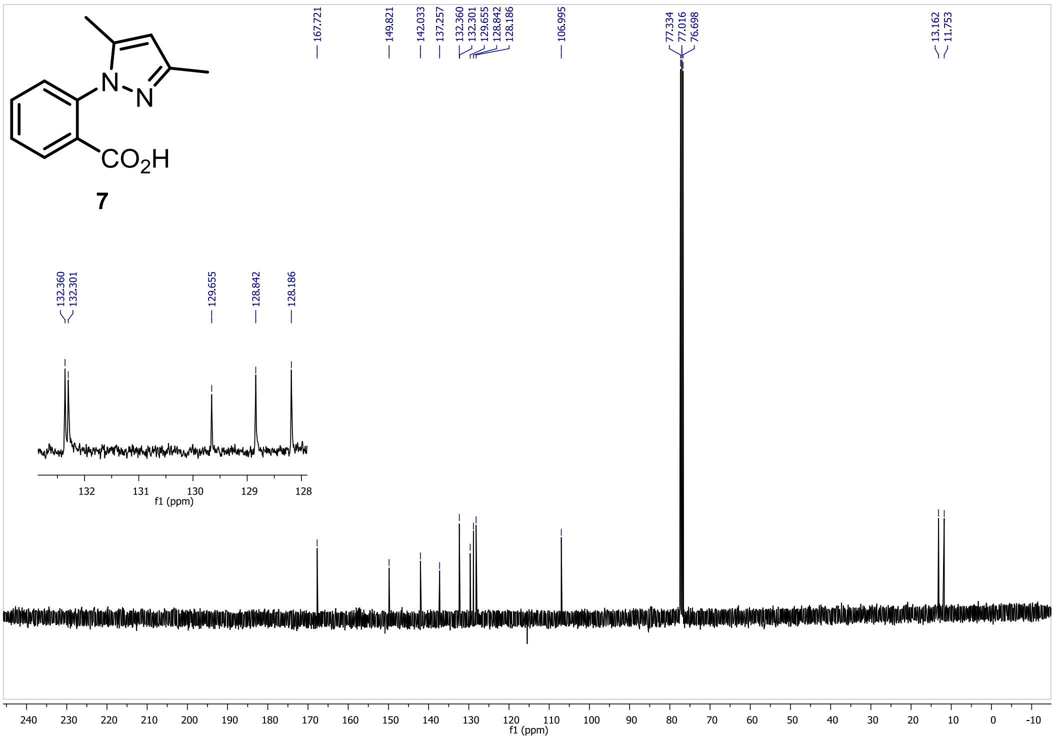

1-(3-bromophenyl)-3,5-dimethyl-1*H*-pyrazole (8):

^{1}H NMR, 400 MHz, $CDCl_3$:

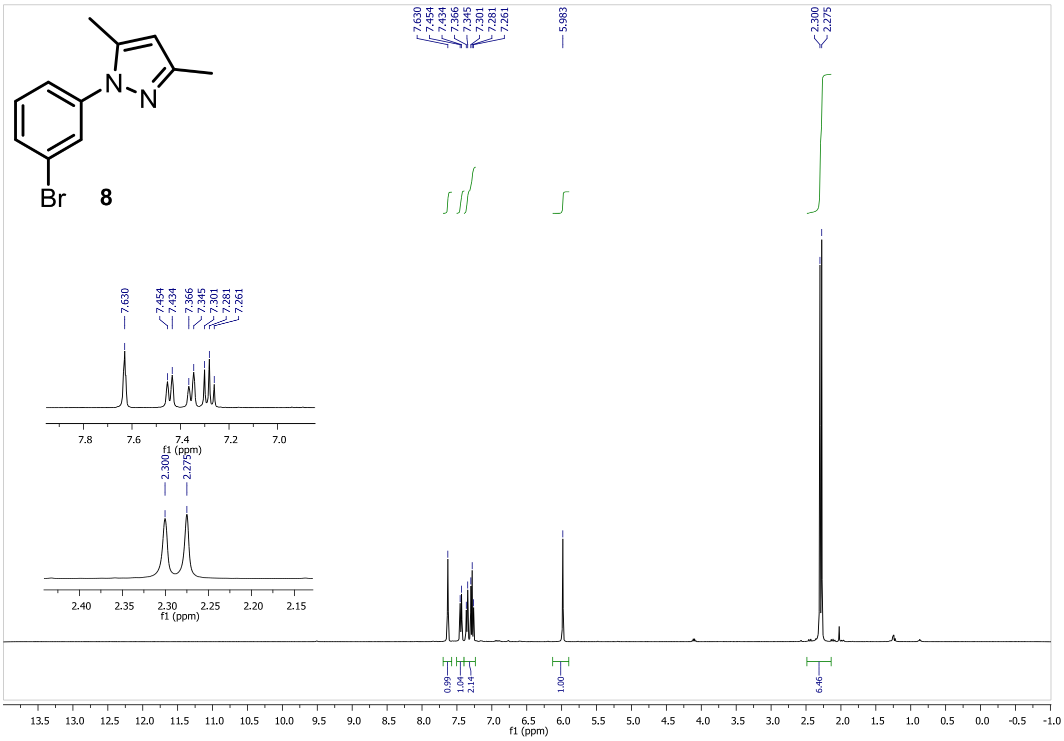

^{13}C NMR, 100 MHz, $CDCl_3$:

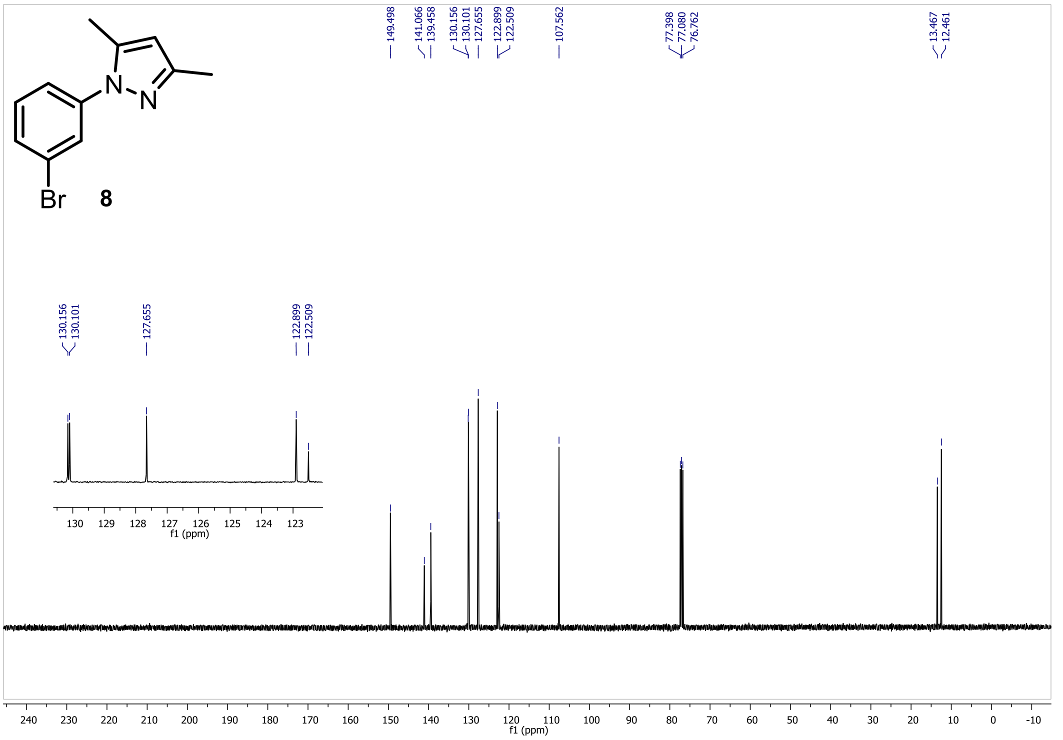

2-(4-(3,5-dimethyl-1*H*-pyrazol-1-yl)phenyl)ethanol (9):

^{1}H NMR, 400 MHz, $CDCl_3$:

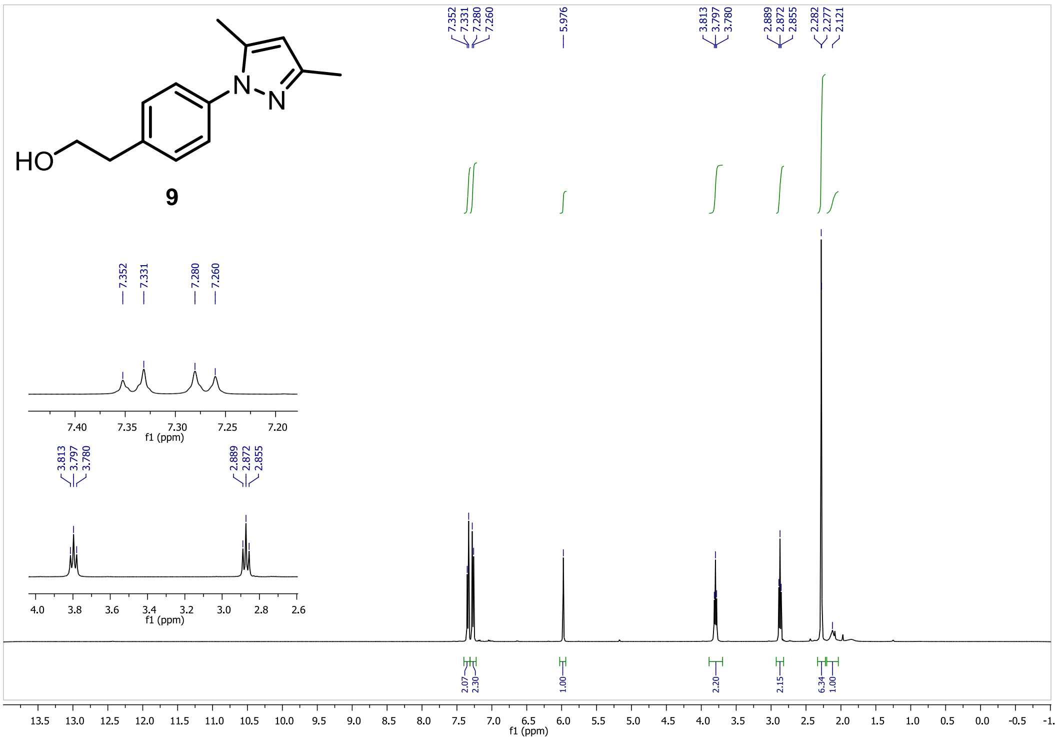

^{13}C NMR, 100 MHz, $CDCl_3$:

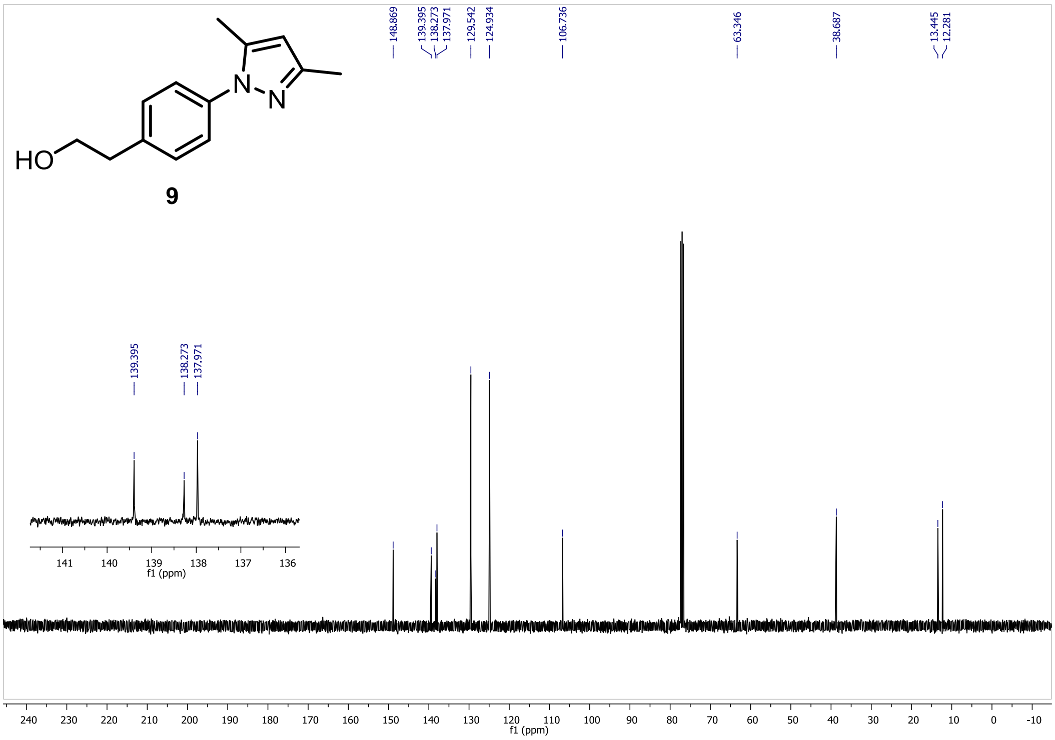

1-(4-fluorophenyl)-3,5-dimethyl-1*H*-pyrazole (10):

^{1}H NMR, 400 MHz, $CDCl_3$:

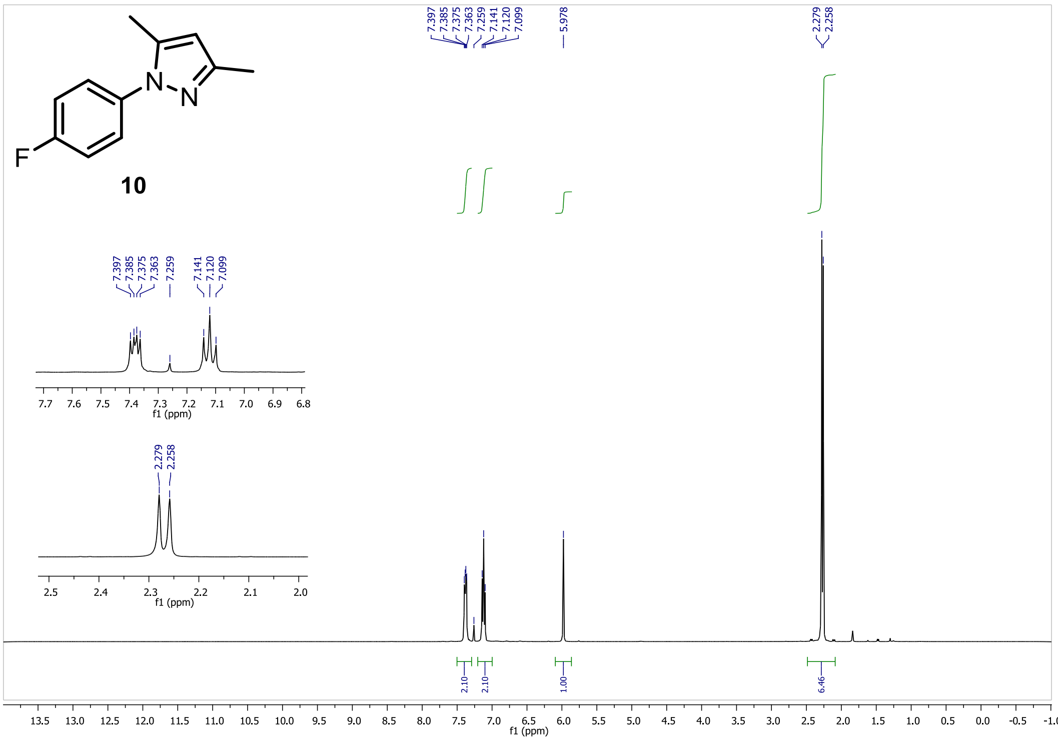

^{13}C NMR, 100 MHz, $CDCl_3$:

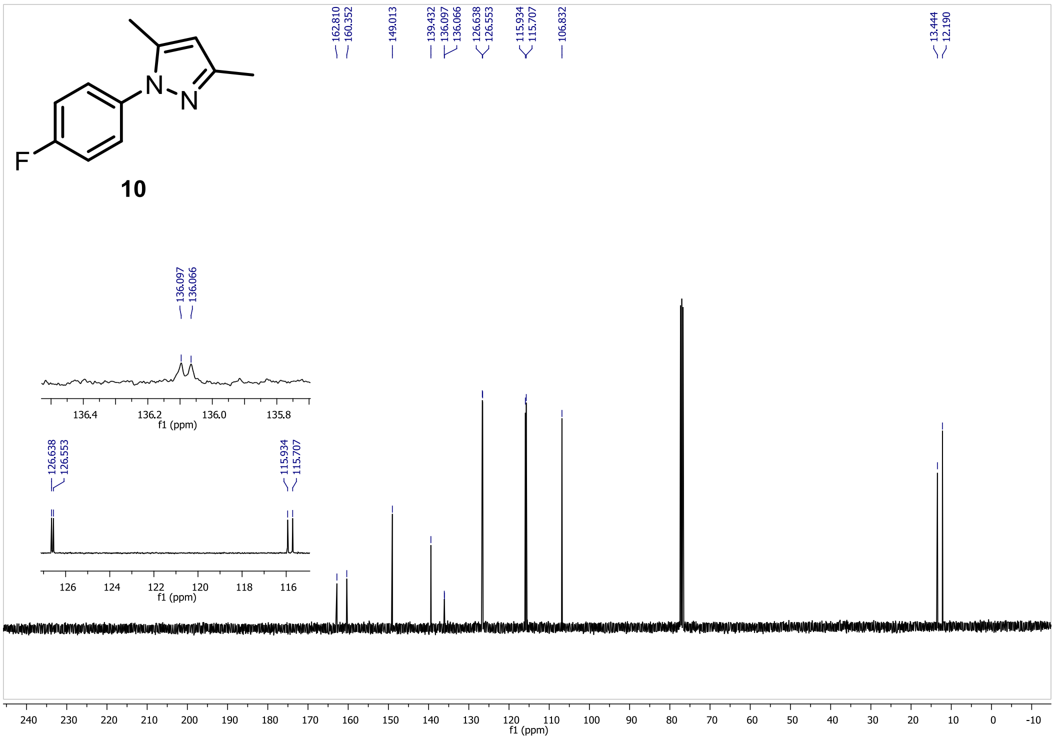

^{19}F NMR, 376 MHz, $CDCl_3$:

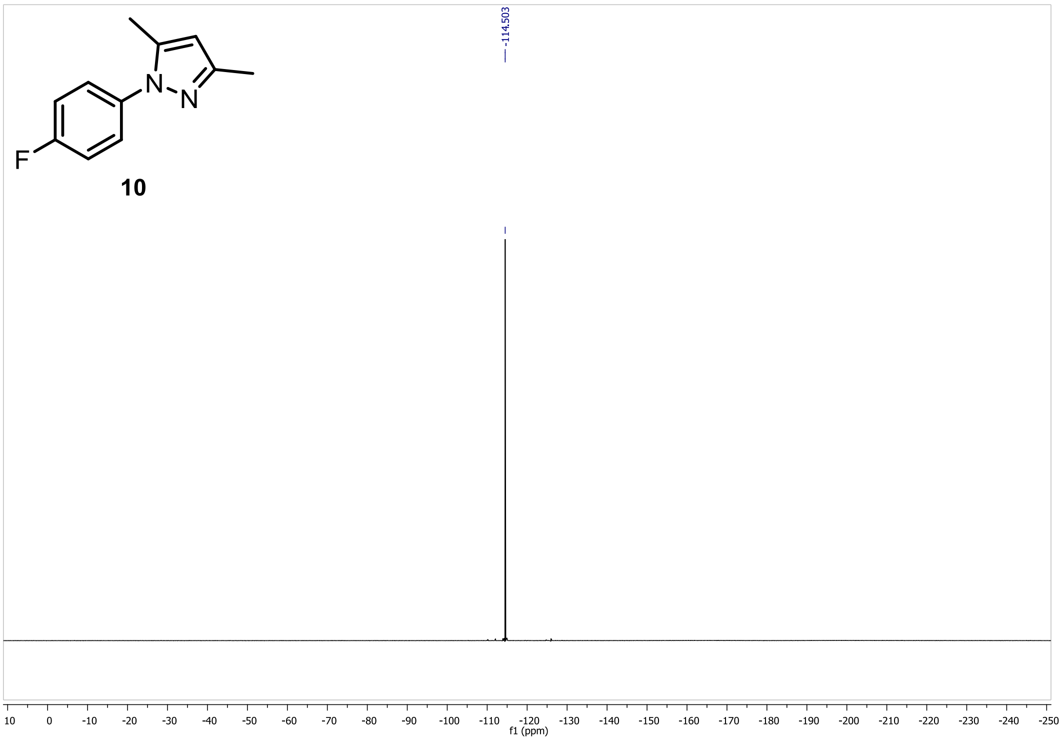

2-chloro-3-(3,5-dimethyl-1*H*-pyrazol-1-yl)pyridine (11):

^{1}H NMR, 400 MHz, $CDCl_3$:

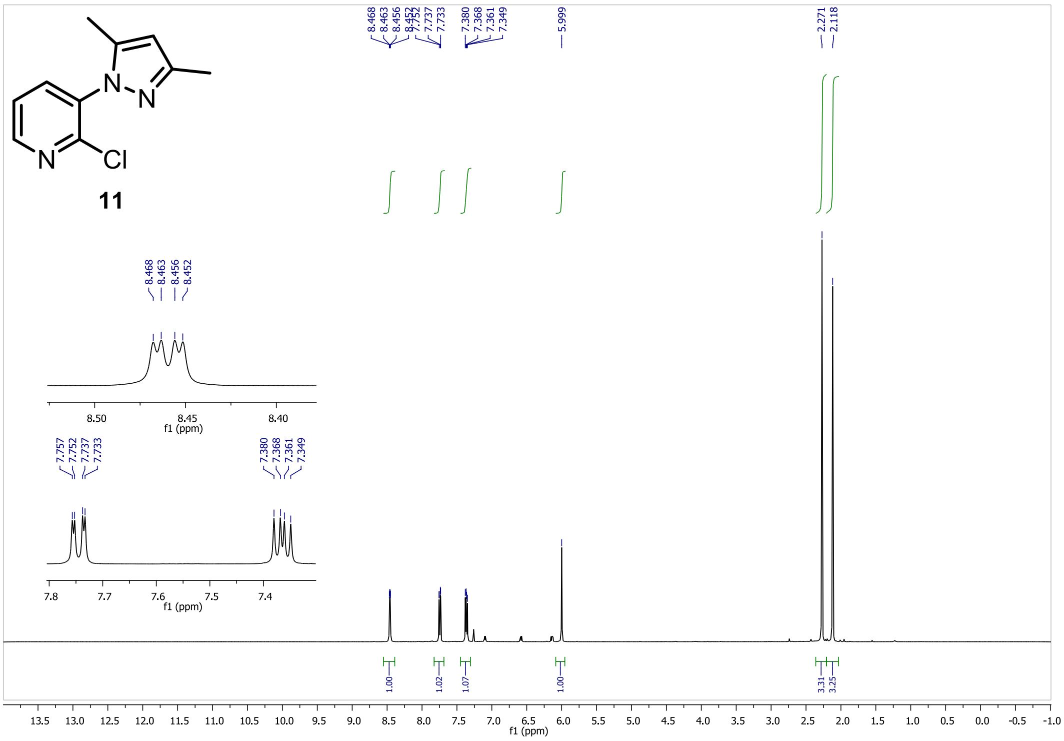

^{13}C NMR, 100 MHz, $CDCl_3$:

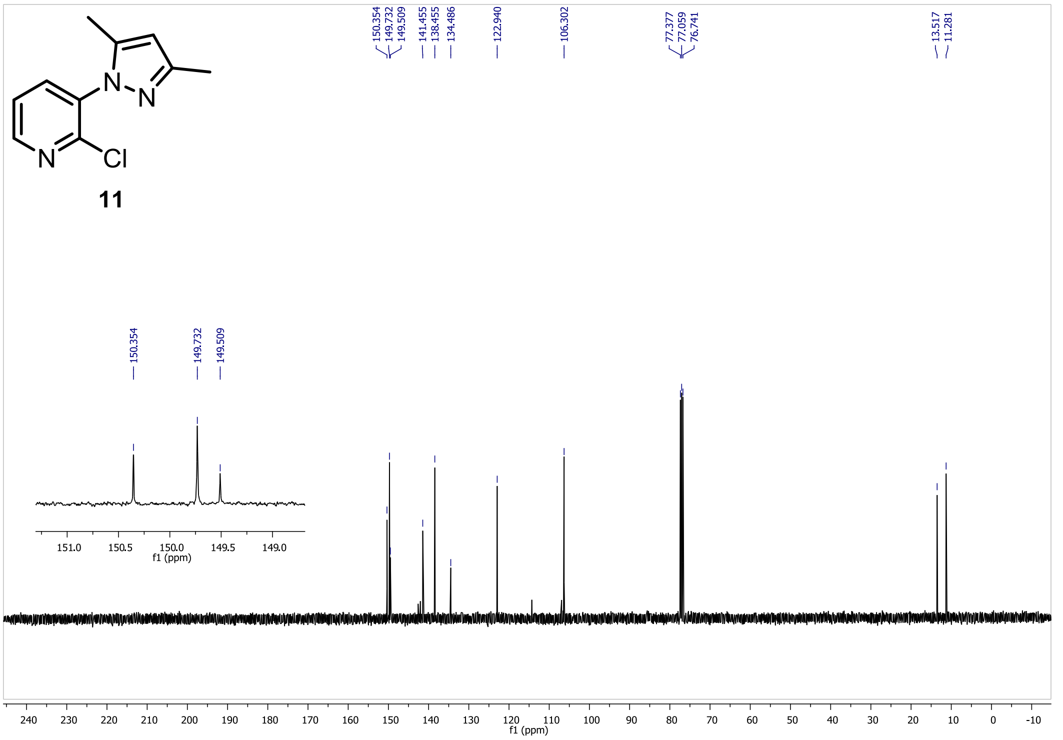

3,5-dimethyl-1-(4-(pentafluorosulfanyl)phenyl)-1*H*-pyrazole (12):

^{1}H NMR, 500 MHz, $CDCl_3$:

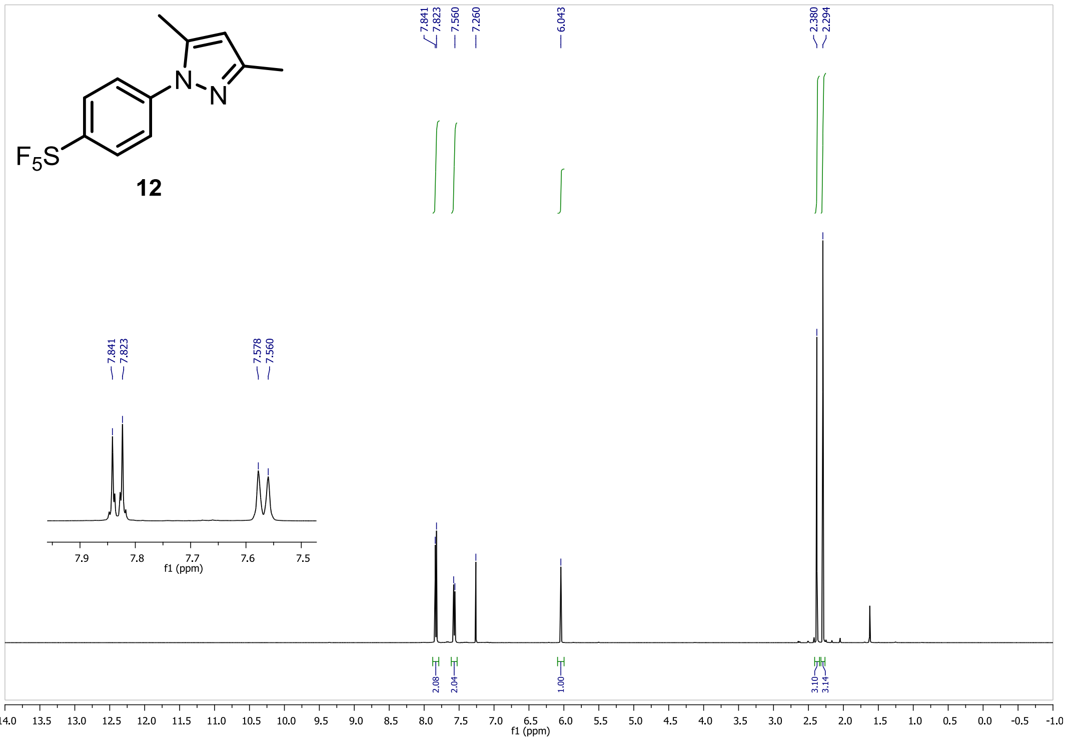

^{13}C NMR, 125 MHz, $CDCl_3$:

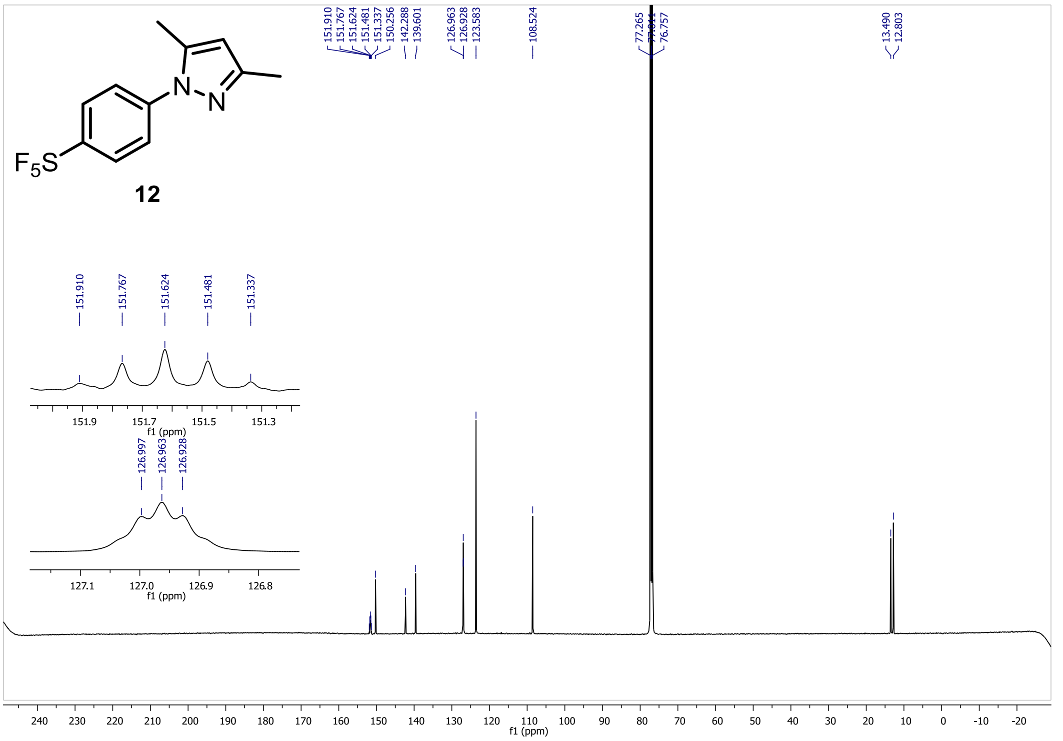

^{19}F NMR, 376 MHz, CDCl$_3$:

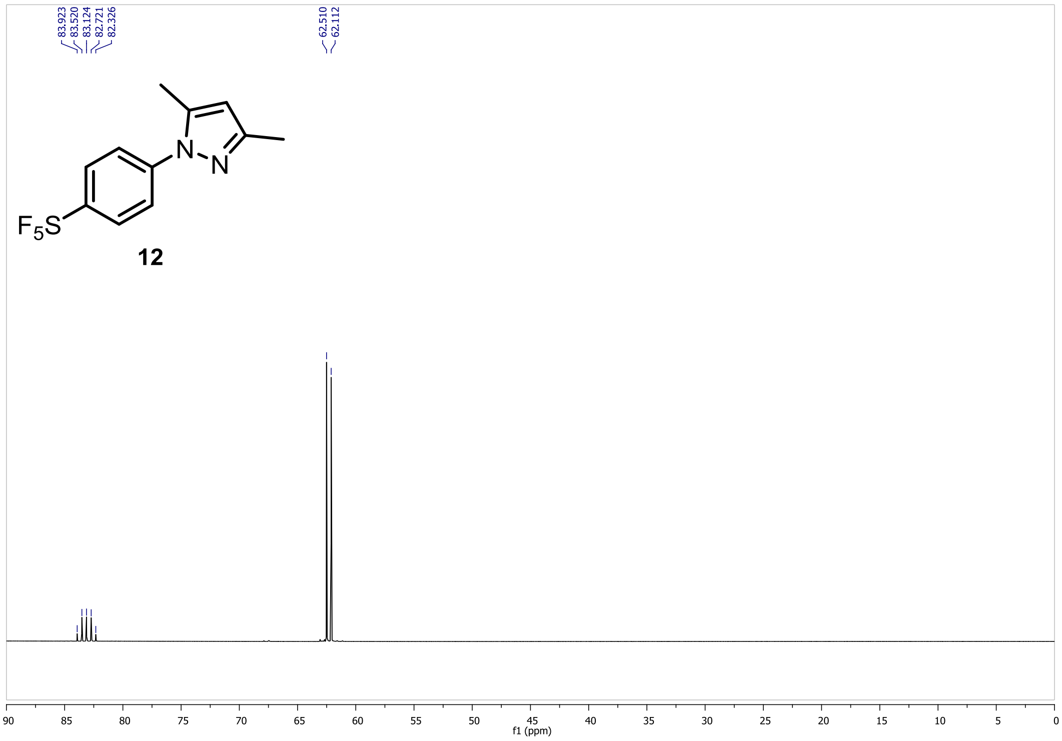

3-(3,5-dimethyl-1*H*-pyrazol-1-yl)quinoline (13):

^{1}H NMR, 400 MHz, $CDCl_3$:

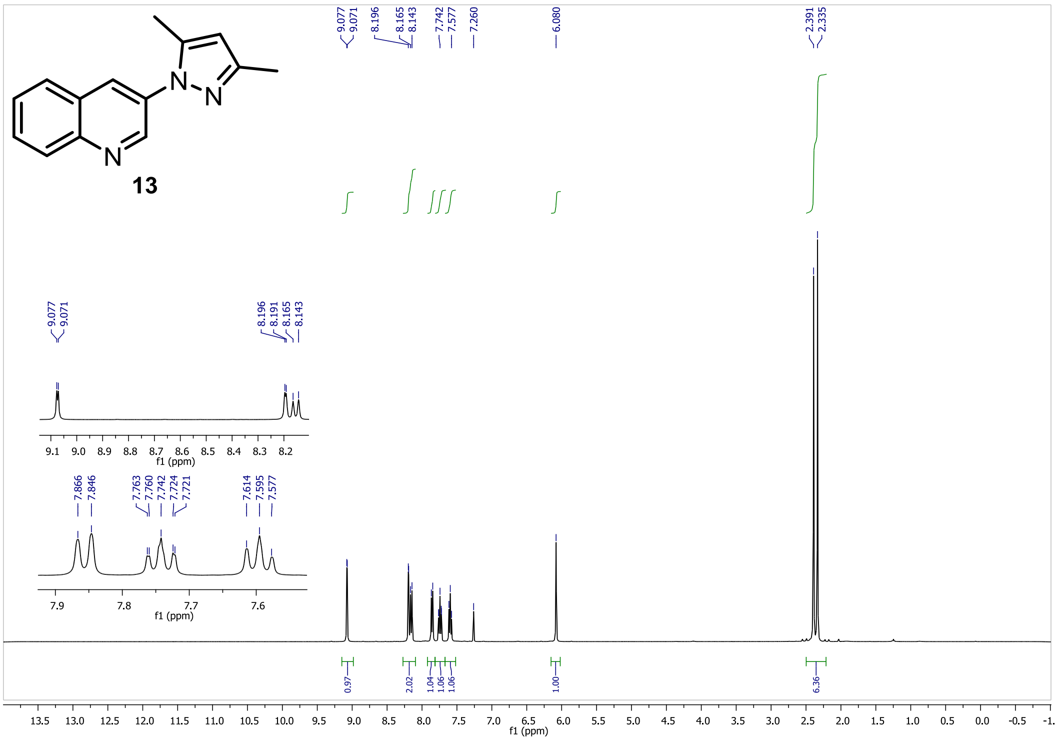

^{13}C NMR, 100 MHz, $CDCl_3$:

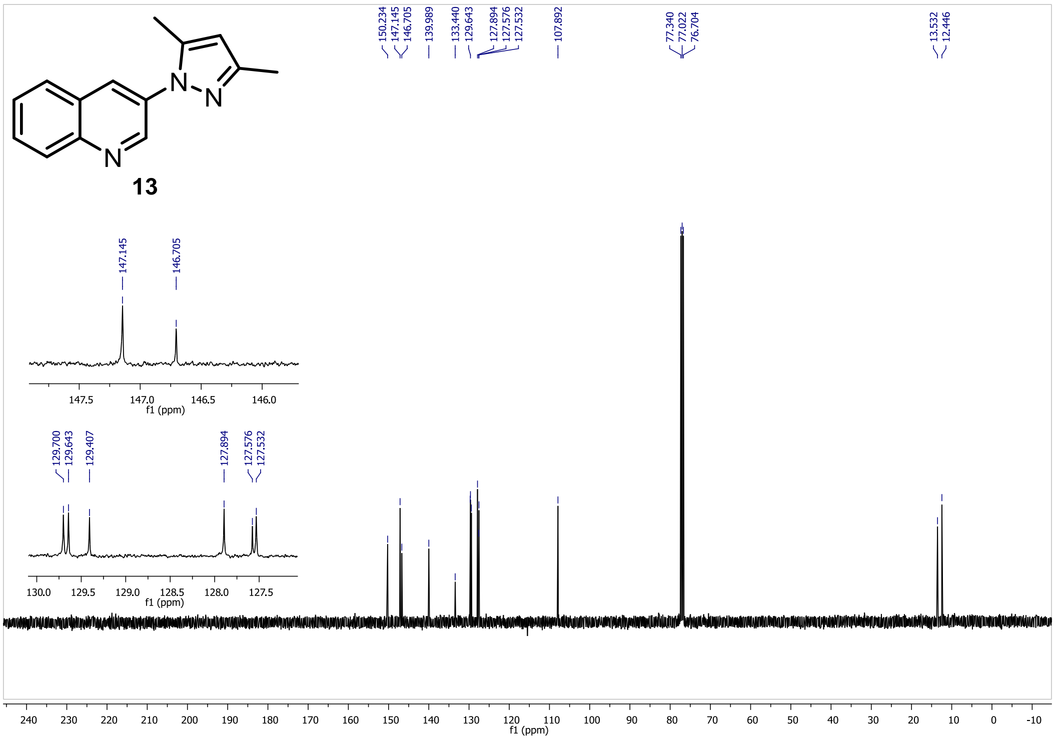

5-cyclopropyl-1-(4-(trifluoromethyl)phenyl)-1*H*-pyrazole-3-carboxylic acid (15):

^{1}H NMR, 600 MHz, $CDCl_3$:

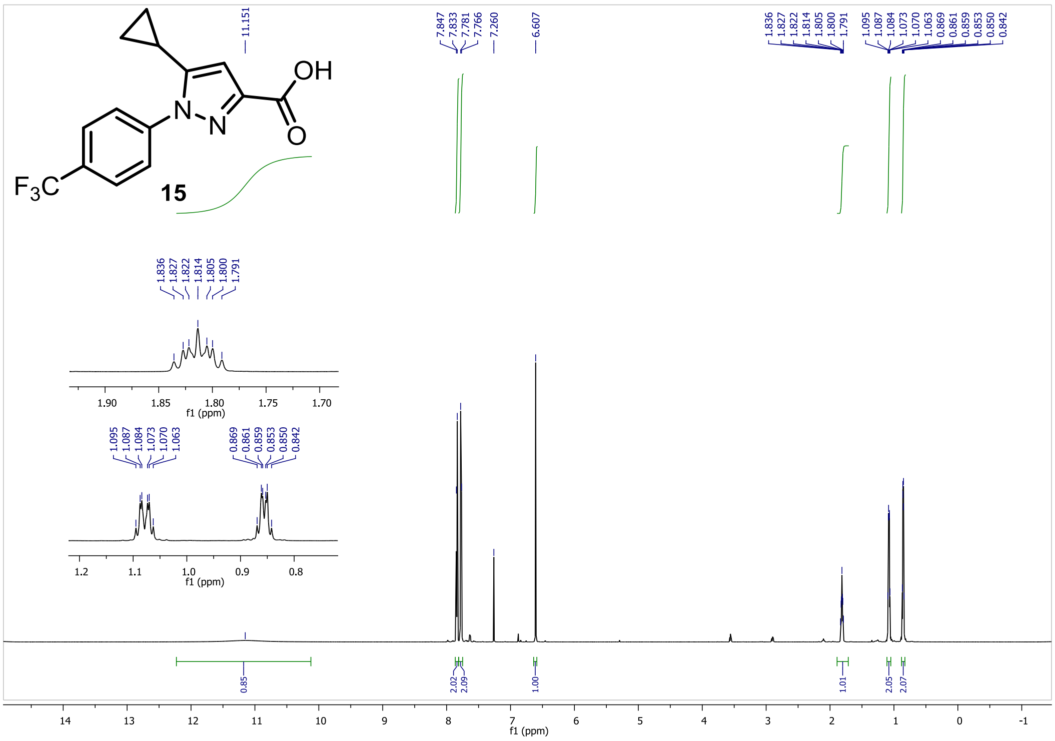

^{13}C NMR, 150 MHz, $CDCl_3$:

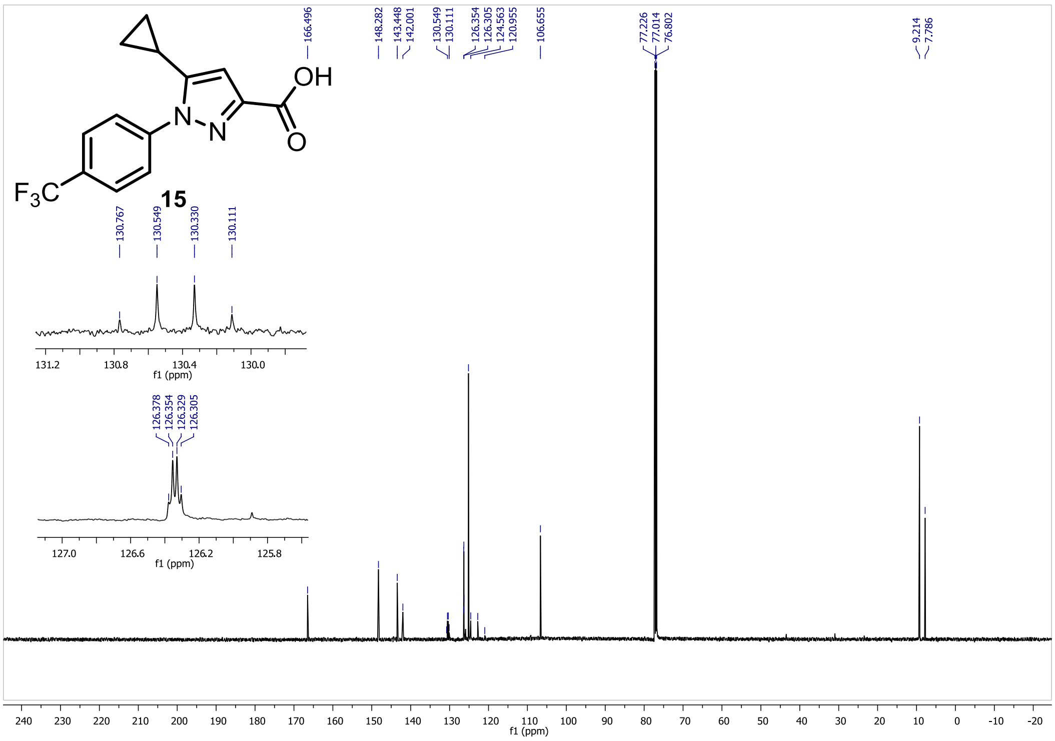

^{19}F NMR, 376 MHz, $CDCl_3$:

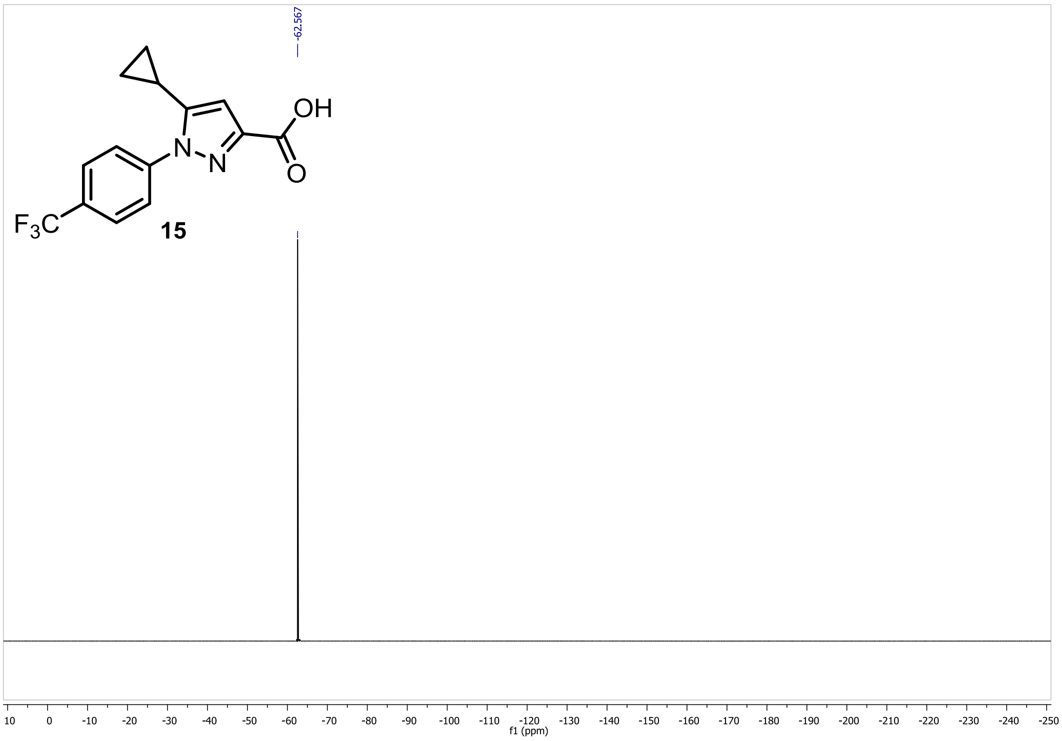

Ethyl 5-cyclopropyl-1-(4-(trifluoromethyl)phenyl)-1*H*-pyrazole-3-carboxylate (16):

^{1}H NMR, 600 MHz, $CDCl_3$:

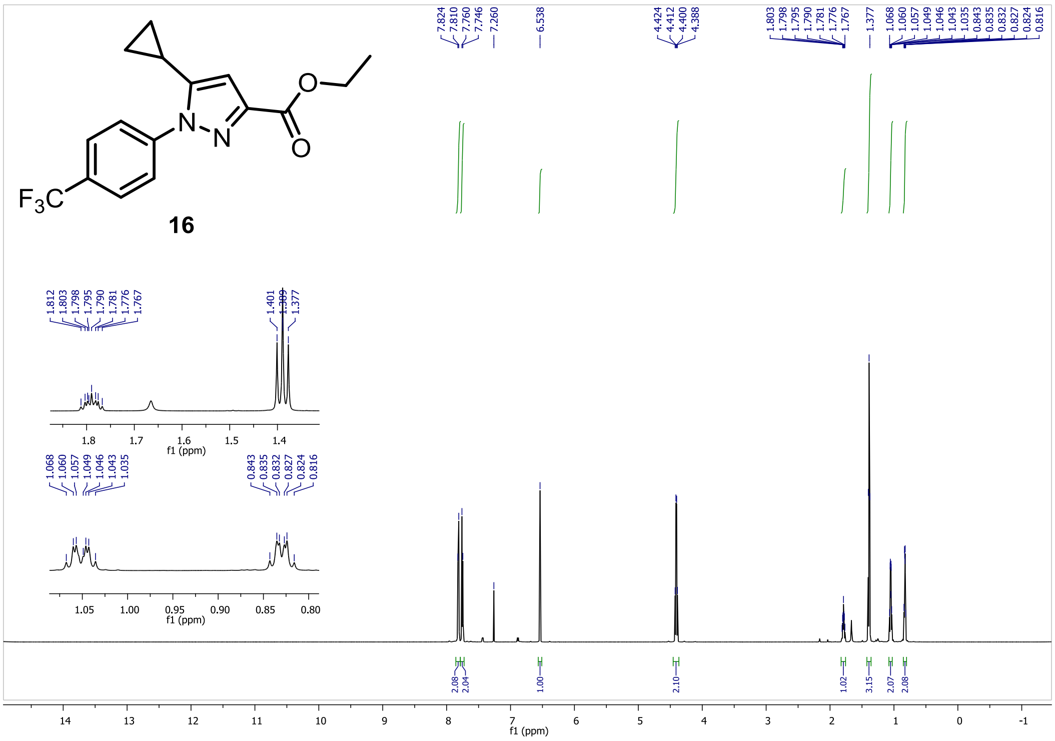

^{13}C NMR, 150 MHz, $CDCl_3$:

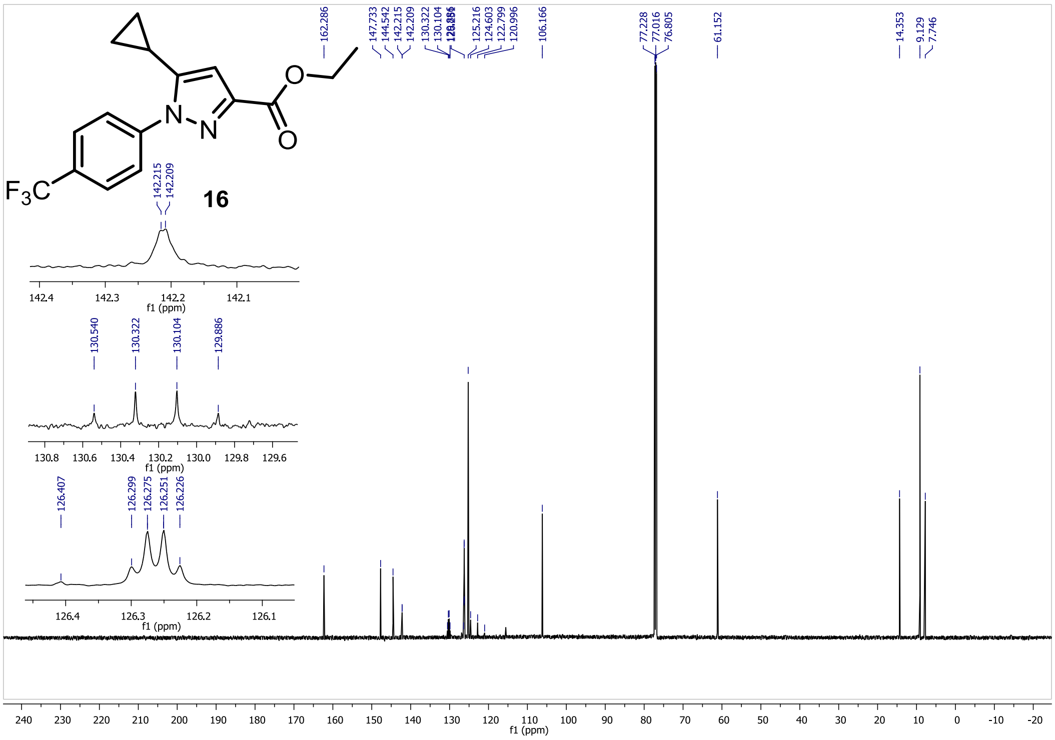

^{19}F NMR, 376 MHz, $CDCl_3$:

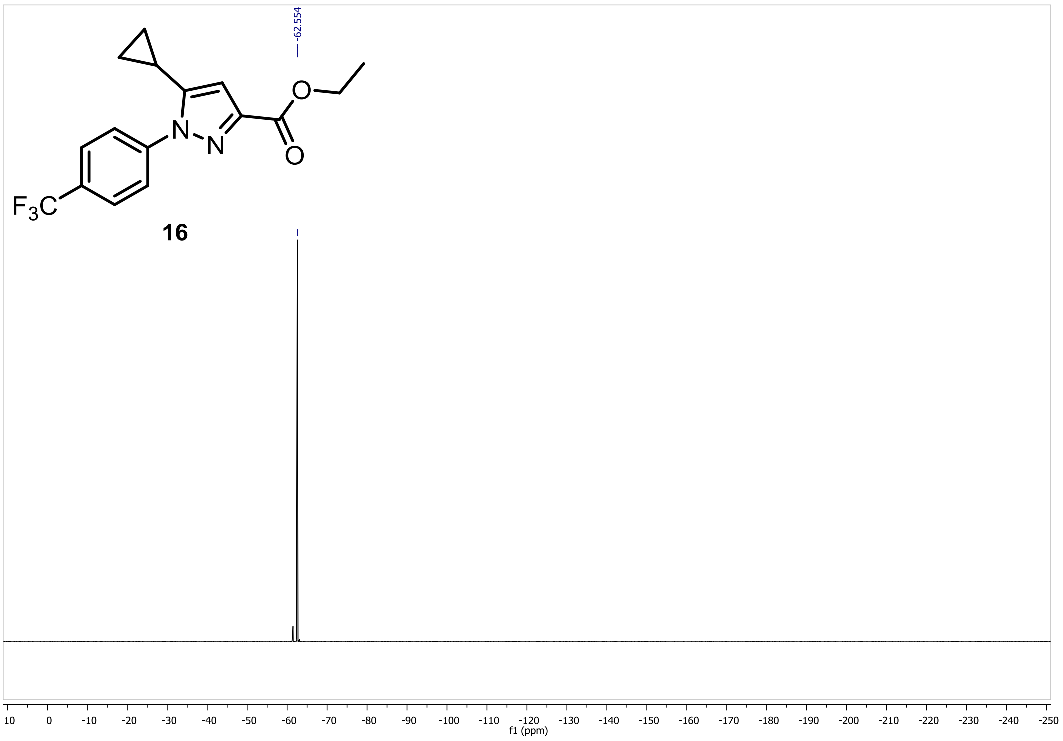

3-methyl-5-phenyl-1-(4-(trifluoromethyl)phenyl)-1*H*-pyrazole (18):

^{1}H NMR, 600 MHz, CDCl$_3$:

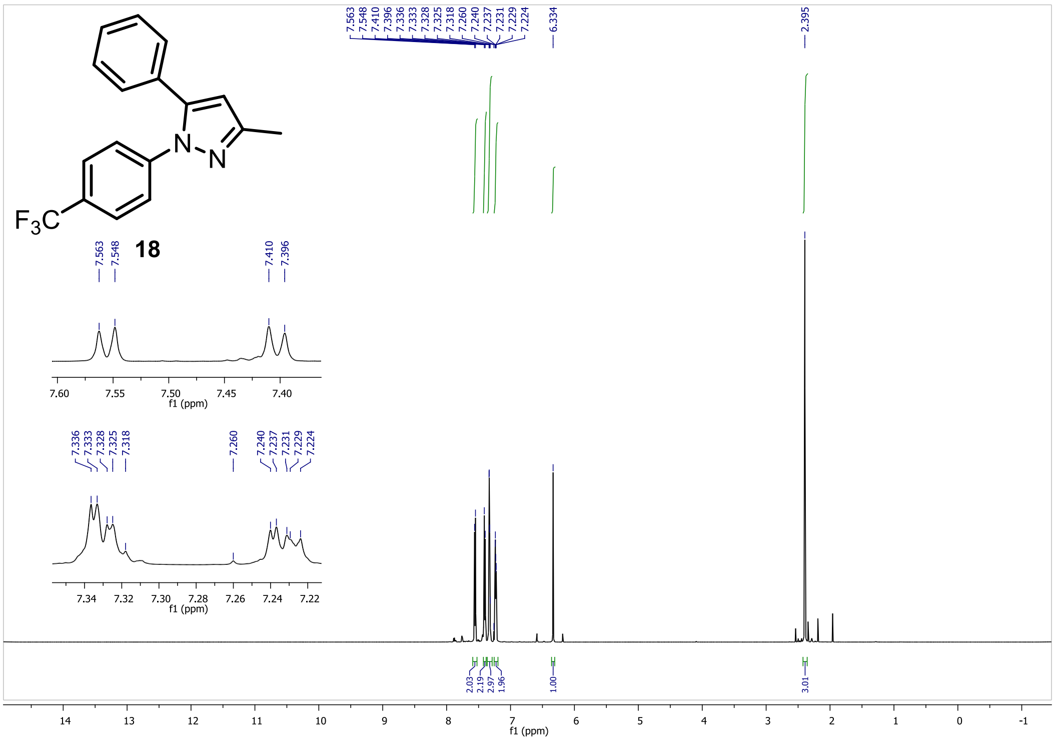

^{13}C NMR, 150 MHz, CDCl$_3$:

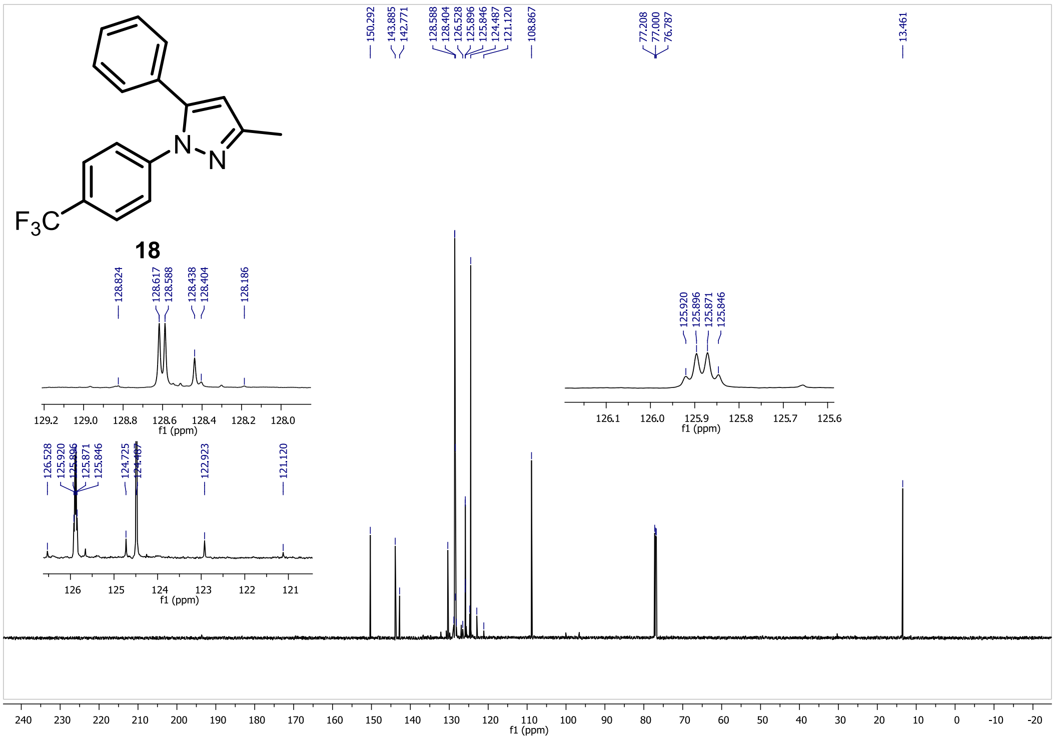

^{19}F NMR, 376 MHz, $CDCl_3$:

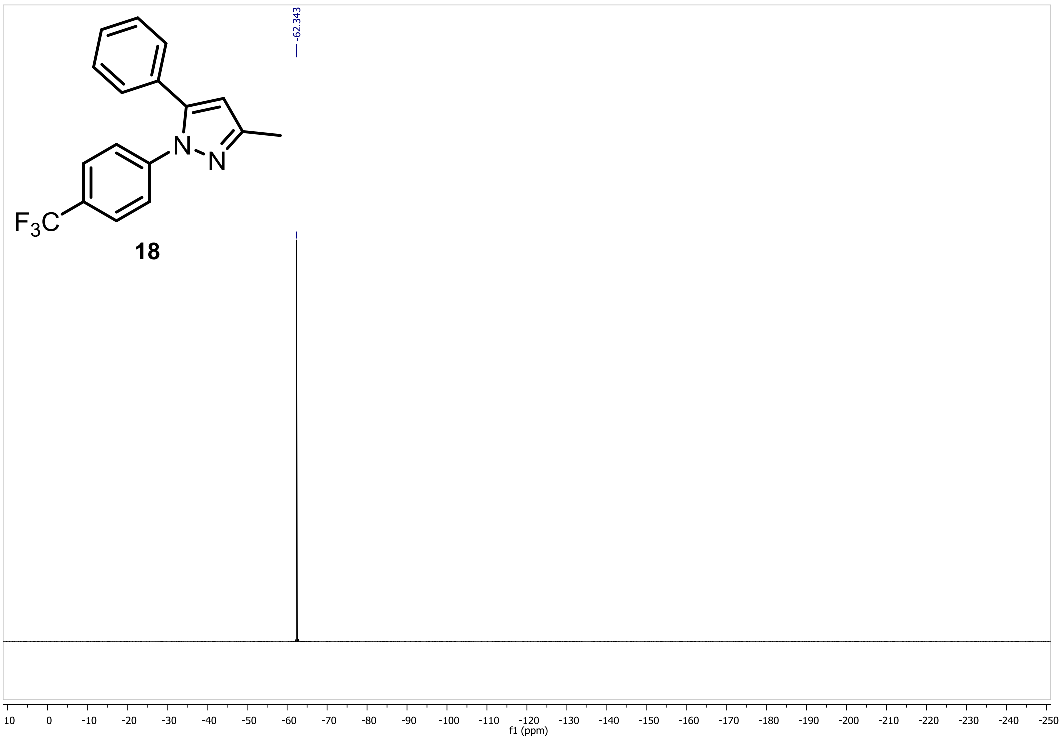

3-methyl-1-(4-(trifluoromethyl)phenyl)-1*H*-pyrazol-5-amine (20):

^{1}H NMR, 400 MHz, $CDCl_3$:

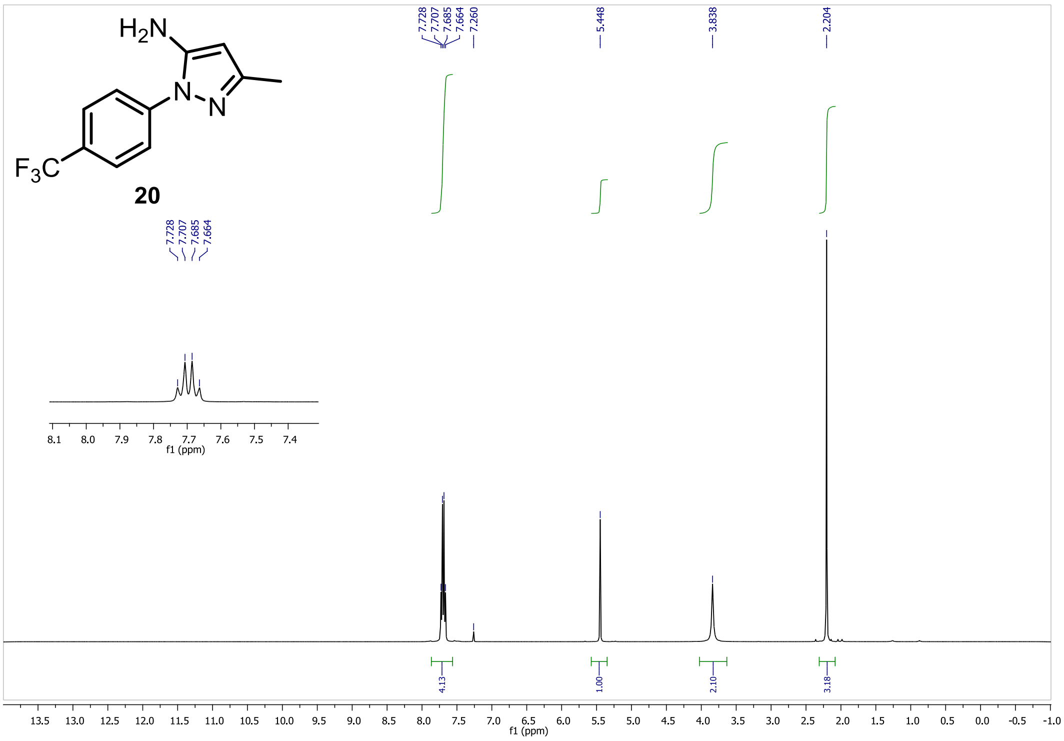

^{13}C NMR, 100 MHz, $CDCl_3$:

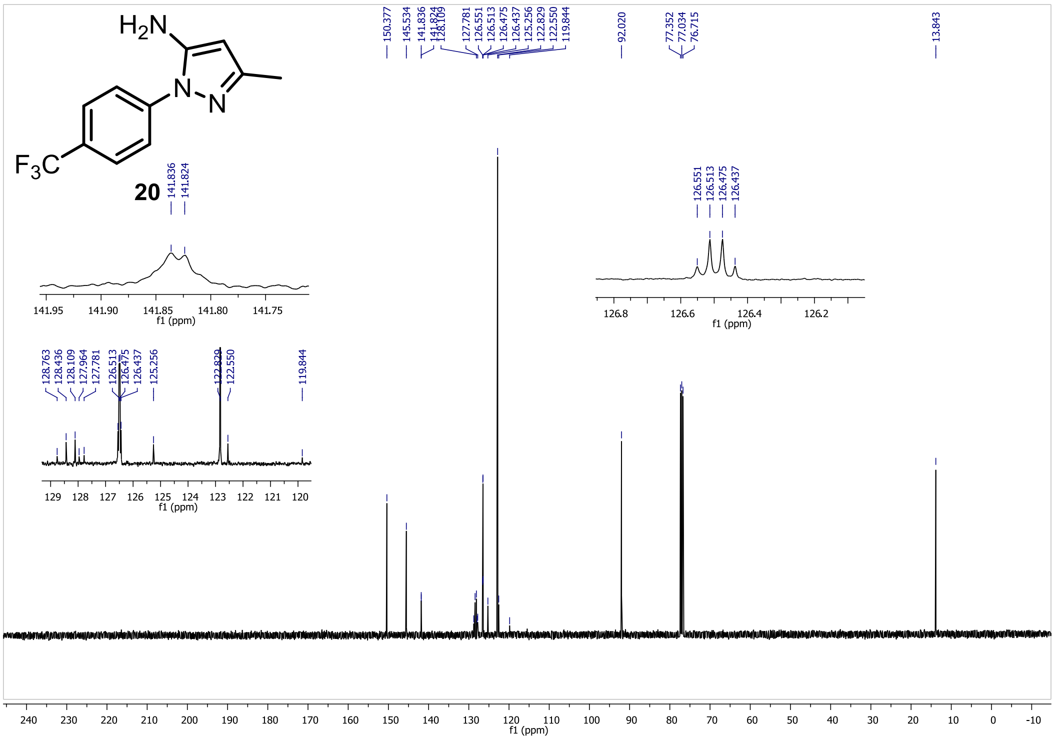

^{19}F NMR, 376 MHz, CDCl$_3$:

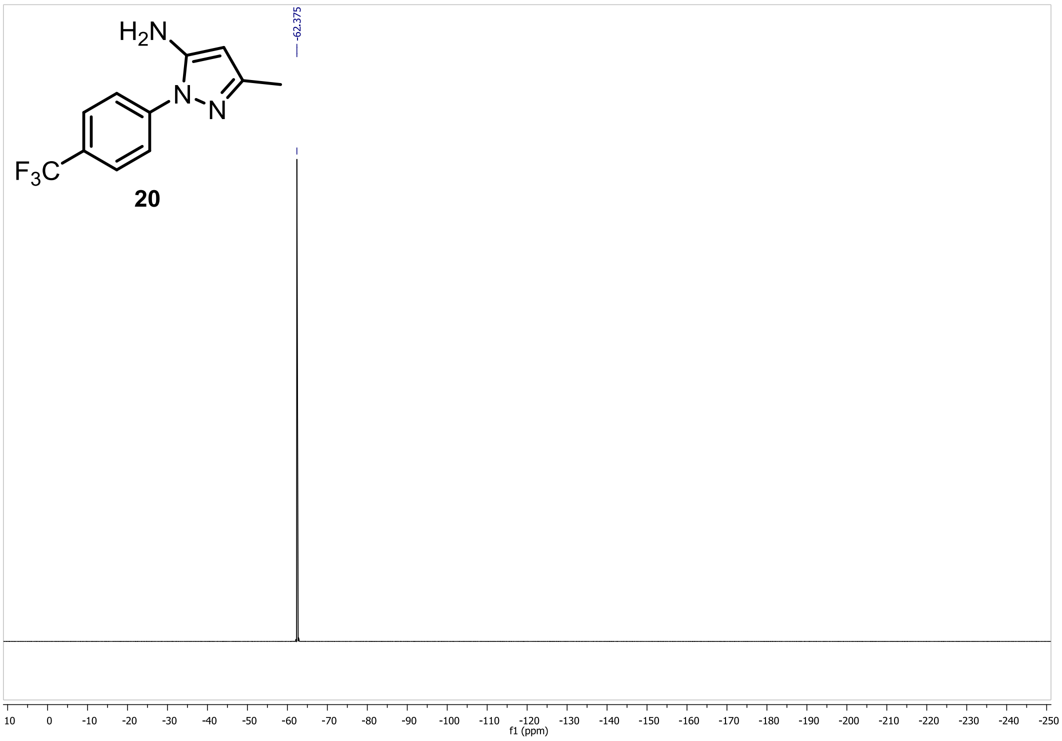

5-(thiophen-2-yl)-1-(4-(trifluoromethyl)phenyl)-1*H*-pyrazole (22a) and **3-(thiophen-2-yl)-1-(4-(trifluoromethyl)phenyl)-1*H*-pyrazole (22b):**

^{1}H NMR, 600 MHz, CDCl$_3$:

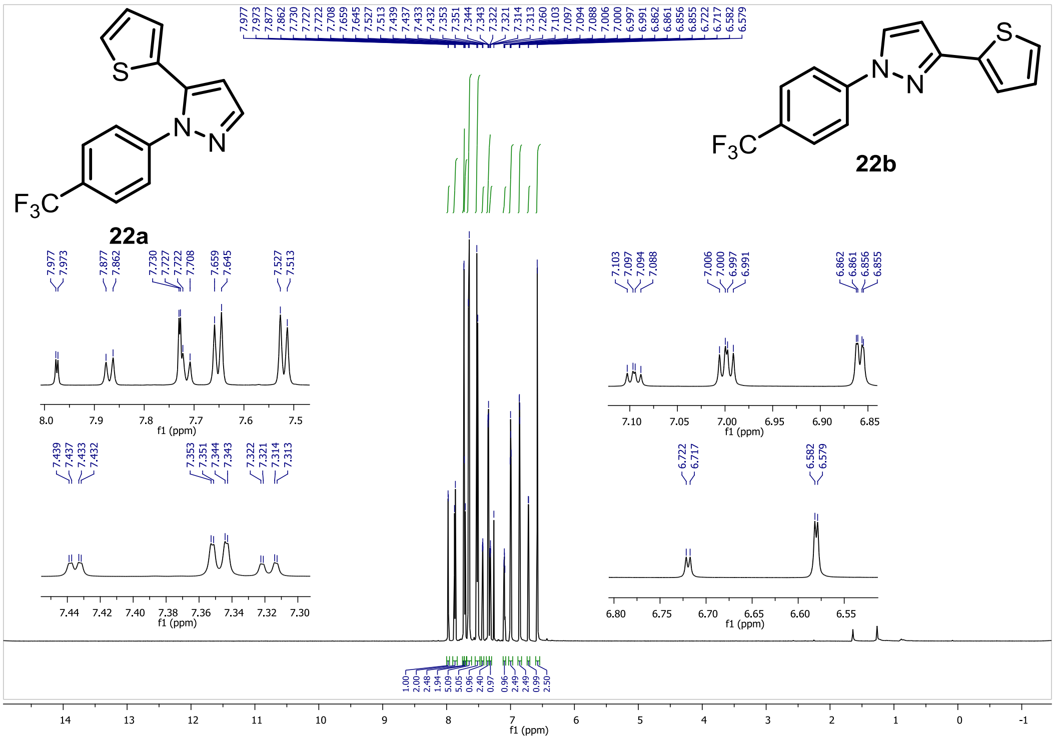

^{13}C NMR, 150 MHz, CDCl$_3$:

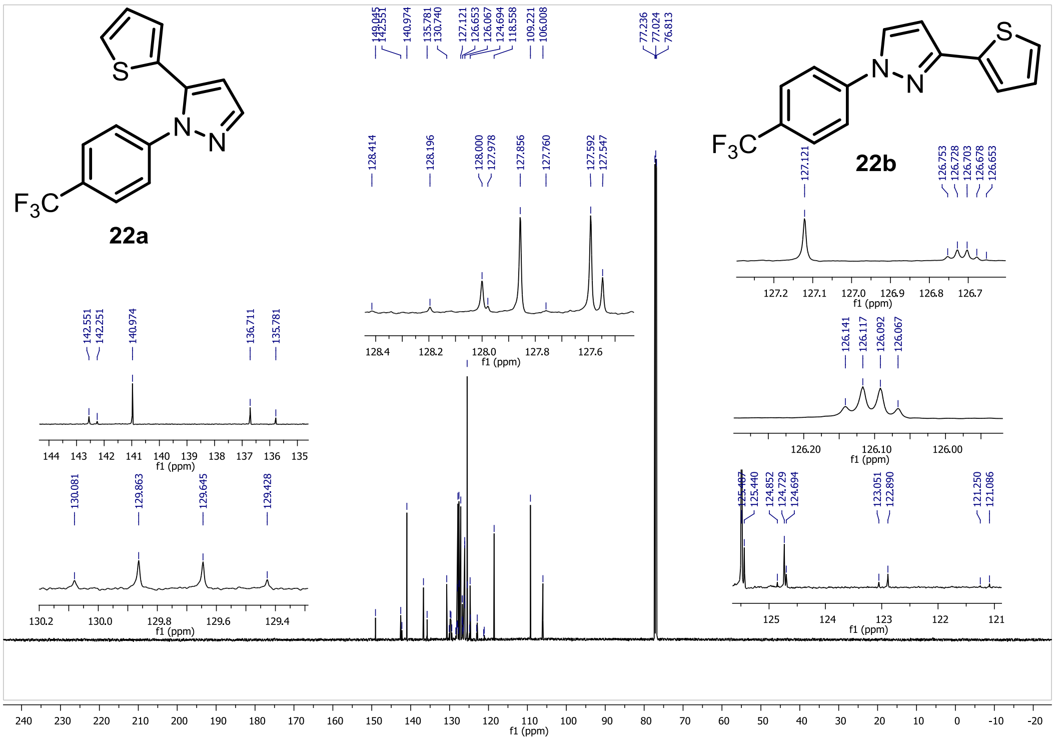

^{19}F NMR, 376 MHz, CDCl$_3$:

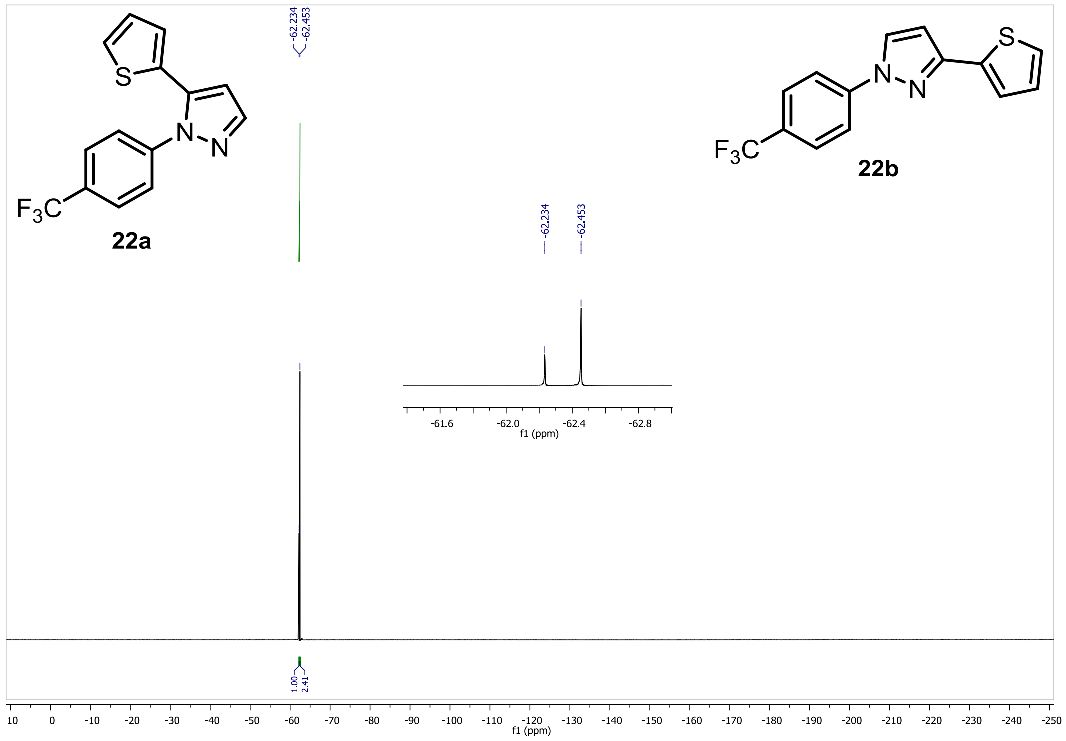

5-(furan-2-yl)-1-(4-(pentafluorosulfanyl)phenyl)-3-(trifluoromethyl)-1*H*-pyrazole (23):

^{1}H NMR, 500 MHz, CDCl$_3$:

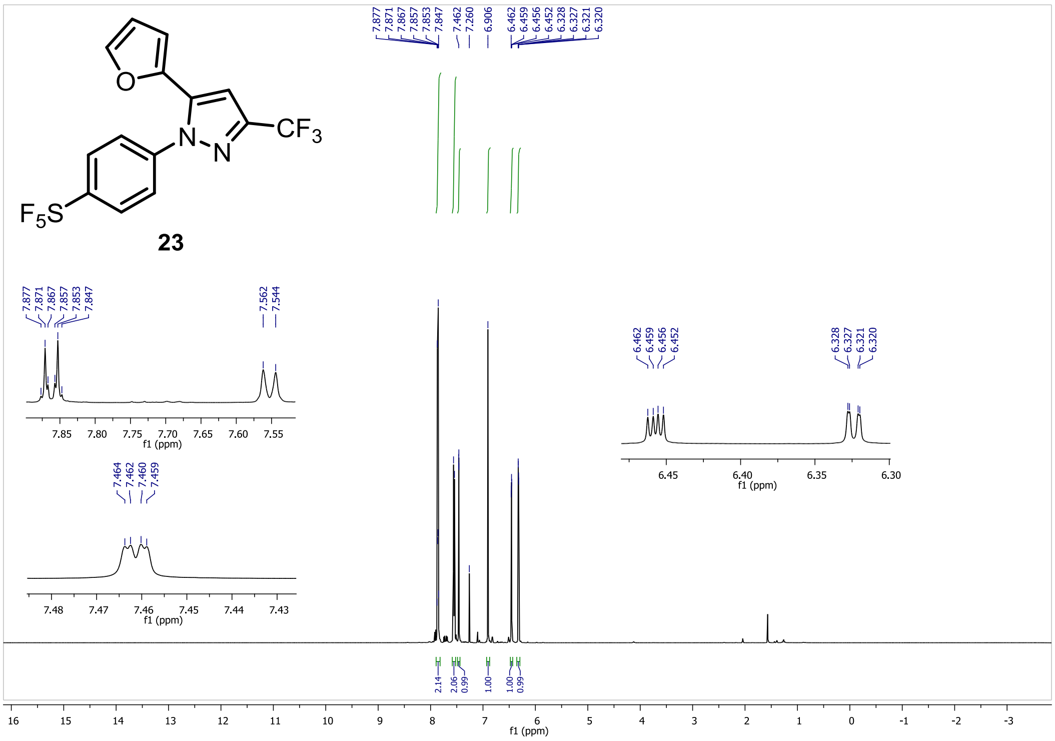

^{13}C NMR, 125 MHz, CDCl$_3$:

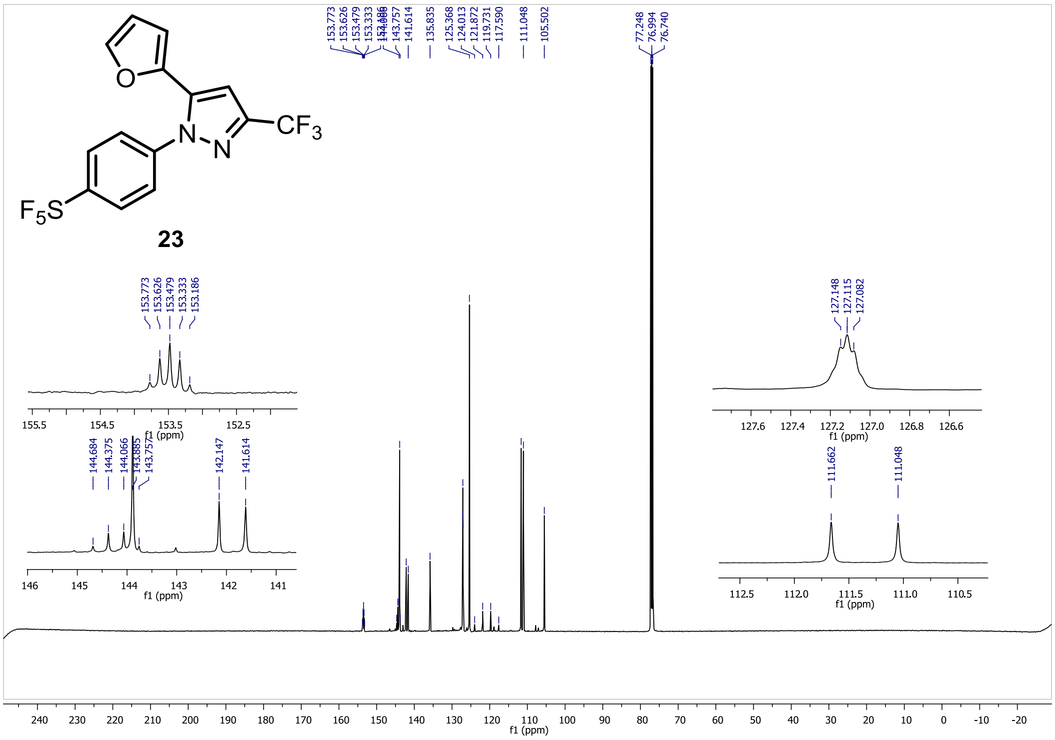

^{19}F NMR, 376 MHz, $CDCl_3$:

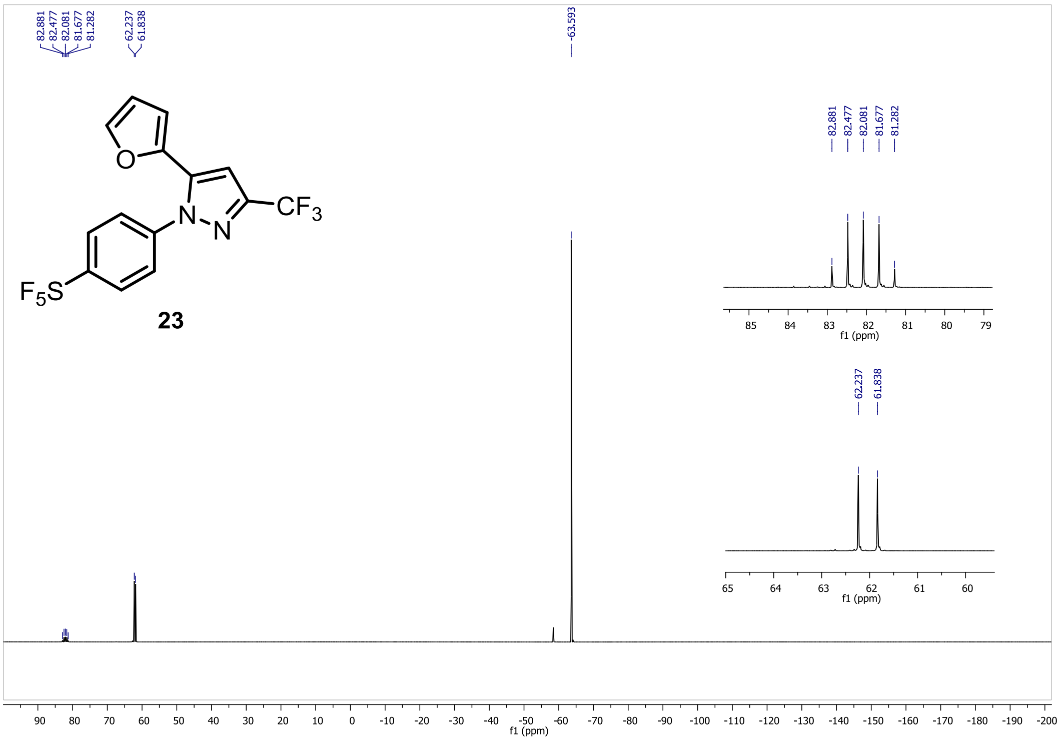

2-chloro-3-(5-phenyl-3-(trifluoromethyl)-1*H*-pyrazol-1-yl)pyridine (24):

^{1}H NMR, 600 MHz, $CDCl_3$:

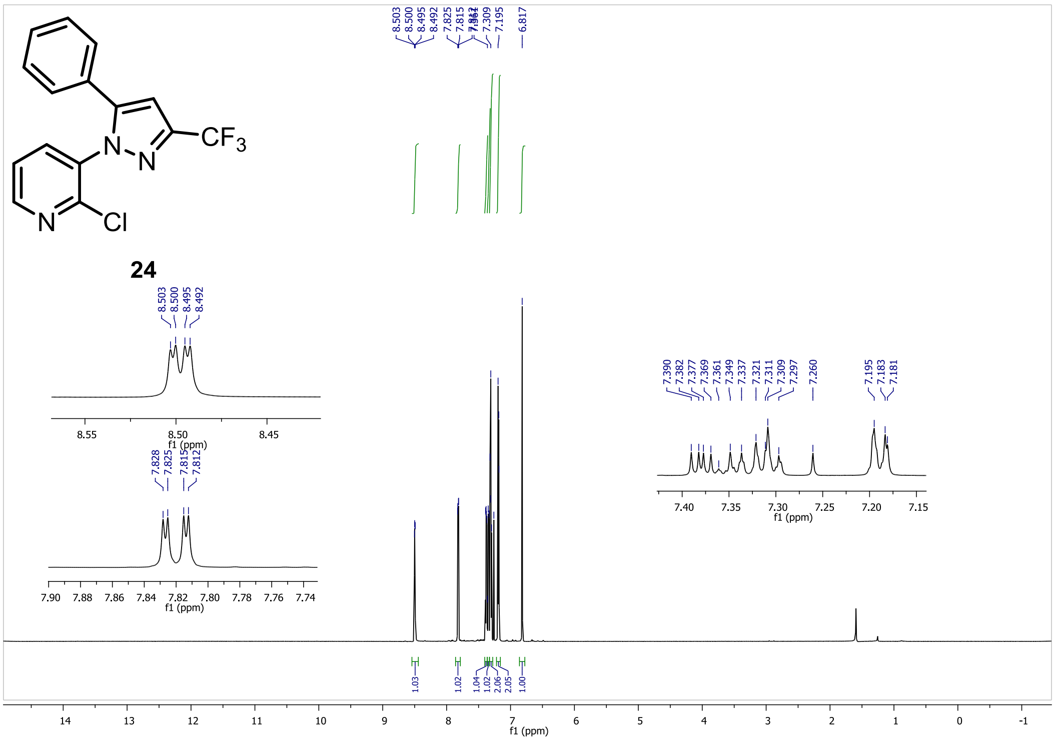

^{13}C NMR, 150 MHz, $CDCl_3$:

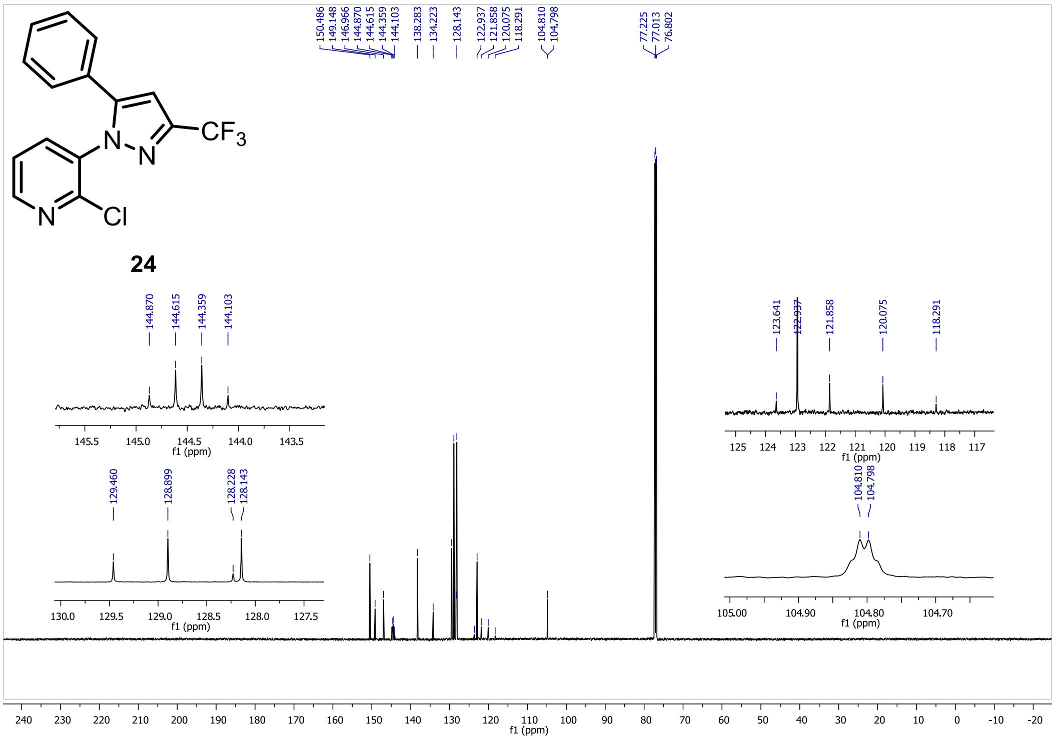

^{19}F NMR, 376 MHz, $CDCl_3$:

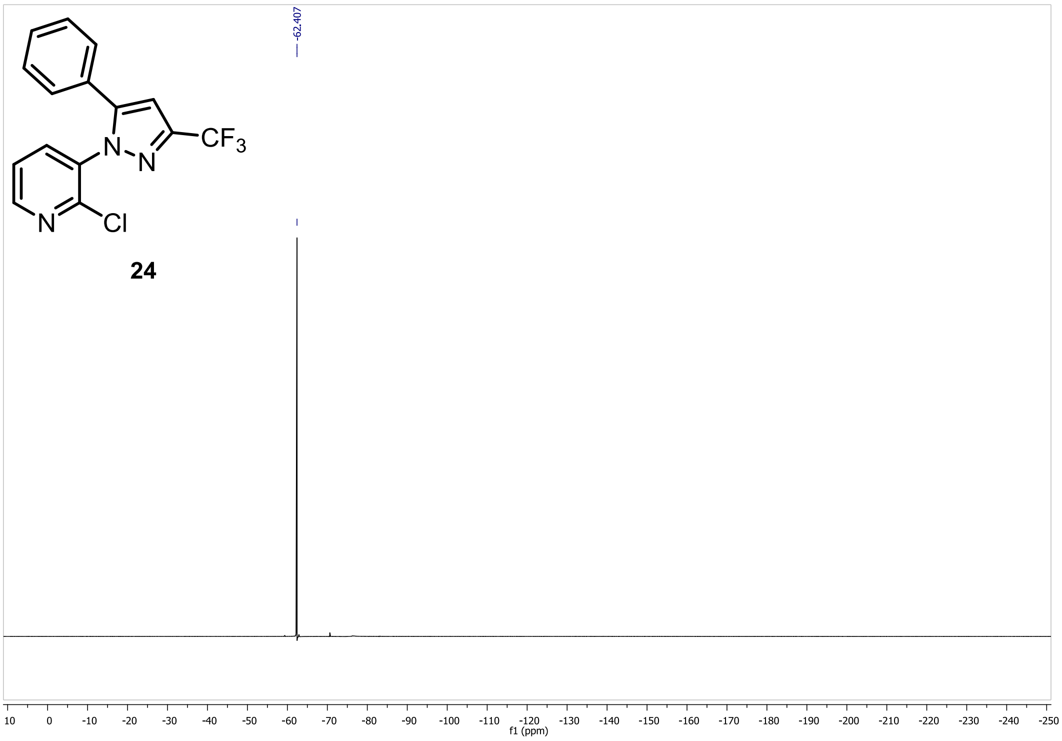

5-(furan-2-yl)-1-(4-nitrophenyl)-3-(trifluoromethyl)-1*H*-pyrazole (25):

^{1}H NMR, 600 MHz, $CDCl_3$:

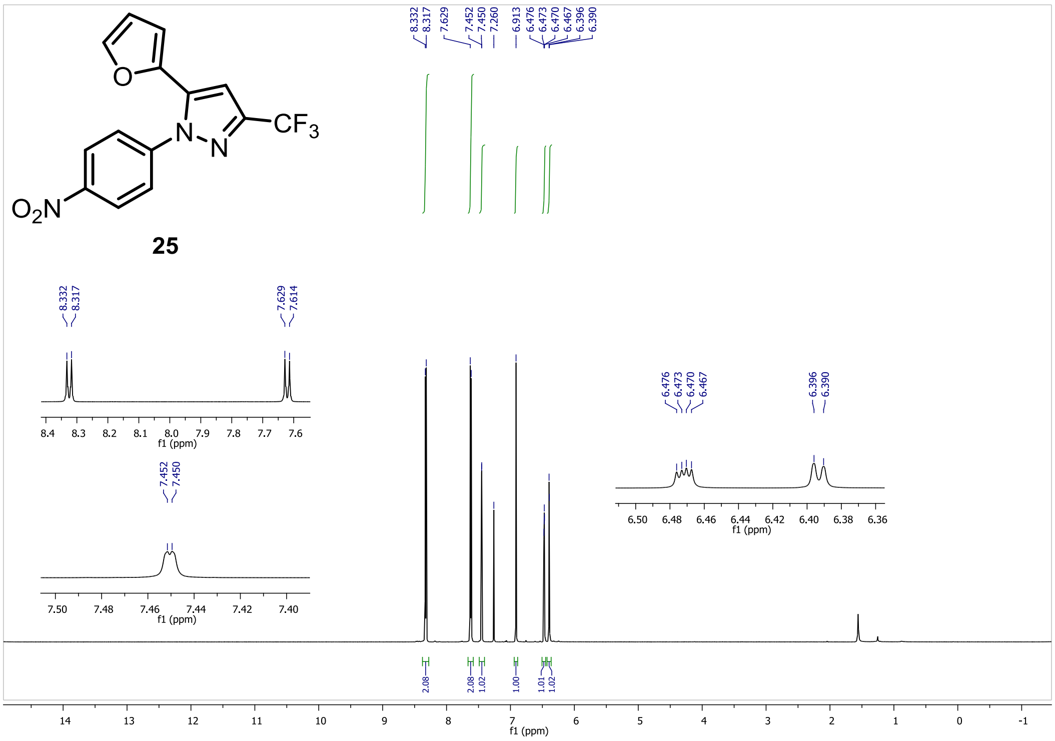

^{13}C NMR, 150 MHz, $CDCl_3$:

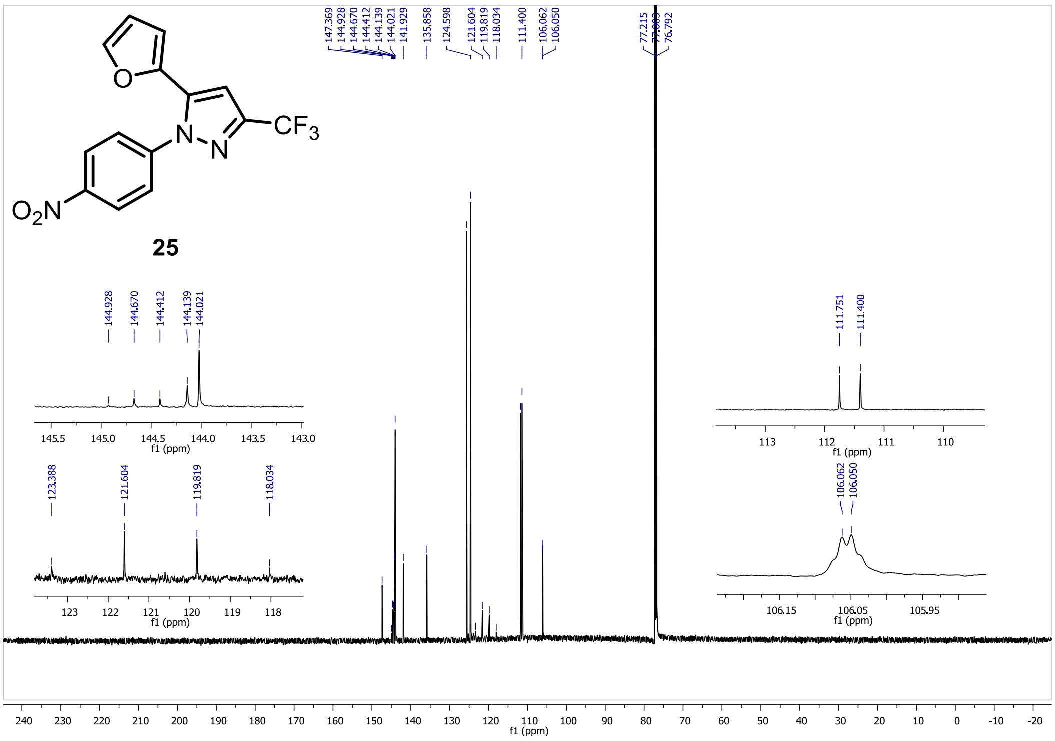

^{19}F NMR, 376 MHz, $CDCl_3$:

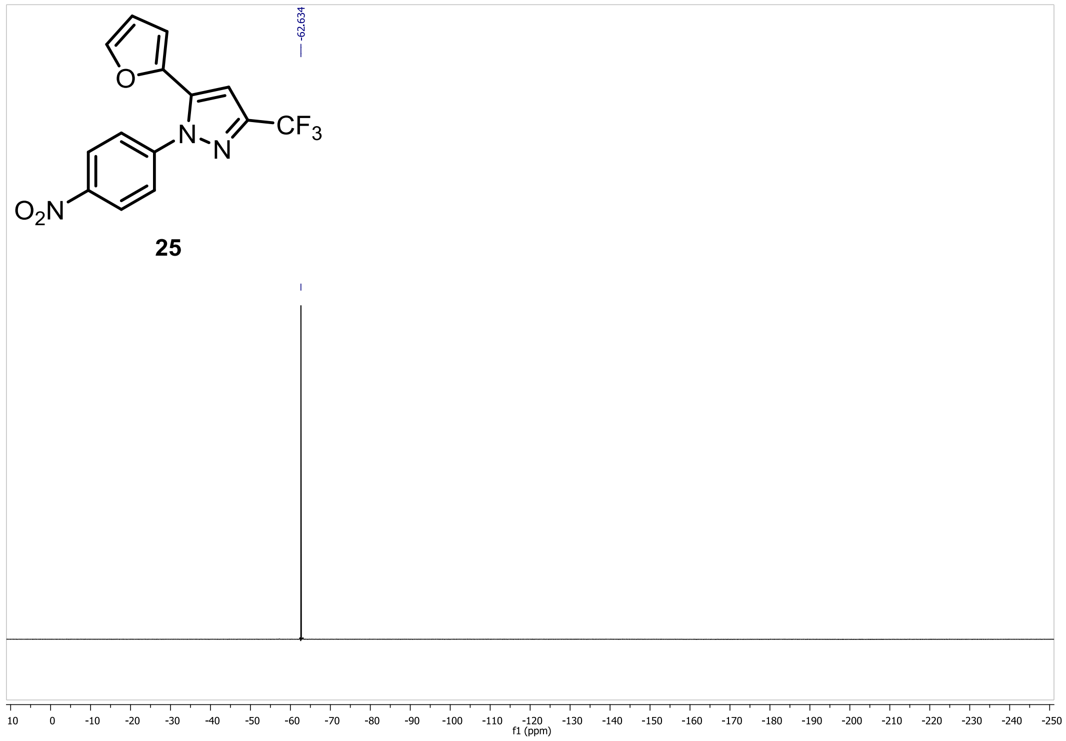

4. References

[1] http://www.uniqsis.com/

[2] http://www.vapourtec.co.uk/home

[3] http://www.knauer-germany.com/en/products/product-types/pumps/

[4] http://uk.mt.com/gb/en/home/products/L1_AutochemProducts/ReactIR/flow-ir-chemis.html

[5] H. Lange, C. F. Carter, M. D. Hopkin, A. Burke, J. G. Goode, I. R. Baxendale and S. V. Ley, *Chem. Sci.*, 2011, **2**, 765-769.

[6] K. Sano and S. Hara, *Heterocycles*, 2010, **80**, 349-357.

[7] N. Joubert, E. Baslé, M. Vaultier and M. Pucheault, *Tetrahedron Lett.*, 2010, **51**, 2994-2997.

[8] M. Zora and A. Kivrak, *J. Org. Chem.*, 2011, **76**, 9379-9390.

[9] A. Alberola, L. C. Bleye, A. González-Ortega, M. L. Sádaba and M. C. Sañudo, *Heterocycles*, 2001, **55**, 331-351.

[10] Y. Schneider, J. Prévost, M. Gobin and C. Y. Legault, *Org. Lett.*, 2014, **16**, 596-599.

[11] R. G. Micetich, *Can. J. Chem.*, 1970, **48**, 2006-2015.

[12] M. Hirano, K. Nakao, S. Nukui and T. Yamagishi, Warner-Lambert Company LLC, Patent US 2004/0019045, 29 January 2004.

[13] D. S. Iyengar, K. K. Prasad and R. V. Venkataratnam, *Tetrahedron Lett.*, 1972, **13**, 3937-3940.

[14] N. Y. Bamaung, A. Basha, S. W. Djuric, E. J. Gubbins, J. R. Luly, N. P. Tu, D. J. Madar, U. Warrior, P. E. Wiedeman, X. Zhou, R. J. Sciotti and F. J. Wagenaar, Abbott Laboratories, Patent US 2001/0044445, 22 November 2001.
